# Treatment of Severe Uncontrolled Chronic Rhinosinusitis with Nasal Polyposis (CRSwNP) with Mepolizumab or Dupilumab: A Preliminary Single-Center Study for Evaluation of Safety and Efficacy

**DOI:** 10.3390/jpm16040224

**Published:** 2026-04-17

**Authors:** Melania Bertolini, Lorenzo Fucci, Luca Guastini, Carlo Conti, Gregorio Santori, Frank Rikki Mauritz Canevari

**Affiliations:** 1 Department of Otolaryngology and Head and Neck Surgery, IRCCS Ospedale Policlinico San Martino, Largo Rosanna Benzi 10, 16132 Genoa, Italy; fuccilorenzo99@gmail.com (L.F.); luca.guastini@unige.it (L.G.); carlo.conti.it@gmail.com (C.C.); frank.canevari@unige.it (F.R.M.C.); 2 Department of Surgical Sciences and Integrated Diagnostics (DISC), University of Genoa, 16132 Genoa, Italy; gregorio.santori@unige.it

**Keywords:** chronic rhinosinusitis with nasal polyposis (CRSwNP), biologic therapies, Mepolizumab, Dupilumab, efficacy, safety

## Abstract

**Background:** The study aims to analyze the safety and efficacy of Mepolizumab and Dupilumab in the treatment of patients affected by severe chronic rhinosinusitis not controlled with nasal polyposis (CRSwNP) from a tertiary care regional referral center, with the aim of improving the concept of personalized medicine. **Methods:** A retrospective study was conducted on 72 adult patients selected for biologic therapy according to EPOS/EUFOREA criteria. The patients received either Mepolizumab or Dupilumab. Primary endpoints were reduction in nasal polyp size, improvement in disease-specific quality of life (sinonasal outcome test-22, visual analog scale), olfactory recovery, and asthma control. Secondary outcomes were the assessment of adverse events. **Results:** Both monoclonal antibodies significantly improved nasal polyps score (NPS), sinonasal outcome test-22 (SNOT-22), and asthma control test (ACT) over time, with no statistically significant differences between Mepolizumab and Dupilumab. In contrast, blood eosinophil counts showed significant differences: Dupilumab was associated with a transient increase in eosinophil levels (absolute Δ = 660.08% Δ = 9%; *p* < 0.001), while Mepolizumab produced a marked reduction (absolute Δ = 192.52% Δ = 2%; *p* < 0.001). Both treatments were well tolerated, with only mild adverse events reported. **Conclusions:** Mepolizumab and Dupilumab are both effective and safe in improving sinonasal symptoms and quality of life in severe uncontrolled CRSwNP. While improvements in NPS, SNOT-22, and ACT scores were comparable, Mepolizumab achieved a significant reduction in eosinophil counts, whereas Dupilumab was associated with faster clinical improvement but a transient eosinophilia. These findings suggest that biologic choice may be guided by individual patient profiles and inflammatory patterns.

## 1. Introduction

Chronic rhinosinusitis (CRS) is defined as a chronic inflammatory condition characterized by nasal congestion, rhinorrhoea, facial pain or pressure, and hyposmia lasting more than 12 weeks that affects between the 5% and 12% in the global population. Historically, it is differentiated into two clinical subtypes, according to the presence or absence of polyps: CRSwNP (CRS with nasal polyposis) and CRSsNP (CRS without nasal polyposis). Beyond anatomical classification, rhinosinusitis should be interpreted as a multifactorial disease driven by both infectious and immunological mechanisms. Allergic inflammation may contribute significantly to sinonasal disease, either independently or in association with infection. The identification of endotypes such as central compartment atopic disease (CCAD), strongly linked to inhalant allergy, further supports the role of atopy in chronic rhinosinusitis and highlights the need for a personalized, mechanism-based diagnostic approach [1]. In recent decades, different studies have focused on the analysis of the immunopathological mechanism, defining three different immunological pathways named as endotypes [2,3,4]. These endotypes have been included in the last European Position Paper on Rhinosinusitis and Nasal Polyps (EPOS) [5], which classified primary CRS according to the presence or absence of type-2 inflammation. Indeed, the EPOS group shifted the management of phenotypical classification towards a new classification based on the disease being localized (often unilateral) or diffuse (always bilateral) and based on the endotype into type 2 or non-type 2 disease. In this scenario, CRSwNP is mainly associated with type-2 inflammation, which entails the production of specific interleukins (IL-3, IL-4 and IL-5) [6,7], as well as the recruitment of eosinophils, basophils and mast cells [4,8].

Clinically, CRSwNP is related to a more severe form that is recalcitrant to the standard therapy, based on topical and/or systemic corticosteroid and surgery treatment, often correlated to other type-2 diseases such as comorbid asthma and/or Exacerbated Respiratory Disease (N-ERD) [9]. Thus, difficult-to-treat patients have a more severe disease requiring high systemic corticosteroid use and/or multiple sinonasal surgeries [10,11]. In recent years, the development of biological target therapies, which restrain the signaling of specific cytokines and interrupt the inflammatory cascade, has provided a new effective option to manage CRSwNP. In Italy, three monoclonal antibodies are now authorized for CRSwNP: anti-IL- 4/IL-13 (Dupilumab), anti-IgE (Omalizumab), and anti-IL-5 (Mepolizumab).

Even though disease-specific consensus and protocols are widespread to guide the treatment with biologic therapy [12,13,14,15], the panorama of biologic therapy continues to increase, leading to difficulty in choosing the best biologic therapy for CRSwNP patients. With the aim of improving the concept of personalized medicine, we analyzed the safety and efficacy of Dupilumab and Mepolizumab in our cohort of patients from a tertiary-care regional referral center.

## 2. Materials and Methods

### 2.1. Study Design

A retrospective single-center observational study in a real-life setting was conducted in the Unit of Otorhinolaryngology—Head and Neck Surgery at the IRCSS Ospedale Policlinico San Martino, Genoa, Italy. Adult patients with severe CRSwNP treated with Dupixent^®^ (Dupilumab, Sanofi/Regeneron Pharmaceuticals, Paris, France/Tarrytown, NY, USA) and Nucala^®^ (Mepolizumab, GlaxoSmithKline, Brentford, United Kingdom) between 1 May 2021 and 31 March 2025 were enrolled. All patients met the eligibility criteria for biologic treatment according to the latest EPOS/EUFOREA [16] criteria and the treatment plan established by the Italian Medicines Agency [17,18]. The requirement for written informed consent was waived because of the retrospective nature of the study. The research was conducted under the approval of the IRCCS Ospedale Policlinico San Martino Institutional Ethics Committee (CER Liguria: 384/2022-DB id 11996), following the principles of the Declaration of Helsinki.

### 2.2. Study Assessment

Before treatment, all patients were investigated in terms of clinical history and habits, comorbidities, presence of asthma, allergies to inhalant and intolerance to non-steroidal anti-inflammatory drugs (NSAID), past surgical history with details on the number of previous functional endoscopic surgeries (FESS), use of nasal corticosteroids, use and number of cycles of systemic corticosteroid (SCS) per year. Diagnostic workup included a nasal rigid endoscopy with 0–30° and 45° rod lenses (Karl Storz^®^ endoscope, Tuttlingen, Germany), evaluating both nasal cavities to measure the nasal polyp score (NPS) and a smell 16-stick identification test (SSIT-16) [19] through the Burghart Odofin Sniffin’ 16-Stick Identification Test^®^. Patients were required to complete subjective questionnaires for assessing their quality of life (QoL) through the overall visual analog scale (VAS), symptoms and the sinonasal outcome test-22 (SNOT-22) [20], and for assessing their control over asthma through the asthma control test (ACT) [21]. A maxillofacial computed tomography (CT) scan was required at the baseline to evaluate the radiological severity of the disease (Lund- Mackay score) [22] and the completeness of previous endoscopic surgeries, when performed, based on the degree of patency of drainage routes of the paranasal sinuses (ACCESS score) [22]. All patients also performed blood tests for evaluation of blood eosinophils and serum Immunoglobulin type E (IgE).

#### 2.2.1. Dupilumab and Mepolizumab Treatment Eligibility Criteria

Based on the therapeutic plan established by the Italian Medicines Agency (AIFA), patients with the following characteristics were considered eligible for treatment: age ≥ 18 years; endoscopic diagnosis of severe CRSwNP; NPS > 5 or SNOT-22 > 50; failure of prior medical treatments due to complications or inefficacy (at least 2 cycles of systemic corticosteroid in the last year) and/or failure of previous surgical treatment (ascertained by the onset of postoperative complications or by lack of therapeutic response). Since the two biologics shared the same criteria of eligibility, patients received Mepolizumab or Dupilumab according to the patient’s preference or the clinician’s personal experience.

#### 2.2.2. Dupilumab and Mepolizumab Administration and Follow-Up

Dupilumab and Mepolizumab were administered with a pre-filled syringe through a subcutaneous injection at a dose of 300 mg once every two weeks and at a dose of 100 mg every month, respectively. The first injection was carried out under medical control at the Unit of Otorhinolaryngology-Head and Neck Surgery at the IRCSS Ospedale Policlinico San Martino, Genoa, Italy. Adverse events were monitored and defined as early or late, if the onset was before or after 30 days of treatment, respectively. Therefore, follow-up visits were conducted at 1, 3, 6, 9 and 12 months. After one year of treatment, patients were followed up every 6 months. Follow-up of all these patients is still ongoing. QoL assessments, ACT, nasal endoscopy, and eosinophilic count were investigated at each follow-up visit. ACT scan control was performed after one year of therapy.

#### 2.2.3. Efficacy of Treatment

To assess the efficacy of treatment, the indications proposed by the previous consensus of EPOS 2020/European Forum for Research and Education in Allergy and Airway Diseases (EUFOREA) [23] were followed. The evaluation of treatment response was based on the following criteria at 6 and 12 months: NPS reduction (at least 1 point); no need for systemic corticosteroids (SCS) or salvage surgery; SNOT-22 reduction (at least <40 points or the minimal clinically important difference (MCID) of 9 (surgical) −12 (medical) if baseline SNOT-22 was <20 points); SSIT-16 improvement from anosmia to hyposmia (at least 6 points); and reduced impact of comorbidities (MCID of ACT of at least 3). Based on the abovementioned criteria, patients were divided into 3 groups: “No–Poor responder” (0–1 criteria met), “Moderate responder” (2–3 criteria met), or “Good–Excellent responder” (4–5 criteria met). Therapy was carried out at 12 months if at least 2 criteria were satisfied.

### 2.3. Statistical Analysis

The results were expressed as mean ± standard deviation (SD), median, counts or percentages. The Shapiro–Wilk test was used to assess the normal distribution of continuous variables and to choose the appropriate test for group comparisons. In the Shapiro–Wilk test, a *p*-value < 0.05 indicates that the data significantly deviates from a normal distribution (reject the null hypothesis of the normal distribution), while a *p*-value > 0.05 suggests the data does not significantly differ from a normal distribution (fail to reject the null hypothesis). The results of the Shapiro–Wilk test were reported for all continuous variables, as well as for each treatment group and each follow-up time-point in the Supplementary Material [SM, Tables S1–S4]. In the SM file, many other descriptive statistics for continuous variables were calculated [95% confidence intervals (95% CI), standard error (SE) of mean, interquartile range (IQR), minimum/maximum value, skewness, SE of skewness, kurtosis, SE of kurtosis]. For each treatment group, a box–violin plot was provided for the main variables evaluated at each time point. The non-parametric Mann–Whitney U test for continuous variables and the chi-square or Fisher’s exact test for binary variables were used to compare the groups. In the SM, both chi-square and Fisher’s exact test have been presented in each contingency table. In each treatment group, a non-parametric one-way ANOVA for repeated measures was performed. The ANOVA models, which were found to be significant at the overall Friedman test, were evaluated for pairwise comparisons by using the post hoc Durbin-Conover test. A Propensity Score Matching (PSM) analysis was performed to reduce a potential selection bias and to balance the observed covariates between treatment groups. In the PSM analysis, the ATT (Average Treatment Effect on the Treated) was used as the target estimand, while the matching was obtained by applying the Greedy method (nearest neighbor approach) without replacement (1:1 matching). A missing imputation procedure was preliminarily carried out for *n* = 3 unavailable data in one of the covariates (eosinophil count before treatment) assumed for the PSM. These missing values were calculated within the Dupilumab group by entering the mean value of the available values in the same variable (mean imputation method) [SM, Tables S5 and S6, Figure S1]. The variables used for matching were entered into a logistic regression model to estimate the propensity score, with treatment assignment serving as the dependent variable and covariates potentially influencing assignment as independent variables. Covariate balance was evaluated with empirical cumulative distribution function (eCDF), absolute standardized mean differences, Kolmogorov–Smirnov statistics, empirical quantile-quantile (eQQ) and density plots. A PS analysis for subclasses was also performed. Logistic regression was used to evaluate putative independent predictors for the occurrence of overall complications. The independent variables with *p*-value < 0.05 or < 0.1 were entered in multivariate regression models. In each logistic regression model, the *β* coefficient and odds ratio (OR) with 95% CI were calculated. The more relevant logistic regression models were also evaluated for overall accuracy, sensitivity and specificity, by providing the corresponding ROC curve with area under the curve (AUC) and cut-off plot. The statistical analyses performed in all enrolled patients (unmatched sample) were replicated in the subsample obtained after PSM analysis (matched sample). In all tests, a two-tailed *p*-value less than 0.05 was considered statistically significant. Statistical analysis was performed using the R environment (version 4.5.2. R Core Team, 2025. R: A language and environment for statistical computing. R Foundation for Statistical Computing, Vienna, Austria; a detailed list of R packages used in this study is reported at the end of the SM file).

## 3. Results

A total of 97 patients were treated with Mepolizumab or Dupilumab. However, 25 patients were excluded from enrolment because they were either lost to follow-up or had started biologic treatment for reasons other than CRSwNP, such as asthma or EGPA. Finally, 41 patients treated with Dupilumab and 31 patients treated with Mepolizumab were retrospectively enrolled. The overall female-to-male ratio was 1.3 and the mean age was 56.5 ± 14.6 years. Fifty-five patients (76.4%) had undergone previous endoscopic sinus surgery, while the mean number of cycles of SCS therapy in the previous year was 6.5 ± 9.4 and 2.0 ± 3.9 with Dupilumab and Mepolizumab, respectively (*p* = 0.024). Forty-eight patients (66.7%) were allergic and 58 (80.6%) had comorbid asthma. A total of thirty patients (41.7%) presented Samter’s triad, consisting of the simultaneous presence of nasal polyps, asthma, and NSAID intolerance, of whom 22 (30.6%) and 8 (11.1%) were from the Dupilumab and Mepolizumab groups, respectively (*p* = 0.029). General demographic and clinical data are presented in Table 1, while Table 2 shows the clinical and radiological scores, blood test results, and endoscopic assessment of NPS collected before starting biological therapy. When comparing patients treated with Dupilumab and Mepolizumab, the Mann–Whitney test revealed significant differences in the number of treatment cycles (*p* = 0.024) and peripheral eosinophil counts (*p* = 0.039), with higher eosinophil levels in the Mepolizumab cohort. The prevalence of NSAID intolerance significantly differed between treatment groups (73.3% in the Dupilumab group vs. 26.7% in the Mepolizumab group; χ^2^ = 5.63, *p* = 0.018). No significant differences were observed for other clinical or radiological parameters (all *p* > 0.05). In the matched sample (*n* = 31 patients for each group) returned by the PSM analysis, no statistical differences were found for the same comparisons shown in Table 1 and Table 2 [SM, pp. 55–56]. In the SM file are reported other comparisons for eosinophil count before treatment [SM, Tables S5–S9, including the missing imputation for n = 3 unavailable data in the Dupilumab group; Figure S1 n = group], NSAID intolerance [SM, Tables S10–S12], ACT [SM, Tables S13–S17] and EPOS [SM, Tables S18–S20]. In the matched sample (n = 31 patients for each group) returned by the PSM analysis, extended descriptive statistics for continuous and categorical variables are reported in the SM file [Tables S21–S23]. No further stratifications in subgroups were performed, considering the overall results of PSM analysis [SM: Section S5, Figures S2–S9], as well as the PSM outputs for subclasses [SM: Figures S10–S12]. In the matched sample, no statistical differences were found for the same comparisons performed in the Table 1 and Table 2 [SM: Tables S24–S26]. In the matched sample we replicated the main analyses performed in the whole sample for eosinophil count [SM, Tables S27–S31, Figure S13], NSAID intolerance [SM: Tables S32–S34], ACT [SM: Tables S35–S39], and EPOS [SM: Tables S40 and S41].

### 3.1. Adverse Events

Five patients, all belonging to the Dupilumab cohort, reported early adverse events [SM: Table S43]: one patient complained of conjunctivitis, one of a fleeting headache, one hands joints pain, one of mild myalgia and one of both headache and conjunctivitis. All symptoms reported were mild, resolved spontaneously and did not lead to treatment discontinuation. Nine patients [SM: Table S43] experienced late-onset complications during treatment: seven in the Dupilumab group and two in the Mepolizumab group. These complications included asthma exacerbations (four patients: three on Dupilumab, one on Mepolizumab), moderate hyper-eosinophilia (two Dupilumab patients), late-onset conjunctivitis (one Dupilumab patient), alopecia (one Mepolizumab patient), and weight gain (one Dupilumab patient). As a result of these adverse events or insufficient clinical response, all nine patients required switching to an alternative biologic therapy. A comparison of overall complications between the treatment groups resulted in almost significant (*p* = 0.057), with 24.4% of complications in the Dupilumab group vs. 6.5% in the Mepolizumab group [SM: Table S44]. Although in the matched sample [SM: Table S45], no statistical significance was found for this same comparison, a lower number of complications was observed in patients assigned to the Mepolizumab group [SM, Table S46].

The univariate logistic regression (LR) was performed in all enrolled patients by entering overall complications as the dependent variable [SM: Table S47]. The LR returned two putative independent predictors [Number of cycles of SCS in the previous year: β = −0.091, OR = 0.913 (95% CI: 0.855–0.975), *p* = 0.007; Access: β = 0.102, OR = 1.108 (95% CI: 1.002–1.225), *p* = 0.046]. In the subsequent multivariate logistic regression model (MLR1) [SM: Table S48], only the number of cycles of SCS in the previous year reached statistical significance (*p* = 0.012). The MLR1 had an accuracy of 0.873 (specificity = 0.417, sensitivity = 0.966, AUC = 0.746) [SM, Figure S15]. The negative β value returned in the LR models for the number of cycles of SCS in the previous year indicates an inverse relationship between the predictor increasing values and the probability of complications. In a second multivariate LR model (MLR2) we also entered the variables with a *p*-value < 0.1 at the univariate analysis [SM: Table S49]. In the MLR2, four potential independent predictors for complications were found [Number of cycles of SCS in the previous year: β = −0.156, OR = 0.855 (95% CI: 0.735–0.996), *p* = 0.044; Access: β = 0.329, OR = 1.389 (95% CI: 1.050–1.838), *p* = 0.021; Group of treatment (target: Dupilumab): β = 3.830, OR = 46.084 (95% CI: 1.080–1966), *p* = 0.045; Gender (target: Female): β = 6.417, OR = 612.195 (95% CI: 3.406–110,022), *p* = 0.002]. The MLR2 had an accuracy of 0.902 (specificity = 0.727, sensitivity = 0.940, AUC = 0.949) [SM, Figure S16]. The replication of univariate logistic regression on the matched sample did not find any putative independent predictor for complications, although relatively high OR occurred for the group of treatment, allergy, gender, NSAID intolerance and allergy, while a *p* < 0.1 was returned by the Lund–Mackay score [SM, Table S50].

### 3.2. Clinical Outcomes

In the Dupilumab cohort, a statistically significant improvement was observed as early as one month for several clinical parameters. The Nasal Polyp Score (NPS) decreased from 5.78 (95% CI: 5.3–6.25) to 3.76 (95% CI: 3.05–4.47), with a *p*-value = 0.003. Similarly, the SNOT-22 score improved from 59.56 (95% CI: 53.23–65.89) to 34.35 (95% CI: 26.97–41.74), and the Visual Analog Scale (VAS) for quality of life improved from 44.71 (95% CI: 39.6–42.8) to 24.8 (95% CI: 19–30.6), both with *p*-values < 0.001. Additionally, the sense of smell significantly improved from 4.14 (95% CI: 3.35–4.94) to 7.53 (95% CI: 6.54–8.53), again with a *p*-value < 0.001. At three months, the Asthma Control Test (ACT) score also showed a significant improvement, rising from 18.26 (95% CI: 16.43–20.13) to 22 (95% CI: 20.64–23.4), with a *p*-value = 0.022. This positive trend was consistently maintained at 6, 9, 12, 18, and 24 months throughout the entire follow-up period.

**Figure 1:**
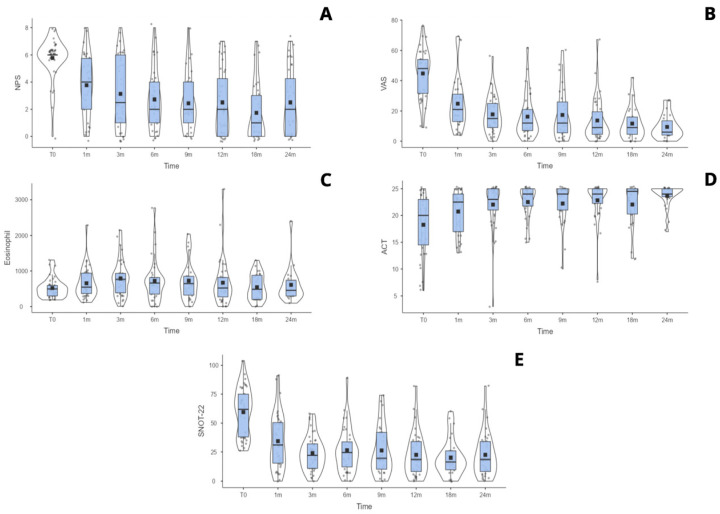
Box–violin plots for Dupilumab main clinical outcomes over time: Nasal Polyp Score (NPS) (**A**); visual analog scale (VAS) (**B**); eosinophil count (cells/µL) (**C**); asthma control test (ACT) (**D**); and sinonasal outcome test-22 (SNOT-22) (**E**).

**Figure 2:**
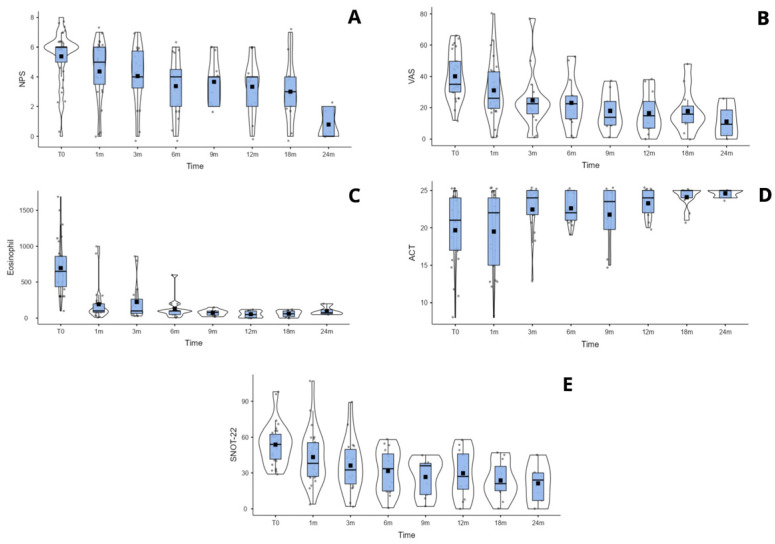
Box–violin plots for Mepolizumab main clinical outcomes over time: nasal polyp score (NPS) (**A**); visual analog scale (VAS) (**B**); eosinophil count (cells/µL) (**C**); asthma control test (ACT) (**D**); and sinonasal outcome test-22 (SNOT-22) (**E**).

In comparison, the Mepolizumab cohort showed a significant improvement beginning at three months. The SNOT-22 score decreased from 53.74 (95% CI: 47.75–59.73) to 36.16 (95% CI: 25.97–46.36), and the VAS improved from 40.11 (95% CI: 34.34–45.89) to 24.62 (95% CI: 15.67–33.57), with *p*-values = 0.047 and 0.028, respectively. A significant reduction in NPS was observed at six months, from 5.38 (95% CI: 4.75–6.02) to 3.37 (95% CI: 2.36–4.38), and this improvement was maintained during subsequent follow-up visits. Eosinophil count and percentage also showed a statistically significant reduction starting from the first month of treatment, with absolute values dropping from 695.49 (95% CI: 563.3–827.67) to 192.55 (95% CI: 89.03–293.01), and percentages decreasing from 9.76% (95% CI: 7.98–11.54%) to 2% (95% CI: 1.5–2.48%), both with *p*-values < 0.001. The trend of the main clinical outcomes for both biologic therapies is shown in Figure 1 and Figure 2. Detailed descriptive statistics for each time-point are reported in the SM [for Figure 1, in SM: Table S3; for Figure 2, in SM: Table S4]. The same box–violin plot in Figure 1 and the related statistics were produced for the Dupilumab group after matching, where *n* = 10 patients of this cohort were discharged from the matched sample [SM, Figure S14, Table S42].

When comparing the two groups, no statistically significant differences were found in terms of quality of life, except at the three-month mark, when patients treated with Dupilumab had significantly lower SNOT-22 scores than those receiving Mepolizumab [23.95 (95% CI: 19.43–28.46) vs. 36.17 (95% CI: 25.97–46.36); *p*-value = 0.045]. There were no significant differences between the two groups in terms of sense of smell, NPS, or ACT scores. Dupilumab appeared to act faster than Mepolizumab (1-month ΔSNOT-22: −25.20 vs. −10.44; ΔVAS: −19.86 vs. −9.04; SSIT-16: +3.39 vs. +0.45; ΔNPS: −2.02 vs. −1.02; ΔACT: +2.43 vs. −0.17, respectively).

A statistically significant difference was observed in eosinophil counts and percentages as early as the first month of treatment. The absolute eosinophil count increased to 660.08 (95% CI: 523.37–796.79) in the Dupilumab group compared with a reduction to 192.52 (95% CI: 89.03–296.01) in the Mepolizumab group, and the percentage increased to 9.2% (95% CI: 7.4–11%) versus a 2% decrease (95% CI: 1.5–2.48%), both with *p*-values < 0.001.

**Figure 3:**
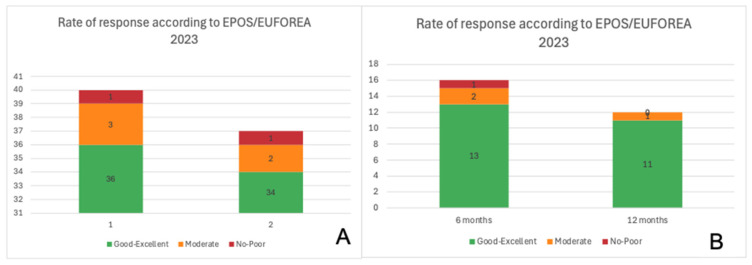
Response rate according to EPOS/EUFOREA 2023 to Dupilumab (**A**) and to Mepolizumab (**B**).

Finally, the rate of excellent response according to the EPOS/EUFOREA 2023 guidelines was 90% at 6 months and 91.8% at 12 months for Dupilumab, and 81% at 6 months and 91.6% at 12 months for Mepolizumab, as shown in Figure 3.

## 4. Discussion

Over the past decade, considerable efforts have been made to better understand the aetiopathogenesis of CRSwNP. The complex cascade of inflammatory cytokines and cells involved in type-2 inflammation has led to the development of monoclonal antibodies that selectively bind specific receptors and molecules, thereby interrupting the never-ending recalcitrant inflammation [24]. Inflammatory processes affecting the paranasal sinuses are not exclusively related to infectious etiologies. Allergic rhinitis and allergic sinusitis can trigger sinonasal inflammation even in the absence of bacterial pathogens, generating clinical manifestations that may resemble those observed in infectious sinusitis. This overlap highlights the importance of considering allergic and immunological mechanisms, including both IgE-mediated and non-IgE-mediated hypersensitivity reactions, during the diagnostic evaluation of patients with persistent or recurrent sinonasal symptoms [25].

Dupilumab is a fully human monoclonal antibody (Mab) that binds to the shared receptor’s subunit called IL-4alfa, inhibiting the IL-4 and IL-13 pathways [26]. In Italy, it is approved for the treatment of uncontrolled CRSwNP in adults, as well as for some allergic diseases, including moderate-to-severe asthma, moderate-to-severe atopic dermatitis, and eosinophilic esophagitis [17,27]. Mepolizumab is an IL-5 Mab antagonist that was initially approved for severe asthma with eosinophilic inflammation, receiving approval in 2023 as a treatment of CRSwNP in adults beyond as a treatment for moderate-to-severe asthma [18,28,29]. Several studies have evaluated the efficacy and safety of these Mabs in the management of CRSwNP [30,31,32,33,34]. These studies consistently reported encouraging results, including improvement in patients’ quality of life (QoL), reduction in polyps’ size and restoration of olfactory function.

Our findings are consistent with the existing literature, further confirming the efficacy and safety of these therapies in patients with CRSwNP. Both Dupilumab and Mepolizumab demonstrated significant improvements in clinical parameters (including NPS, SNOT-22, VAS, sense of smell, and ACT) within the first months of treatment. Moreover, when comparing the two Mabs, our intention was not to highlight limitations but rather to emphasize their differences and strengthen their respective advantages, with the ultimate goal of optimizing treatment personalization according to patient characteristics. However, biologic selection remains a clinical challenge. Over the past decade, several meta-analyses of randomized controlled trials (RCTs) have indirectly compared Mabs, consistently concluding that Dupilumab demonstrates superior efficacy [35,36,37,38,39]. On the other hand, heterogeneity in the inclusion criteria across various RCTs analyzed may introduce bias in the results. Oykhman et al. [36] confirmed that both therapies are effective in improving symptoms, while Dupilumab provides greater benefits in terms of olfactory recovery, reduction in SCS use, decrease in NPS and, consequently, a reduction in the need for surgery. By contrast, Mepolizumab appeared to be slightly less effective in reducing NPS, decreasing SCS requirements and restoring the sense of smell [40]. Lipworth et al. [39] reported the greatest improvements in NPS, nasal congestion score (NCS) and sense of smell, along with a reduction in SCS use and need for surgery, with Anti-TSLP (Tezepelumab) or anti- IL4Ra (Dupilumab). However, these conclusions were based on inspection and comparison of 95% CI derived from the forest plots of phase-III RCTs, and the authors did not formally assess the heterogeneity or weighting for each trial. Conversely, Chen et al. [37] conducted a quality assessment including six studies. Regarding long-term efficacy (NPS, SNOT-22, VAS), Dupilumab ranked highest, followed by Mepolizumab. Nevertheless, their findings were limited by the small number of studies available, the persistent heterogeneity among trials and the lack of data regarding personalized treatment strategies, based on patients’ baseline immunological or clinical profiles. Conversely, in our analysis, detailed information regarding patients’ baseline immunological and clinical profiles was available. In our study population, patients receiving the two Mabs differed in terms of ASA syndrome, number of SCS cycles per year and eosinophils. Based on these findings, we preferred anti-IL-5 therapy in patients with higher blood eosinophil levels at baseline. This trend is consistent with previous scientific evidence suggesting a possible association of Dupilumab with the so-called eosinophil escape in peripheral blood, which may require serial monitoring in patients with baseline blood eosinophil levels >1000/mL [41]. Other meta-analyses of real-world studies (RWSs) have failed to directly compare these Mabs, given the significant baseline differences and unequal sample sizes across treatment groups [42]. Some RWS evaluated the efficacy of different biologic therapies but were again unable to draw a significant conclusion from direct comparison because the treatment cohorts varied in baseline characteristics [43]. Recently, an Indirect Treatment Comparisons (ITC) study confirmed the superiority of Dupilumab with respect to SNOT-22, VAS, NPS, need for surgery, SCS use and olfactory function [44]. This analysis included three RCTs considered similar in both study design and outcome definitions, although inclusion criteria differed slightly between the enrolled trials.

In our study, no statistically significant differences were observed in terms of QoL, sense of smell, NPS and ACT. However, consistent with previous reports, Dupilumab appeared to act faster than Mepolizumab. Simon Carney et al. [45] investigated why Mepolizumab and Benralizumab, which target IL5 and IL5R directly, fail to achieve better results in terms of treatment response. The explanation may lie in the fact that eosinophil proliferation, while predominantly driven by IL-5, can also be stimulated by other interleukins such as IL-33, thereby contributing to the reduced responsiveness to therapy. However, our analysis revealed a statistically significant discrepancy in eosinophil count, with a greater reduction observed in the Mepolizumab cohort, and a slight but transient increase in the Dupilumab cohort. Several pieces of evidence supported the use of Mepolizumab in CRSwNP patients with persistent eosinophilia (>1500 cells/μL) or with associated systemic manifestations. Some studies have reported that Dupilumab may increase the risk of activating or worsening systemic eosinophilic diseases, such as eosinophilic granulomatosis with polyangiitis (EGPA), in patients with pre-existing hyper-eosinophilia (eosinophils > 1500 cells/μL) [46]. Dupilumab blocks IL-4Rα, thereby inhibiting IL-4 and IL-13 signaling, but does not interfere with IL-5. Consequently, eosinophils’ migration into tissues is impaired, leading to their accumulation in peripheral blood, resulting in blood hyper-eosinophilia. By contrast, Mepolizumab showed a reduction in blood eosinophils by 83% and disease flare-ups by 69% [28]. Higo et al. [47] analyzed 27 patients with severe asthma, many of whom had already been treated with Mepolizumab or Benralizumab, and were switched to Dupilumab (without a washout period). The switch to Dupilumab resulted in significant benefits, including improved FEV1, ACT scores, an 87% reduction in nasal symptoms, and good tolerability, with transient hyper-eosinophilia occurring in approximately 30% of patients. Similarly, Rosso et al. [48], reported that Dupilumab was more effective than Mepolizumab in reducing IgE levels and in improving SNOT-22, NPS, olfaction, and ACT scores within 3–6 months. Collectively, these studies support a treatment strategy favoring Mepolizumab in patients with eosinophilic asthma or multiple comorbidities, without significant clinical manifestations affecting the paranasal sinuses.

Adverse reactions should also be considered when choosing a biological drug. Although severe adverse events related to Dupilumab-induced hyper-eosinophilia are extremely rare [49], their potential occurrence cannot be completely disregarded. This consideration may favor the use of mepolizumab in selected patients, as it offers a more reassuring safety profile with respect to eosinophil-driven complications. It is important to remember that we are dealing with chronic rhinosinusitis with nasal polyps, a condition that, while disabling, does not usually represent a life-threatening disease. Therefore, exposing even a single patient to the risk of a severe, potentially fatal complication could be seen as disproportionate.

Our study did not show significant differences regarding the incidence of early/late complications between the Dupilumab and Mepolizumab groups, although a relatively higher number of complications occurred in patients assigned to Dupilumab treatment. On the other hand, Qingwu et al. [37] reported in a network meta-analysis a higher risk of adverse events (AEs) with Mepolizumab. In addition, our study observed a low incidence of severe adverse reactions requiring cessation of the treatment. According to Taylor J Stack et al. [50], the most common adverse reactions associated with Dupilumab administration are ophthalmological and dermatological, followed by injection-site reactions. In contrast, the main adverse reactions related to Mepolizumab are primarily pulmonary. Other studies have reported similar findings [51]. The underlying reason for these reactions remains unclear, but one possible explanation is that certain drugs are preferentially used in patients with severe and complex dermatological conditions (in the case of Dupilumab) and pulmonary conditions (in the case of Mepolizumab), respectively. Real-world observational studies of patients switching biologic therapy for severe CRSwNP provide additional insights into differences in biologic efficacy. A recent study of 225 patients assessed switching patterns in biologic treatment for severe CRSwNP and found that lack of efficacy was the most common reason for switching from Mepolizumab and Omalizumab, whereas adverse effects were the main reason for switching from Dupilumab [52]. The absence of head-to-head studies directly comparing the two biologics still precludes definitive declare conclusions regarding the superiority of one biologic over the others. To date, no completed, head-to-head studies comparing the available or emerging biologics for treatment of CRSwNP exist, although several are ongoing (NCT04998604, NCT05942222).

In addition, robust cost-effectiveness data specific to CRSwNP are still limited, and further studies are needed to better define the economic sustainability of long-term biologic therapy and to guide resource allocation in clinical practice [31].

This study has several limitations, including its retrospective single-center design, small sample size, non-randomized treatment allocation, and lack of standardization for concomitant treatments, which may have introduced selection bias and reduced generalizability. It is important to remember that baseline differences between the two treatment groups should be carefully considered when interpreting the results. In particular, variations in the number of prior systemic corticosteroid (SCS) courses and baseline peripheral blood eosinophil counts may reflect differences in disease severity and inflammatory profile. These factors could have influenced treatment response and therefore may act as potential confounders in the comparison between the two monoclonal antibody groups. Another important consideration to take into account is the relatively small sample size that may have limited the statistical power of the study, potentially leading to an underestimation of clinically relevant differences between treatments. For this reason, the results should be interpreted with caution and considered exploratory.

In the matched sample obtained by the propensity score analysis, no statistically significant difference was found between treatment, although several clinical differences were observed anyway. Follow-up was limited to 24 months, although data for some Dupilumab patients were collected up to 36 months, so long-term efficacy and safety remain uncertain. In our study, subgroup analysis (e.g., for multiple comorbidities) would not have been reliable due to the small number of patients and events in the unmatched/matched sample. Finally, the absence of a head-to-head randomized comparison prevents firm conclusions on the relative superiority of Dupilumab vs. Mepolizumab.

## 5. Conclusions

This real-world study confirmed the efficacy of self-administered Dupilumab and Mepolizumab as adjunctive therapy to intranasal corticosteroids (INCS) in patients with uncontrolled severe CRSwNP. Dupilumab appeared to provide faster improvement and was associated with a transient increased of blood eosinophilic levels.

Overall, the choice between these treatments should be individualized, considering patient characteristics, eosinophil levels and comorbid asthma.

It is also important to note that biologics do not replace intranasal corticosteroids, and there is currently no clear indication of when to discontinue treatment, which is usually continued as long as it remains effective and reimbursed.

In conclusion, both drugs represent valid therapeutic options, and their use should be adapted to the clinical profile of each patient, but several questions remain open:

- Identification of independent predictors of response timing to determine the optimal window for performing salvage surgery. Tailoring therapy on an individual basis by identifying factors that modulate response over time would be desirable.
- Further studies could provide more robust data on adverse effects by focusing specifically on the CRSwNP population.
- Evaluation of the safety of Mabs during pregnancy.
- Assessment of strategies for treatment discontinuation or dose reduction.

## Figures and Tables

**Table 1:** Demographic data of patients included in the study.

Variable		Overall (n = 72)	Dupilumab (n = 41)	Mepolizumab (n = 31)	*p*-Value *
Patients			41/72 (56.9%)	31/72 (43.0%)	0.247
Age		56.5 ± 14.6	56.2 ± 14.2	57.1 ± 15.2	0.95
Gender	Male	31 (43.1%)	22 (53.6%)	9 (29%)	0.054
	Female	41 (56.9%)	19 (46.4%)	22 (71%)	
Previous endoscopic sinus surgery	Yes	55 (76.4%)	35/41 (85.3%)	20/31(64.5%)	0.052
	No	17 (23.6%)	6/41 (16.7%)	11/31 (35.5%)	
Number of cycles of SCS in the previous year			6.5 ± 9.4	2.0 ± 3.9	**0.024**
Allergy	Yes		28 (68.3%)	19 (61.3%)	0.80
	No		13 (31.7%)	12 (38.7%)	
Asthma	Yes	58 (80.6%)	33 (80.5%)	25 (80.6%)	1.00
	No	14 (19.4%)	8 (19.5%)	6 (19.4%)	
NSAID intolerance	Yes	30 (41.1%)	22 (53.7%)	8 (25.8%)	**0.029**
	No	42 (58.9%)	19 (46.3%)	23 (74.2%)	

Results represent the mean ± standard deviation and number of events (%) for continuous and categorical variables, respectively. SCS: systemic corticosteroid; NSAID: nonsteroidal anti-inflammatory drug. * *p*-value refers to the comparison between the Dupilumab and Mepolizumab cohorts.

**Table 2:** Clinical and radiological scores, blood test results, and endoscopic assessment of NPS were collected before starting biological therapy.

Variable *	Dupilumab (n = 41)	Mepolizumab (n = 31)	*p*-Value
NPS	5.78 ± 1.56 (6.0)	5.39 ± 1.80 (6.0)	0.287
SNOT-22	59.56 ± 20.66 (62.0)	53.74 ± 17.01 (54.0)	0.150
SSIT-16	4.15 ± 2.59 (3.0)	5.07 ± 2.28 (5.0)	0.079
IgE Tot (kU/L)	315.18 ± 557.48 (173.5)	362.07 ± 595.39 (133.5)	0.748
Eosinophil count (cells/µL)	524.53 ± 275.84 (500)	695.48 ± 375.49 (650)	**0.039**
Eosinophil (%)	8.45 ± 5.52 (7.1)	9.77 ± 4.89 (8.4)	0.161
ACT	18.29 ± 5.58 (20.0)	19.65 ± 4.72 (21.0)	0.352
VAS	44.71 ± 16.08 (48.0)	40.12 ± 15.03 (35.0)	0.253
Lund Mackay	18.95 ± 4.34 (20.0)	18.37 ± 4.78 (19.5)	0.520
Access	10.61 ± 7.84 (7.0)	11.50 ± 9.11 (10.0)	0.916

* The results of each variable are expressed as mean ± SD (median). SSIT-16: Sniffin’ Sticks-16 Identification Test; SNOT-22: Sinonasal Outcome Test-22; ACT: Asthma Control Test; VAS: Visual Analog Scale; NPS: Nasal Polyp Score.

## Data Availability

The original contributions presented in this study are included in the article/Supplementary Material. Further inquiries can be directed to the corresponding author.

## Supplementary Materials

The following supporting information can be downloaded at: https://www.mdpi.com/article/10.3390/jpm16040224/s1, Figure S1: Histogram with density plot for “Eosinophil_or” (original data, with n = 3 missing values in the Dupilumab group) and “Eosinophil_mi” (data after mean imputation for the missing cases in the Dupilumab group) variables, in both Dupilumab and Mepolizumab group; Figure S2: Covariate balance for the multivariate PS model (nearest neighbor approach) without replacement (1:1 matching); Figure S3: Empirical Quantile-Quantile (eQQ) plots for “Age” and “Allergy” variables before and after matching; Figure S4: Empirical Quantile-Quantile (eQQ) plots for “Asthma” and “SNOT.22” variables before and after matching; Figure S5: Empirical Quantile-Quantile (eQQ) plots for “NSAID_intol” and “NPS” variables before and after matching; Figure S6: Empirical Quantile-Quantile (eQQ) plots for “Previous_Surg” variable before and after matching; Figure S7: Empirical Quantile-Quantile (eQQ) plots for “Eosinophil_dic” variable before and after matching; Figure S8: Empirical Quantile-Quantile (eQQ) plots for “Smoke” variable before and after matching; Figure S9: Density plots for each continuous covariate entered in the PS model. The x-axis displays the covariate values, and the y-axis displays the density of the sample at that covariate value. The black line corresponds to the Mepolizumab group and the gray line to the Dupilumab group; Figure S10: Standardized mean differences/covariate before/after matching and balance for each subclass; Figure S11: Density plots for each continuous covariate entered in the PS model (Subclass 1). The x-axis displays the covariate values, and the y-axis displays the density of the sample at that covariate value. The black line corresponds to the Mepolizumab group and the gray line to the Dupilumab group; Figure S12: Density plots for each continuous covariate entered in the PS model (Subclass 2). The x-axis displays the covariate values, and the y-axis displays the density of the sample at that covariate value. The black line corresponds to the Mepolizumab group and the gray line to the Dupilumab group; Figure S13: Histogram with density plot for “Eosinophil_or” (original data, with n = 3 missing values in the Dupilumab group) and “Eosinophil_mi” (data after mean imputation for the missing cases in the Dupilumab group) variables, in both Dupilumab and Mepolizumab group of the matched sample; Figure S14: Box-violin plots for Dupilumab main clinical outcomes over time for the n = 31 patients selected by the propensity score analysis: Nasal Polyp Score (NPS) (A); Visual Analogue Scale (VAS) (B); Eosinophil count (cells/µL) (C); Asthma Control Test (ACT) (D); sinonasal outcome test-22 (SNOT-22) (E); Figure S15: ROC curve (A), cut-off plot (B), classification table and predictive measures of the MLR1; Figure S16: ROC curve (A), cut-off plot (B), classification table and predictive measures of the MLR2; Table S1: Extended descriptive statistics for continuous variables (all cases; Part I/Part II); Table S2: Extended descriptive statistics for continuous variables, grouped for Dupilumab and Mepolizumab (Part I/Part II/Part III); Table S3: Extended descriptive statistics for continuous variables at each time-point of Figure 1 (Dupilumab); Table S4: Extended descriptive statistics for continuous variables at each time-point of Figure 2 (Mepolizumab); Table S5: Extended descriptive statistics for the “Eosinophil_or” and “Eosinophil_mi” variables (all cases); Table S6: Mann-Whitney test performed by comparing Eosinophil count (cells/µL) in Dupilumab vs. Mepolizumab group (“Eosinophil_or”: raw data; “Eosinophil_mi”: data after mean imputation for n = 3 missing values in the Dupilumab group); Table S7: Frequency statistics and binomial test for the “Eosinophil_dic” variable; Table S8: Comparison between Dupilumab vs. Mepolizumab for “Eosinophil_dic” variable; Table S9: Comparison between the main continuous variables by stratifying for absence (No)/presence (Yes) of eosinophil count > 600 cells/µL; Table S10: Comparison between Dupilumab vs. Mepolizumab for NSAID intolerance; Table S11: Comparison between the main continuous variables by stratifying for absence (No)/presence (Yes) of NSAID intolerance; Table S12: Comparison between absence/presence of NSAID intolerance and absence/presence of asthma; Table S13: Frequency statistics for the “Asthma_control” variable; Table S14: Comparison between Dupilumab vs. Mepolizumab for quality of asthma control; Table S15: Comparison between absence/presence of complications and quality of asthma control; Table S16: General one-way non-parametric ANOVA, using “Asthma control” as grouping variable; Table S17: The post-hoc pairwise comparisons for significant variables at the general one-way non-parametric ANOVA model for “Asthma control”; Table S18: Frequency statistics for the “EPOS_SIX” and “EPOS_TWELVE” variables; Table S19: Comparison between Dupilumab vs. Mepolizumab for “EPOS_SIX” variable; Table S20: Comparison between Dupilumab vs. Mepolizumab for “EPOS_TWELVE” variable; Table S21: Descriptive statistics for continuous variables of the matched sample (all cases; Part I/Part II); Table S22: Descriptive statistics for continuous variables of the matched sample, by grouping for Dupilumab and Mepolizumab (Part I/Part II/Part III); Table S23: Descriptive statistics for categorical variables of the matched sample (Part I/Part II/Part III); Table S24: Comparison between continuous variables by stratifying for Dupilumab vs. Mepolizumab in the matched sample; Table S25: Recalculated Table 1 of the paper in the matched sample; Table S26: Recalculated Table 2 of the paper in the matched sample; Table S27: Extended descriptive statistics for the Eosinophil-related continuous variables in the matched sample; Table S28: Mann-Whitney test performed by comparing Eosinophil count (cells/µL) in Dupilumab vs. Mepolizumab group (“Eosinophil_or”: raw data; “Eosinophil_mi”: data after mean imputation for n = 3 missing values in the Dupilumab group) in the matched sample; Table S29: Frequency statistics and binomial test for the “Eosinophil_dic” variable in the matched sample; Table S30: Comparison between Dupilumab vs. Mepolizumab for “Eosinophil_dic” variable in the matched sample; Table S31: Comparison between the main continuous variables by stratifying for absence (No)/presence (Yes) of eosinophil count > 600 cells/µL in the matched sample; Table S32: Comparison between the main continuous variables by stratifying for absence (No)/presence (Yes) of NSAID intolerance in the matched sample; Table S33: Comparison between absence/presence of NSAID intolerance and absence/presence of asthma in the matched sample; Table S34: Frequency statistics for the “Asthma_control” variable in the matched sample; Table S35: Comparison between Dupilumab vs. Mepolizumab for quality of asthma control in the matched sample; Table S36: Comparison between absence/presence of complications and quality of asthma control in the matched sample; Table S37: General one-way non-parametric ANOVA, using “Asthma control” as grouping variable in the matched sample; Table S38: The post-hoc pairwise comparisons for significant variables at the general one-way non-parametric ANOVA model for “Asthma control” in the matched sample; Table S39: Frequency statistics for the “EPOS_SIX” and “EPOS_TWELVE” variables in the matched sample; Table S40: Comparison between Dupilumab vs. Mepolizumab for “EPOS_SIX” variable in the matched sample; Table S41: Comparison between Dupilumab vs. Mepolizumab for “EPOS_TWELVE” variable in the matched sample; Table S42: Extended descriptive statistics for continuous variables at each time-point of Figure S14; Table S43: Number of complications observed in the unmatched sample; Table S44 (Part I): Comparison between Dupilumab vs. Mepolizumab for early complications in the unmatched sample; Table S44 (Part II): Comparison between Dupilumab vs. Mepolizumab for late complications in the unmatched sample; Table S44 (Part III): Comparison between Dupilumab vs. Mepolizumab for overall complications (early and/or late) in the unmatched sample; Table S45: Number of complications observed in the matched sample; Table S46 (Part I): Comparison between Dupilumab vs. Mepolizumab for early complications in the matched sample; Table S46 (Part II): Comparison between Dupilumab vs. Mepolizumab for late complications in the matched sample; Table S46 (Part III): Comparison between Dupilumab vs. Mepolizumab for overall complications (early and/or late) in the matched sample; Table S47: Univariate logistic regression models performed with overall complications as dependent variable in the unmatched sample; Table S48: Multivariate logistic regression model (MLR1) performed with overall complications as dependent variable in the unmatched sample, by entering the significative variables at the univariate analysis; Table S49: Multivariate logistic regression model (MLR2) performed with overall complications as dependent variable in the unmatched sample, by entering the variables with P value <0.1 at the univariate analysis; Table S50: Univariate logistic regression models performed with overall complications as dependent variable in the matched sample

## Abbreviations

The following abbreviations are used in this manuscript:

CRSwNP	Chronic Rhinosinusitis with Nasal Polyposis
NPS	Nasal Polyp Score
VAS	Visual Analog Scale
SNOT-22	Sinonasal outcome test-22
ACT	Asthma Control Test
SSIT-16	Sniffin’ Sticks 16 Identification Test
