# Supplementary material for: Treatment of Severe Uncontrolled Chronic Rhinosinusitis with Nasal Polyposis (CRSwNP) with Mepolizumab or Dupilumab: A Preliminary Single-Center Study for Evaluation of Safety and Efficacy
Source: J Pers Med. 2026 Apr 17;16(4):224. doi: 10.3390/jpm16040224 (PMC13118264; doi:10.3390/jpm16040224)
Supplement: Supplementary file 1 [file jpm-16-00224-s001.zip › jpm-4188212-supplementary.pdf]

TREATMENT OF SEVERE CHRONIC RHINOSINUSITIS NOT CONTROLLED WITH NASAL POLYPOSIS (CRSWNP) WITH MEPOLIZUMAB OR DUPILIMAB: A PRELIMINARY SINGLE-CENTER STUDY FOR EVALUATION OF SAFETY AND EFFICACY

SUPPLEMENTARY MATERIAL

Section S1. Extended descriptive statistics for continuous variables (all cases)

Table S1 – Extended descriptive statistics for continuous variables (all cases; **Part I**).

	Age	NPS	SNOT-22*	SSIT-16*	IgE_Tot	Eosinophil**
**Mean**	56.917	5.611	57.056	4.542	335.502	601.333
**Std. error mean**	1.709	0.196	2.270	0.293	73.551	40.098
**95% CI mean lower bound**	53.567	5.226	52.606	3.967	191.344	522.742
**95% CI mean upper bound**	60.266	5.996	61.505	5.117	479.659	679.925
**Median**	60.000	6.000	58.500	4.500	153.500	510.000
**Standard deviation**	14.501	1.666	19.265	2.489	569.724	333.082
**IQR**	20.250	0.000	29.500	3.000	216.500	420.000
**Range**	63	8	78	10	2883.500	1590.000
**Minimum**	23	0	26	0	2.500	100
**Maximum**	86	8	104	10	2886.000	1690.000
**Skewness**	-0.428	-1.553	0.319	0.397	3.028	1.118
**Std. error skewness**	0.283	0.283	0.283	0.283	0.309	0.289
**Kurtosis**	-0.249	2.716	-0.635	-0.457	9.260	1.157
**Std. error kurtosis**	0.559	0.559	0.559	0.559	0.608	0.570
**Shapiro-Wilk (*W*)**	0.975	0.782	0.964	0.960	0.558	0.915
**Shapiro-Wilk (*P* value)*** **	0.157	< .001	0.036	0.021	< .001	< .001

The "*SNOT-22*" and "*SSIT-16*" variables in several native R outputs (tables and plots) are reported as "*SNOT.22*" and "*SSIT.16*", due to the handling of variable names (characters "_" and "-") by the analysis environment.

** For more details regarding eosinophil count in Dupilumab group, see Section S4.1 (pp. 14-16).

*** The Shapiro-Wilk test evaluates the normal distribution of continuous variables. In this test, a *P* value < 0.05 indicates that the data significantly deviates from a normal distribution (reject the null hypothesis of the normal distribution), while a *P* value > 0.05 suggests the data does not significantly differ from normal distribution (fail to reject the null hypothesis). When a *P* value < 0.05 is returned by the Shapiro-Wilk test, a non-parametric test should be used in the subsequent comparisons.

Table S1 – Extended descriptive statistics for continuous variables (all cases; **Part II**).

	**ACT***	**VAS**	**Lund-Mackay**	**Access**
**Mean**	18.869	42.844	18.704	10.986
**Std. error mean**	0.670	1.963	0.535	0.991
**95% CI mean lower bound**	17.555	38.996	17.656	9.044
**95% CI mean upper bound**	20.183	46.691	19.752	12.927
**Median**	20	45.500	20	7
**Standard deviation**	5.236	15.705	4.505	8.347
**IQR**	7.000	24.000	6.000	14.000
**Range**	19	67	26	24
**Minimum**	6	9	2	0
**Maximum**	25	76	28	24
**Skewness**	-0.726	-0.141	-0.972	0.432
**Std. error skewness**	0.306	0.299	0.285	0.285
**Kurtosis**	-0.341	-0.729	1.617	-1.305
**Std. error kurtosis**	0.604	0.590	0.563	0.563
**Shapiro-Wilk (*W*)**	0.918	0.972	0.935	0.874
**Shapiro-Wilk (*P* value)**	< .001	0.163	0.001	< .001

* For more details regarding ACT, see Section S4.3 (pp. 22-25).

Section S2. Extended descriptive statistics for continuous variables, grouped for Dupilumab and Mepolizumab

Table S2 – Extended descriptive statistics for continuous variables, grouped for Dupilumab and Mepolizumab (**Part I**).

	Group	Age	NPS	SNOT-22	SSIT-16
**Mean**	Dupilumab	56.780	5.780	59.561	4.146
	Mepolizumab	57.097	5.387	53.742	5.065
**Std. error mean**	Dupilumab	2.177	0.243	3.226	0.405
	Mepolizumab	2.774	0.324	3.055	0.409
**95% CI mean lower bound**	Dupilumab	52.514	5.304	53.237	3.352
	Mepolizumab	51.660	4.753	47.754	4.262
**95% CI mean upper bound**	Dupilumab	61.047	6.257	65.885	4.940
	Mepolizumab	62.533	6.021	59.730	5.867
**Median**	Dupilumab	60	6	62	3
	Mepolizumab	61	6	54	5
**Standard deviation**	Dupilumab	13.940	1.557	20.659	2.594
	Mepolizumab	15.443	1.801	17.010	2.279
**IQR**	Dupilumab	18.000	0.000	37.000	3.000
	Mepolizumab	21.500	1.000	21.000	3.000
**Range**	Dupilumab	58	8	78	10
	Mepolizumab	63	8	69	9
**Minimum**	Dupilumab	23	0	26	0
	Mepolizumab	23	0	29	1
**Maximum**	Dupilumab	81	8	104	10
	Mepolizumab	86	8	98	10
**Skewness**	Dupilumab	-0.627	-1.869	0.007	0.545
	Mepolizumab	-0.253	-1.285	0.822	0.403
**Std. error skewness**	Dupilumab	0.369	0.369	0.369	0.369
	Mepolizumab	0.421	0.421	0.421	0.421
**Kurtosis**	Dupilumab	0.092	4.596	-1.027	-0.444
	Mepolizumab	-0.486	1.703	0.964	-0.125
**Std. error kurtosis**	Dupilumab	0.724	0.724	0.724	0.724
	Mepolizumab	0.821	0.821	0.821	0.821
**Shapiro-Wilk (*W*)**	Dupilumab	0.964	0.731	0.946	0.937
	Mepolizumab	0.981	0.845	0.936	0.954
**Shapiro-Wilk (*P* value)**	Dupilumab	0.210	< .001	0.050	0.025
	Mepolizumab	0.839	< .001	0.063	0.195

Table S2 – Extended descriptive statistics for continuous variables, grouped for Dupilumab and Mepolizumab (**Part II**).

	Group	IgE Tot	Eosinophil*	ACT*
**Mean**	Dupilumab	315.182	519.268	18.286
	Mepolizumab	362.073	695.484	19.654
**Std. error mean**	Dupilumab	95.607	46.067	0.943
	Mepolizumab	116.766	67.441	0.926
**95% CI mean lower bound**	Dupilumab	127.796	428.978	16.437
	Mepolizumab	133.216	563.301	17.838
**95% CI mean upper bound**	Dupilumab	502.569	609.558	20.135
	Mepolizumab	590.930	827.667	21.470
**Median**	Dupilumab	173.500	500.000	20
	Mepolizumab	133.500	650.000	21.000
**Standard deviation**	Dupilumab	557.480	283.977	5.581
	Mepolizumab	595.392	375.498	4.724
**IQR**	Dupilumab	211.750	297.500	8.500
	Mepolizumab	209.250	425.000	7.000
**Range**	Dupilumab	2883.500	1309.800	19
	Mepolizumab	2275.400	1590.000	17
**Minimum**	Dupilumab	2.500	0.200	6
	Mepolizumab	11.600	100.000	8
**Maximum**	Dupilumab	2886.000	1310.000	25
	Mepolizumab	2287.000	1690.000	25
**Skewness**	Dupilumab	3.728	1.004	-0.650
	Mepolizumab	2.422	0.877	-0.776
**Std. error skewness**	Dupilumab	0.403	0.383	0.398
	Mepolizumab	0.456	0.421	0.456
**Kurtosis**	Dupilumab	15.009	1.195	-0.593
	Mepolizumab	5.271	0.583	0.045
**Std. error kurtosis**	Dupilumab	0.788	0.750	0.778
	Mepolizumab	0.887	0.821	0.887
**Shapiro-Wilk (*W*)**	Dupilumab	0.508	0.929	0.920
	Mepolizumab	0.603	0.937	0.915
**Shapiro-Wilk (*P* value)**	Dupilumab	< .001	0.018	0.015
	Mepolizumab	< .001	0.067	0.034

* For more details regarding eosinophil count in Dupilumab group and ACT, see Section S4.1 (pp. 14-18) and S4.3 (pp. 22-25), respectively.

Table S2 – Extended descriptive statistics for continuous variables, grouped for Dupilumab and Mepolizumab (**Part III**).

	Group	VAS	Lund-Mackay	Access
**Mean**	Dupilumab	44.711	18.951	10.610
	Mepolizumab	40.115	18.367	11.500
**Std. error mean**	Dupilumab	2.608	0.677	1.224
	Mepolizumab	2.947	0.873	1.664
**95% CI mean lower bound**	Dupilumab	39.598	17.624	8.211
	Mepolizumab	34.339	16.656	8.240
**95% CI mean upper bound**	Dupilumab	49.823	20.278	13.008
	Mepolizumab	45.892	20.078	14.760
**Median**	Dupilumab	48.000	20	7
	Mepolizumab	35.000	19.500	10.000
**Standard deviation**	Dupilumab	16.079	4.336	7.835
	Mepolizumab	15.029	4.781	9.111
**IQR**	Dupilumab	22.500	5.000	13.000
	Mepolizumab	19.750	7.000	15.500
**Range**	Dupilumab	67	22	24
	Mepolizumab	54	20	24
**Minimum**	Dupilumab	9	2	0
	Mepolizumab	12	8	0
**Maximum**	Dupilumab	76	24	24
	Mepolizumab	66	28	24
**Skewness**	Dupilumab	-0.368	-1.592	0.641
	Mepolizumab	0.169	-0.337	0.217
**Std. error skewness**	Dupilumab	0.383	0.369	0.369
	Mepolizumab	0.456	0.427	0.427
**Kurtosis**	Dupilumab	-0.375	4.403	-1.018
	Mepolizumab	-0.949	-0.450	-1.585
**Std. error kurtosis**	Dupilumab	0.750	0.724	0.724
	Mepolizumab	0.887	0.833	0.833
**Shapiro-Wilk (*W*)**	Dupilumab	0.965	0.871	0.871
	Mepolizumab	0.953	0.964	0.861
**Shapiro-Wilk (*P* value)**	Dupilumab	0.274	< .001	< .001
	Mepolizumab	0.279	0.394	0.001

Section S3. Extended descriptive statistics for continuous variables at each time-point

Table S3 – Extended descriptive statistics for continuous variables at each time-point of Figure 1 (Dupilumab)

	Time	NPS	VAS	Eosinophil	ACT	SNOT-22
**Mean**	T0	5.780	44.711	524.526	18.286	59.561
	1m	3.763	24.848	660.081	20.719	34.359
	3m	3.125	17.757	791.389	22.027	23.951
	6m	2.722	16.242	720.650	22.500	26.444
	9m	2.444	17.370	726.231	22.222	26.250
	12m	2.500	13.686	675.767	22.824	22.444
	18m	1.741	11.630	549.192	22.038	20.107
	24m	2.500	9.435	614.211	23.652	22.444
**Std. error mean**	T0	0.243	2.608	44.747	0.943	3.226
	1m	0.361	2.978	69.748	0.710	3.769
	3m	0.399	1.990	85.951	0.703	2.304
	6m	0.366	2.493	102.300	0.516	3.173
	9m	0.411	3.223	105.661	0.725	4.026
	12m	0.387	2.404	107.981	0.590	3.092
	18m	0.422	2.034	71.370	0.833	3.015
	24m	0.387	1.677	117.305	0.438	3.092
**95% CI mean lower bound**	T0	5.304	39.598	436.823	16.437	53.237
	1m	3.056	19.011	523.377	19.327	26.972
	3m	2.343	13.856	622.927	20.648	19.435
	6m	2.004	11.356	520.145	21.489	20.226
	9m	1.639	11.054	519.140	20.802	18.359
	12m	1.741	8.975	464.128	21.667	16.384
	18m	0.913	7.643	409.309	20.406	14.198
	24m	1.741	6.147	384.297	22.793	16.384
**95% CI mean upper bound**	T0	6.257	49.823	612.229	20.135	65.885
	1m	4.470	30.686	796.786	22.111	41.746
	3m	3.907	21.658	959.851	23.406	28.468
	6m	3.440	21.129	921.155	23.511	32.663
	9m	3.250	23.687	933.322	23.643	34.141
	12m	3.259	18.397	887.405	23.980	28.505
	18m	2.568	15.616	689.076	23.671	26.017
	24m	3.259	12.722	844.124	24.511	28.505
**Median**	T0	6	48.000	500.000	20	62
	1m	4.000	21	550.000	22.500	31
	3m	2.500	15	765.000	23	22
	6m	2.000	12	665.000	24.000	24.500
	9m	2	12	650.000	24	19.500
	12m	2.000	9	525.000	24.000	18.500

	Time	NPS	VAS	Eosinophil	ACT	SNOT-22
	18m	1	9	490.000	24.500	16.500
	24m	2.000	6	460.000	24	18.500
**Standard deviation**	T0	1.557	16.079	275.840	5.581	20.659
	1m	2.223	17.110	424.263	4.018	23.538
	3m	2.524	12.107	515.708	4.278	14.755
	6m	2.199	14.322	596.509	2.918	19.036
	9m	2.136	16.745	538.766	3.766	21.303
	12m	2.324	14.220	647.885	3.442	18.554
	18m	2.194	10.569	363.919	4.247	15.954
	24m	2.324	8.044	511.320	2.102	18.554
**IQR**	T0	0.000	22.500	297.500	8.500	37.000
	1m	3.750	18.000	570.000	7.000	35.000
	3m	5.000	16.000	542.500	4.000	21.000
	6m	3.000	14.000	460.000	3.250	21.500
	9m	3.000	20.500	525.000	4.000	31.750
	12m	4.250	15.000	542.500	2.750	25.750
	18m	3.000	11.500	672.500	4.750	16.250
	24m	4.250	9.500	440.000	1.500	25.750
**Range**	T0	8	67	1120.000	19	78
	1m	8	65	2171.000	12	91
	3m	8	56	2140.000	22	58
	6m	8	62	2769.430	10	89
	9m	8	60	2038.000	15	74
	12m	7	67	3296.400	17	82
	18m	7	42	1300.000	13	60
	24m	7	27	2300.000	8	82
**Minimum**	T0	0	9	190.000	6	26
	1m	0	4	119.000	13	0
	3m	0	0	10.000	3	0
	6m	0	0	0.570	15	0
	9m	0	0	2.000	10	0
	12m	0	0	3.600	8	0
	18m	0	0	0.000	12	0
	24m	0	0	100.000	17	0
**Maximum**	T0	8	76	1310.000	25	104
	1m	8	69	2290.000	25	91
	3m	8	56	2150.000	25	58
	6m	8	62	2770.000	25	89
	9m	8	60	2040.000	25	74
	12m	7	67	3300.000	25	82
	18m	7	42	1300.000	25	60
	24m	7	27	2400.000	25	82
**Skewness**	T0	-1.869	-0.368	1.181	-0.650	0.007

	Time	NPS	VAS	Eosinophil	ACT	SNOT-22
	1m	-0.074	1.320	1.712	-0.567	0.561
	3m	0.235	0.940	0.864	-2.776	0.530
	6m	0.631	1.506	1.644	-1.313	1.155
	9m	0.901	1.141	1.052	-1.764	0.856
	12m	0.564	1.976	2.364	-2.764	1.255
	18m	1.065	1.212	0.353	-1.461	1.036
	24m	0.564	0.966	2.564	-2.099	1.255
**Std. error skewness**	T0	0.369	0.383	0.383	0.398	0.369
	1m	0.383	0.409	0.388	0.414	0.378
	3m	0.374	0.388	0.393	0.388	0.369
	6m	0.393	0.409	0.403	0.414	0.393
	9m	0.448	0.448	0.456	0.448	0.441
	12m	0.393	0.398	0.393	0.403	0.393
	18m	0.448	0.448	0.456	0.456	0.441
	24m	0.393	0.481	0.524	0.481	0.393
**Kurtosis**	T0	4.596	-0.375	1.300	-0.593	-1.027
	1m	-0.714	1.587	4.690	-1.192	-0.255
	3m	-1.396	1.280	0.547	10.227	-0.232
	6m	-0.327	2.293	3.597	0.881	1.987
	9m	0.339	0.447	0.528	3.416	-0.229
	12m	-0.991	4.895	7.491	9.810	1.885
	18m	0.026	1.381	-0.955	1.080	0.648
	24m	-0.991	0.166	8.255	4.253	1.885
**Std. error kurtosis**	T0	0.724	0.450	0.750	0.778	0.724
	1m	0.750	0.798	0.759	0.809	0.741
	3m	0.733	0.759	0.768	0.759	0.724
	6m	0.768	0.798	0.788	0.809	0.768
	9m	0.872	0.872	0.887	0.872	0.858
	12m	0.768	0.778	0.768	0.788	0.768
	18m	0.872	0.872	0.887	0.887	0.858
	24m	0.768	0.935	1.014	0.935	0.768
**Shapiro-Wilk (*W*)**	T0	0.731	0.897	0.929	0.920	0.946
	1m	0.955	0.867	0.866	0.869	0.946
	3m	0.895	0.942	0.935	0.692	0.965
	6m	0.921	0.858	0.859	0.810	0.923
	9m	0.876	0.869	0.898	0.761	0.900
	12m	0.880	0.799	0.779	0.664	0.901
	18m	0.791	0.892	0.944	0.729	0.905
	24m	0.880	0.892	0.737	0.689	0.901
**Shapiro-Wilk (*P* value)**	T0	< .001	0.274	0.018	0.015	0.050
	1m	0.131	< .001	< .001	0.001	0.061
	3m	0.001	0.055	0.036	< .001	0.232
	6m	0.013	< .001	< .001	< .001	0.015
	9m	0.004	0.003	0.014	< .001	0.012

	Time	NPS	VAS	Eosinophil	ACT	SNOT-22
	12m	0.001	< .001	< .001	< .001	0.004
	18m	< .001	0.009	0.172	< .001	0.015
	24m	0.001	0.017	< .001	< .001	0.004

Table S4 – Extended descriptive statistics for continuous variables at each time-point of Figure 2 (Mepolizumab)

	Time	NPS	VAS	Eosinophil	ACT	SNOT-22
**Mean**	T0	5.387	40.115	695.484	19.654	53.742
	1m	4.370	31.077	192.522	19.476	43.296
	3m	4.056	24.625	224.000	22.438	36.167
	6m	3.375	23.071	131.923	22.600	31.625
	9m	3.667	18.000	73.333	21.750	26.556
	12m	3.333	16.545	54.000	23.273	29.750
	18m	3.000	17.818	61.000	24.091	23.636
	24m	0.800	11.250	100.000	24.600	21.200
**Std. error mean**	T0	0.324	2.947	67.441	0.926	3.055
	1m	0.389	3.715	52.802	1.182	4.245
	3m	0.475	4.564	69.233	0.841	5.202
	6m	0.515	4.195	42.159	0.524	4.552
	9m	0.527	4.048	14.240	1.473	5.242
	12m	0.527	3.985	15.217	0.574	5.851
	18m	0.661	4.251	14.564	0.415	4.724
	24m	0.490	6.019	34.400	0.245	8.071
**95% CI mean lower bound**	T0	4.753	34.339	563.301	17.838	47.754
	1m	3.608	23.796	89.031	17.159	34.976
	3m	3.125	15.679	88.307	20.788	25.971
	6m	2.365	14.849	49.292	21.574	22.703
	9m	2.634	10.065	45.423	18.863	16.282
	12m	2.300	8.735	24.175	22.149	18.283
	18m	1.705	9.487	32.455	23.278	14.378
	24m	-0.160	-0.547	32.578	24.120	5.381
**95% CI mean upper bound**	T0	6.021	45.892	827.667	21.470	59.730
	1m	5.133	38.358	296.013	21.794	51.617
	3m	4.986	33.571	359.693	24.087	46.362
	6m	4.385	31.294	214.554	23.626	40.547
	9m	4.700	25.935	101.243	24.637	36.829
	12m	4.366	24.356	83.825	24.397	41.217
	18m	4.295	26.150	89.545	24.904	32.895
	24m	1.760	23.047	167.422	25.080	37.019
**Median**	T0	6	35.000	650	21.000	54
	1m	5	26.000	100	22	38
	3m	4.000	22.500	100	24.000	32.500
	6m	4.000	22.500	100	22	33.500
	9m	4	14	80	23.500	36
	12m	4.000	15	50.000	24	27.000
	18m	3	16	60.000	25	21
	24m	0	9.500	75.000	25	24

	Time	NPS	VAS	Eosinophil	ACT	SNOT-22
**Standard deviation**	T0	1.801	15.029	375.498	4.724	17.010
	1m	2.022	18.942	253.232	5.419	22.059
	3m	2.014	18.257	268.136	3.366	22.070
	6m	2.062	15.696	152.008	2.028	18.209
	9m	1.581	12.145	42.720	4.166	15.725
	12m	1.826	13.216	48.120	1.902	20.267
	18m	2.191	14.098	46.056	1.375	15.667
	24m	1.095	12.038	68.799	0.548	18.047
**IQR**	T0	1.000	19.750	425.000	7.000	21.000
	1m	2.500	23.500	120.000	9.000	28.500
	3m	2.500	10.000	195.000	3.250	29.000
	6m	2.500	14.500	50.000	4.000	31.250
	9m	2.000	15.000	60.000	5.250	26.000
	12m	2.000	17.000	95.000	3.000	29.750
	18m	2.000	10.500	80.000	1.000	20.500
	24m	2.000	16.250	60.000	1.000	23.000
**Range**	T0	8	54	1590	17	69
	1m	7	79	992	17	103
	3m	7	76	830	12	87
	6m	6	52	595	6	57
	9m	4	36	130	10	43
	12m	6	38	120	5	58
	18m	7	48	120	4	47
	24m	2	26	150	1	45
**Minimum**	T0	0	12	100	8	29
	1m	0	1	8	8	4
	3m	0	1	30	13	2
	6m	0	1	5	19	1
	9m	2	1	20	15	2
	12m	0	0	0	20	0
	18m	0	0	0	21	0
	24m	0	0	50	24	0
**Maximum**	T0	8	66	1690	25	98
	1m	7	80	1000	25	107
	3m	7	77	860	25	89
	6m	6	53	600	25	58
	9m	6	37	150	25	45
	12m	6	38	120	25	58
	18m	7	48	120	25	47
	24m	2	26	200	25	45
**Skewness**	T0	-1.285	0.169	0.877	-0.776	0.822
	1m	-1.002	0.719	2.618	-0.580	0.875
	3m	-0.766	1.658	1.781	-1.717	0.675

	Time	NPS	VAS	Eosinophil	ACT	SNOT-22
	6m	-0.214	0.596	2.759	-0.078	-0.067
	9m	0.461	0.336	0.311	-0.950	-0.487
	12m	-0.271	0.627	0.081	-0.476	-0.034
	18m	0.418	1.118	-0.208	-1.606	0.266
	24m	0.609	0.509	1.658	-0.609	0.127
**Std. error skewness**	T0	0.421	0.456	0.421	0.456	0.421
	1m	0.448	0.456	0.481	0.501	0.448
	3m	0.536	0.564	0.580	0.564	0.536
	6m	0.564	0.597	0.616	0.580	0.564
	9m	0.717	0.717	0.717	0.752	0.717
	12m	0.637	0.661	0.687	0.661	0.637
	18m	0.661	0.661	0.687	0.661	0.661
	24m	0.913	1.014	1.014	0.913	0.913
**Kurtosis**	T0	1.703	-0.949	0.583	0.045	0.964
	1m	0.359	0.516	6.429	-0.972	1.388
	3m	0.020	3.976	2.211	3.077	0.623
	6m	-0.991	-0.099	8.613	-1.175	-1.428
	9m	-1.029	-1.060	-0.302	-0.785	-1.588
	12m	-0.324	-0.881	-1.926	-1.449	-1.610
	18m	-0.156	0.994	-1.575	1.703	-0.986
	24m	-3.333	-2.629	2.690	-3.333	-1.288
**Std. error kurtosis**	T0	0.821	0.887	0.821	0.887	0.821
	1m	0.872	0.887	0.935	0.972	0.872
	3m	1.038	1.091	1.121	1.091	1.038
	6m	1.091	1.154	1.191	1.121	1.091
	9m	1.400	1.400	1.400	1.481	1.400
	12m	1.232	1.279	1.334	1.279	1.232
	18m	1.279	1.279	1.334	1.279	1.279
	24m	2.000	2.619	2.619	2.000	2.000
**Shapiro-Wilk (*W*)**	T0	0.845	0.953	0.937	0.915	0.936
	1m	0.873	0.951	0.605	0.874	0.945
	3m	0.913	0.849	0.712	0.780	0.961
	6m	0.904	0.940	0.650	0.891	0.932
	9m	0.858	0.946	0.941	0.785	0.880
	12m	0.926	0.908	0.859	0.819	0.916
	18m	0.940	0.902	0.892	0.714	0.939
	24m	0.684	0.923	0.823	0.684	0.965
**Shapiro-Wilk (*P* value)**	T0	< .001	0.279	0.067	0.034	0.063
	1m	0.003	0.247	< .001	0.011	0.163
	3m	0.096	0.013	< .001	0.001	0.612
	6m	0.095	0.417	< .001	0.070	0.260
	9m	0.092	0.648	0.593	0.020	0.156
	12m	0.339	0.228	0.074	0.017	0.258
	18m	0.522	0.197	0.177	< .001	0.504

	Time	NPS	VAS	Eosinophil	ACT	SNOT-22
	24m	0.006	0.554	0.149	0.006	0.843

Following the original numbering of the figures in the article, a new plot homologous to the original Figure 1 (box-violin plots at each time-point for Dupilumab group) has been produced after application of the propensity score for matching (see Section S5, pp. 29-45). Considering the matching type obtained by applying the propensity score, a new plot homologous to the original Figure 2 (box-violin plots at each time-point for Mepolizumab group) would be identical to the original, and obviously it was not re-proposed. The plot homologous to the original Figure 1 built on the matched sample, as well as the extended descriptive statistics, are presented on pages 69-73.

Section S4. Additional analysis performed on the original (unmatched) data

S4.1 – Eosinophil count

The eosinophil (EOS) count represents a critical parameter for asthmatic patients and related treatments. In the Mepolizumab group significatively higher EOS values than in Dupilumab group occurred ($P = 0.040$; Table 2 of the paper). However, in the Dupilumab group there were $n = 3$ missing values. Thus, these missing values were calculated within the Dupilumab group by entering the mean value of the available values in the same variable (mean imputation method). Thus, we have renamed the original "*Eosinophil*" variable, with $n = 3$ missing values in the Dupilumab group, as "*Eosinophil_or*", while the variable obtained after mean imputation method was named as "*Eosinophil_mi*". The descriptive statistics for these variables in all enrolled patients are presented in **Table S5**.

Table S5 – Extended descriptive statistics for the "*Eosinophil_or*" and "*Eosinophil_mi*" variables (all cases).

	Eosinophil_or	Eosinophil_mi
Mean	601.333	598.278
Std. error mean	40.098	38.455
95% CI mean lower bound	522.742	522.907
95% CI mean upper bound	679.925	673.649
Median	510	522.500
Standard deviation	333.082	326.304
IQR	420.000	390.000
Range	1590	1590
Minimum	100	100
Maximum	1690	1690
Skewness	1.118	1.165
Std. error skewness	0.289	0.283
Kurtosis	1.157	1.363
Std. error kurtosis	0.570	0.559
Shapiro-Wilk W	0.915	0.912
Shapiro-Wilk p	< .001	< .001

The histograms combined with their corresponding density plots are shown in **Figure S1**.

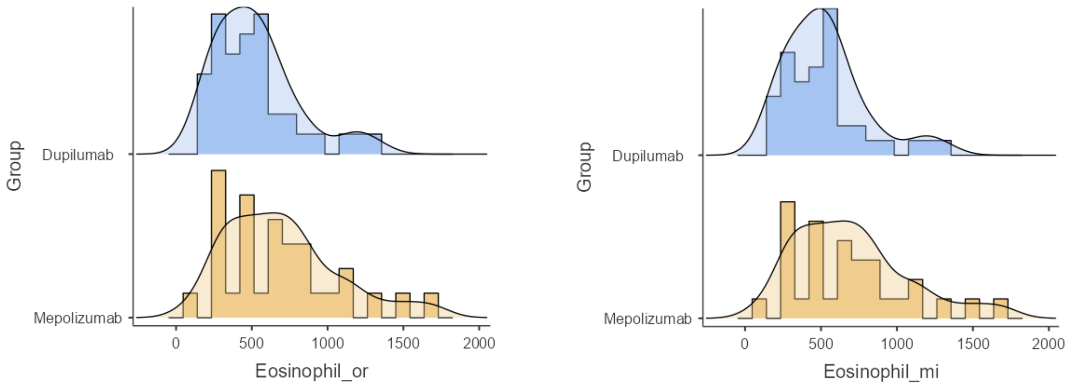

Figure S1 – Histogram with density plot for "*Eosinophil_or*" (original data, with n = 3 missing values in the Dupilumab group) and "*Eosinophil_mi*" (data after mean imputation for the missing cases in the Dupilumab group) variables, in both Dupilumab and Mepolizumab group.

The differences between comparisons with raw data and after mean imputation are reported in **Table S6**.

Table S6 – Mann-Whitney test performed by comparing Eosinophil count (cells/μL) in Dupilumab *vs.* Mepolizumab group ("*Eosinophil_or*": raw data; "*Eosinophil_mi*": data after mean imputation for n = 3 missing values in the Dupilumab group).

						95% Confidence Interval	
		Statistic	p	Mean difference	SE difference	Lower	Upper
Eosinophil_or	Mann-Whitney U	418.500	0.040	−140.000		−300.000	−6.465e−6
Eosinophil_mi	Mann-Whitney U	457.500	0.043	−140.000		−290.000	−6.057e−6

Group Descriptives

	Group	N	Mean	Median	SD	SE
Eosinophil_or	Dupilumab	38	524.526	500.000	275.840	44.747
	Mepolizumab	31	695.484	650.000	375.498	67.441
Eosinophil_mi	Dupilumab	41	524.780	500.000	265.299	41.433
	Mepolizumab	31	695.484	650.000	375.498	67.441

By assuming the eosinophil count > 600 cells/μL as cut-off value (ref. variable: "*Eosinophil.mi*"), we have created the "*Eosinophil_dic*" variable (encoded as "*Yes*": > 600 cells/μL; "*No*": ≤ 600 cells/μL). The main frequency statistics and the binomial test for this new variable are reported in **Table S7**.

Table S7 – Frequency statistics and binomial test for the "*Eosinophil_dic*" variable.

Frequencies of Eosinophil_dic

Levels	Counts	% of Total	Cumulative %
No	46	63.9 %	63.9 %
Yes	26	36.1 %	100.0 %

	Level	Count	Total	Proportion	p	95% Confidence Interval Lower	95% Confidence Interval Upper
Eosinophil_dic	No	46	72	0.639	0.024	0.517	0.749
	Yes	26	72	0.361	0.024	0.251	0.483

Note. H_a is proportion ≠ 0.5

The "*Eosinophil_dic*" has been used as stratifying variable for several comparisons (**Table S8, Table S9**).

Table S8 – Comparison between Dupilumab *vs*. Mepolizumab for "*Eosinophil_dic*" variable.

Contingency Tables

Eosinophil_dic		Group: Dupilumab	Group: Mepolizumab	Total
No	Observed	32	14	46
	% within row	69.6 %	30.4 %	100.0 %
	% within column	78.0 %	45.2 %	63.9 %
	% of total	44.4 %	19.4 %	63.9 %
Yes	Observed	9	17	26
	% within row	34.6 %	65.4 %	100.0 %
	% within column	22.0 %	54.8 %	36.1 %
	% of total	12.5 %	23.6 %	36.1 %
Total	Observed	41	31	72
	% within row	56.9 %	43.1 %	100.0 %
	% within column	100.0 %	100.0 %	100.0 %
	% of total	56.9 %	43.1 %	100.0 %

χ^2 Tests

	Value	df	p
χ^2	8.276	1	0.004
Fisher's exact test			0.006
N	72		

Table S9 – Comparison between the main continuous variables by stratifying for absence (No) / presence (Yes) of eosinophil count > 600 cells/μL.

		Statistic	p
Age	Mann-Whitney U	462.000	0.112
Cycles_N	Mann-Whitney U	565.000	0.695
NPS	Mann-Whitney U	503.000	0.223
SNOT.22	Mann-Whitney U	539.500	0.496
SSIT.16	Mann-Whitney U	503.000	0.263
IgE_Tot	Mann-Whitney U	383.500	0.602
ACT	Mann-Whitney U	389.500	0.377
VAS	Mann-Whitney U	416.500	0.445
Lund.Mackay	Mann-Whitney U	584.000	0.995
Access	Mann-Whitney U	557.500	0.746

Group Descriptives

	Group	N	Mean	Median	SD	SE
Age	No	46	59.196	61.000	12.631	1.862
	Yes	26	52.885	59.000	16.839	3.302
Cycles_N	No	46	5.087	1.000	8.398	1.238
	Yes	26	3.923	1.000	7.353	1.442
NPS	No	46	5.761	6.000	1.608	0.237
	Yes	26	5.346	6.000	1.765	0.346
SNOT.22	No	46	55.565	59.000	19.879	2.931
	Yes	26	59.692	57.000	18.203	3.570
SSIT.16	No	46	4.413	3.500	2.777	0.410
	Yes	26	4.769	5.000	1.904	0.373
IgE_Tot	No	38	370.397	143.500	656.025	106.421
	Yes	22	275.227	171.000	384.256	81.924
ACT	No	36	19.167	20.500	5.624	0.937
	Yes	25	18.440	20.000	4.700	0.940
VAS	No	41	41.610	45.000	16.378	2.558
	Yes	23	45.043	47.000	14.515	3.027
Lund.Mackay	No	45	18.622	20.000	4.811	0.717
	Yes	26	18.846	20.000	4.007	0.786
Access	No	45	10.889	7.000	7.886	1.176
	Yes	26	11.154	10.500	9.251	1.814

While the statistical significance returned by the contingency table was expected (Table S8), considering the criteria adopted for treatment assignment, no significant differences were found by stratifying the main continuous variables for the absence (No) / presence (Yes) of eosinophil count > 600 cells/μL (Table S9).

S4.2 – NSAID intolerance

Intolerance to nonsteroidal anti-inflammation drugs (NSAID) was evaluated in the paper (Table 1), finding a significant difference ($P = 0.029$) between Dupilumab (53.7%) *vs*. Mepolizumab (25.8%) group (**Table S10**).

Table S10 – Comparison between Dupilumab *vs*. Mepolizumab for NSAID intolerance.

Contingency Tables

NSAID_intol		Group: Dupilumab	Group: Mepolizumab	Total
No	Observed	19	23	42
	% within row	45.2 %	54.8 %	100.0 %
	% within column	46.3 %	74.2 %	58.3 %
	% of total	26.4 %	31.9 %	58.3 %
Yes	Observed	22	8	30
	% within row	73.3 %	26.7 %	100.0 %
	% within column	53.7 %	25.8 %	41.7 %
	% of total	30.6 %	11.1 %	41.7 %
Total	Observed	41	31	72
	% within row	56.9 %	43.1 %	100.0 %
	% within column	100.0 %	100.0 %	100.0 %
	% of total	56.9 %	43.1 %	100.0 %

χ^2 Tests

	Value	df	p
χ^2	5.634	1	0.018
Fisher's exact test			0.029
N	72		

Intolerance to NSAID had not been further stressed in the paper. Thus, in this section we used "*NSAID_intol*" variable for additional comparisons (**Table S11**).

Table S11 – Comparison between the main continuous variables by stratifying for absence (No) / presence (Yes) of NSAID intolerance.

		Statistic	p
Age	Mann-Whitney U	596.500	0.706
Cycles_N	Mann-Whitney U	578.000	0.545
NPS	Mann-Whitney U	540.000	0.260
SNOT.22	Mann-Whitney U	626.500	0.973
SSIT.16	Mann-Whitney U	564.500	0.453
IgE_Tot	Mann-Whitney U	319.500	0.078
Eosinophil_or	Mann-Whitney U	347.000	0.006
Eosinophil_mi	Mann-Whitney U	387.500	0.006
Access	Mann-Whitney U	578.500	0.673
Lund.Mackay	Mann-Whitney U	518.000	0.259
VAS	Mann-Whitney U	457.000	0.618
ACT	Mann-Whitney U	333.000	0.058

Group Descriptives

	Group	N	Mean	Median	SD	SE
Age	No	42	57.643	61.000	13.154	2.030
	Yes	30	55.900	59.000	16.382	2.991
Cycles_N	No	42	4.238	1.000	7.515	1.160
	Yes	30	5.267	1.000	8.737	1.595
NPS	No	42	5.405	6.000	1.768	0.273
	Yes	30	5.900	6.000	1.494	0.273
SNOT.22	No	42	56.905	58.500	18.287	2.822
	Yes	30	57.267	57.500	20.874	3.811
SSIT.16	No	42	4.786	4.500	2.628	0.405
	Yes	30	4.200	4.500	2.280	0.416
IgE_Tot	No	35	249.097	110.000	399.399	67.511
	Yes	25	456.468	201.000	738.799	147.760
Eosinophil_or	No	41	515.122	470.000	302.573	47.254
	Yes	28	727.571	615.000	340.545	64.357
Eosinophil_mi	No	42	515.357	485.000	298.865	46.116
	Yes	30	714.367	600.000	332.415	60.690
Access	No	41	11.146	7.000	8.156	1.274
	Yes	30	10.767	7.500	8.736	1.595
Lund.Mackay	No	41	18.146	19.000	4.973	0.777
	Yes	30	19.467	20.000	3.721	0.679
VAS	No	38	42.289	45.000	16.084	2.609
	Yes	26	43.654	48.500	15.412	3.022
ACT	No	32	20.000	21.000	5.010	0.886
	Yes	29	17.621	19.000	5.281	0.981

In Table S11, by stratifying for absence (No) / presence (Yes) of NSAID intolerance a statistical significance was found only in the eosinophil count-related variables (*P* = 0.006 for both "*Eosinophil_or*" and "*Eosinophil_mi*"). A contingency table between NSAID intolerance and asthma has been also evaluated. As expected, significant *P* values were found in both χ^2 and Fisher's exact test (**Table S12**).

Table S12 – Comparison between absence/presence of NSAID intolerance and absence/presence of asthma.

		NSAID_intol		
Asthma		No	Yes	Total
No	Observed	13	1	14
	% within row	92.9 %	7.1 %	100.0 %
	% within column	31.0 %	3.3 %	19.4 %
	% of total	18.1 %	1.4 %	19.4 %
Yes	Observed	29	29	58
	% within row	50.0 %	50.0 %	100.0 %
	% within column	69.0 %	96.7 %	80.6 %
	% of total	40.3 %	40.3 %	80.6 %
Total	Observed	42	30	72
	% within row	58.3 %	41.7 %	100.0 %
	% within column	100.0 %	100.0 %	100.0 %
	% of total	58.3 %	41.7 %	100.0 %

χ^2 Tests

	Value	df	p
χ^2	8.522	1	0.004
Fisher's exact test			0.005
N	72		

S4.3 – Asthma Control Test

The asthma control test (ACT) was treated in the paper as a continuous variable, since this test returns a score ranged from 5 to 25. On the other hand, by using a simple subgrouping criterium (score in the range 20-25: asthma well controlled; 16-19: partly controlled; 5-15: poorly controlled), the numerical "*ACT*" variable may be transformed into a three-layer categorization variable ("*Asthma_control*"; **Table S13**).

Table S13 – Frequency statistics for the "*Asthma_control*" variable.

Frequencies of Asthma_control

Levels	Counts	% of Total	Cumulative %
Good	32	52.5 %	52.5 %
Partly	14	23.0 %	75.4 %
Poorly	15	24.6 %	100.0 %

In our study, the ACT score was not available in all patients. Thus, the results for this parameter must be assumed with caution. By stratifying in a 2x3 table, we found no significant differences between treatment groups (**Table S14**), although a relatively better asthma control seems to occur in the Mepolizumab group.

Table S14 – Comparison between Dupilumab *vs*. Mepolizumab for quality of asthma control.

Contingency Tables

		Group		
Asthma_control		Dupilumab	Mepolizumab	Total
Good	Observed	18	14	32
	% within row	56.3 %	43.8 %	100.0 %
	% within column	51.4 %	53.8 %	52.5 %
	% of total	29.5 %	23.0 %	52.5 %
Partly	Observed	6	8	14
	% within row	42.9 %	57.1 %	100.0 %
	% within column	17.1 %	30.8 %	23.0 %
	% of total	9.8 %	13.1 %	23.0 %
Poorly	Observed	11	4	15
	% within row	73.3 %	26.7 %	100.0 %
	% within column	31.4 %	15.4 %	24.6 %
	% of total	18.0 %	6.6 %	24.6 %
Total	Observed	35	26	61
	% within row	57.4 %	42.6 %	100.0 %
	% within column	100.0 %	100.0 %	100.0 %
	% of total	57.4 %	42.6 %	100.0 %

χ^2 Tests

	Value	df	p
χ^2	2.785	2	0.248
Fisher's exact test			0.263
N	61		

In the next 2x3 table is reported the comparisons between asthma control and complications (**Table S15**).

Table S15 – Comparison between absence/presence of complications and quality of asthma control.

Contingency Tables

		Complications		
Asthma_control		No	Yes	Total
Good	Observed	29	3	32
	% within row	90.6 %	9.4 %	100.0 %
	% within column	58.0 %	27.3 %	52.5 %
	% of total	47.5 %	4.9 %	52.5 %
Partly	Observed	12	2	14
	% within row	85.7 %	14.3 %	100.0 %
	% within column	24.0 %	18.2 %	23.0 %
	% of total	19.7 %	3.3 %	23.0 %
Poorly	Observed	9	6	15
	% within row	60.0 %	40.0 %	100.0 %
	% within column	18.0 %	54.5 %	24.6 %
	% of total	14.8 %	9.8 %	24.6 %
Total	Observed	50	11	61
	% within row	82.0 %	18.0 %	100.0 %
	% within column	100.0 %	100.0 %	100.0 %
	% of total	82.0 %	18.0 %	100.0 %

χ^2 Tests

	Value	df	p
χ^2	6.653	2	0.036
Fisher's exact test			0.042
N	61		

In the general one-way non-parametric ANOVA model (Kruskal-Wallis method) by stratifying for the "*Asthma_control*" variable, a statistical significance was found only for "*SNOT-22*", "*Lund-Mackay*" and "*Access*" variables (**Table S16**). For these variables, the *post-hoc* pairwise comparisons (Dwass-Steel-Critchlow-Fligner method) are reported in **Table S17**.

Table S16 – General one-way non-parametric ANOVA, using "*Asthma control*" as grouping variable.

	χ^2	df	p	ε^2
Age	4.303	2	0.116	0.072
Cycles_N	4.482	2	0.106	0.075
NPS	3.257	2	0.196	0.054
SNOT.22	11.902	2	0.003	0.198
SSIT.16	0.180	2	0.914	0.003
IgE_Tot	0.555	2	0.758	0.012
Eosinophil_or	2.640	2	0.267	0.046
Eosinophil_mi	2.348	2	0.309	0.039
VAS	4.176	2	0.124	0.079
Lund.Mackay	8.366	2	0.015	0.139
Access	6.073	2	0.048	0.101

ε^2: effect size.

Descriptives

	Asthma_control	Age	Cycles_N	NPS	SNOT.22	SSIT.16	IgE_Tot	Eosinophil_or	Eosinophil_mi	VAS	Lund.Mackay	Access
N	Good	32	32	32	32	32	25	31	32	29	32	32
	Partly	14	14	14	14	14	11	13	14	13	14	14
	Poorly	15	15	15	15	15	13	14	15	12	15	15
Mean	Good	58.438	2.531	5.563	50.156	4.406	306.092	550.710	550.188	40.414	17.031	13.656
	Partly	49.500	6.286	4.857	57.929	4.643	336.273	692.308	680.357	43.923	19.429	9.071
	Poorly	59.533	9.000	6.067	72.867	4.467	456.669	750.000	735.000	51.833	21.200	8.133
95% CI mean lower bound	Good	53.319	0.521	4.953	44.200	3.480	84.441	449.772	452.449	34.580	15.143	10.667
	Partly	42.228	0.907	3.833	48.739	3.111	107.350	450.126	454.920	36.778	17.713	4.531
	Poorly	52.359	3.769	5.365	62.640	3.681	66.353	564.209	559.557	43.902	20.098	4.309
95% CI mean upper bound	Good	63.556	4.542	6.172	56.113	5.333	527.743	651.648	647.926	46.247	18.920	16.645
	Partly	56.772	11.665	5.882	67.118	6.175	565.196	934.489	905.794	51.068	21.144	13.612
	Poorly	66.708	14.231	6.769	83.094	5.252	846.986	935.791	910.443	59.765	22.302	11.958
Median	Good	61.000	1.000	6.000	48.000	4.000	181.000	520	527.000	42	17.000	14.000
	Partly	47.000	1.000	6.000	57.000	4.500	242.000	500	512.500	47	20.500	5.000
	Poorly	60	3	6	80	5	209.000	665.000	600	51.500	22	6
Standard deviation	Good	14.773	5.803	1.759	17.192	2.674	565.448	286.740	282.092	16.028	5.451	8.627
	Partly	13.883	10.269	1.956	17.543	2.925	387.381	445.518	430.369	13.143	3.275	8.669
	Poorly	14.177	10.337	1.387	20.209	1.552	718.027	354.683	346.683	14.018	2.178	7.558
IQR	Good	20.750	2.000	0.250	27.750	3.250	135.000	395.000	392.500	25.000	8.250	18.000
	Partly	19.000	4.750	2.750	19.250	4.500	285.500	720.000	622.500	21.000	4.750	13.750
	Poorly	10.500	19.500	0.500	23.500	2.000	192.000	527.500	485.000	11.250	2.500	10.000
Minimum	Good	23	0	0	26	0	24.000	100	100	9	2	0
	Partly	23	0	0	32	0	44.000	200	200	18	14	0
	Poorly	30	0	2	30	2	14.700	300	300	26	17	0
Maximum	Good	86	24	8	92	10	2886.000	1500	1500	69	28	24
	Partly	71	30	7	96	10	1269.000	1690	1690	64	24	24
	Poorly	81	25	8	104	8	2287.000	1310	1310	76	24	24

Table S17 – The *post-hoc* pairwise comparisons for significant variables at the general one-way non-parametric ANOVA model for "*Asthma control*".

Pairwise comparisons - SNOT.22

		W	p
Good	Partly	1.791	0.414
Good	Poorly	4.682	0.003
Partly	Poorly	3.057	0.078

Pairwise comparisons - Lund.Mackay

		W	p
Good	Partly	2.002	0.333
Good	Poorly	3.910	0.016
Partly	Poorly	1.974	0.343

Pairwise comparisons - Access

		W	p
Good	Partly	−2.480	0.186
Good	Poorly	−3.111	0.071
Partly	Poorly	−0.341	0.969

S4.4 – EPOS

The *European Position paper on Rhinosinusitis and Nasal Polyps* (EPOS) is a long-standing initiative of the European Rhinologic Society for creating guidance in the management of patients affected by chronic rhinosinusitis with nasal polyps. In our study, we have included an EPOS follow-up at 6 and 12 months ("*EPOS_SIX*" and "*EPOS_TWELVE*" variables, respectively). These variables were encoded in this analysis as "*Good*", "*Moderate*" and "*Poorly*" regarding overall response to treatments (reduced nasal polyp size, reduced need for systemic corticosteroids, improved quality of life, improved sense of smell, reduced impact of co-morbidities). The "*Good*", "*Moderate*" and "*Poorly*" codes were used in this report for editorial reasons about R outputs; these codes correspond to the "*Good-Excellent responder*", "*Moderate responder*" and "*No-Poor responder*" used in the paper, respectively. For more details, see the "*Efficacy of Treatment*" section in the paper. The frequencies for patients who reached the follow-up time-points, as well as a comparison for treatments, are shown in **Table S18, Table S19** and **Table S20**, respectively. The low number of patients encoded as "*Moderate*" or "*Poorly*" does not allow to obtain reliable estimates for comparisons on continuous variables by using "*EPOS_SIX*" or "*EPOS_TWELVE*" as grouping variable.

Table S18 – Frequency statistics for the "*EPOS_SIX*" and "*EPOS_TWELVE*" variables.

Frequencies of EPOS_SIX

Levels	Counts	% of Total	Cumulative %
Good	49	87.5 %	87.5 %
Moderate	5	8.9 %	96.4 %
Poor	2	3.6 %	100.0 %

Frequencies of EPOS_TWELVE

Levels	Counts	% of Total	Cumulative %
Good	45	91.8 %	91.8 %
Moderate	3	6.1 %	98.0 %
Poor	1	2.0 %	100.0 %

Table S19 – Comparison between Dupilumab *vs*. Mepolizumab for "*EPOS_SIX*" variable.

Contingency Tables

EPOS_SIX		Group: Dupilumab	Group: Mepolizumab	Total
Good	Observed	36	13	49
	% within row	73.5 %	26.5 %	100.0 %
	% within column	90.0 %	81.3 %	87.5 %
	% of total	64.3 %	23.2 %	87.5 %
Moderate	Observed	3	2	5
	% within row	60.0 %	40.0 %	100.0 %
	% within column	7.5 %	12.5 %	8.9 %
	% of total	5.4 %	3.6 %	8.9 %
Poor	Observed	1	1	2
	% within row	50.0 %	50.0 %	100.0 %
	% within column	2.5 %	6.3 %	3.6 %
	% of total	1.8 %	1.8 %	3.6 %
Total	Observed	40	16	56
	% within row	71.4 %	28.6 %	100.0 %
	% within column	100.0 %	100.0 %	100.0 %
	% of total	71.4 %	28.6 %	100.0 %

χ^2 Tests

	Value	df	p
χ^2	0.870	2	0.647
Fisher's exact test			0.487
N	56		

Table S20 – Comparison between Dupilumab *vs*. Mepolizumab for "*EPOS_TWELVE*" variable.

Contingency Tables

		Group		
EPOS_TWELVE		Dupilumab	Mepolizumab	Total
Good	Observed	34	11	45
	% within row	75.6 %	24.4 %	100.0 %
	% within column	91.9 %	91.7 %	91.8 %
	% of total	69.4 %	22.4 %	91.8 %
Moderate	Observed	2	1	3
	% within row	66.7 %	33.3 %	100.0 %
	% within column	5.4 %	8.3 %	6.1 %
	% of total	4.1 %	2.0 %	6.1 %
Poor	Observed	1	0	1
	% within row	100.0 %	0.0 %	100.0 %
	% within column	2.7 %	0.0 %	2.0 %
	% of total	2.0 %	0.0 %	2.0 %
Total	Observed	37	12	49
	% within row	75.5 %	24.5 %	100.0 %
	% within column	100.0 %	100.0 %	100.0 %
	% of total	75.5 %	24.5 %	100.0 %

χ^2 Tests

	Value	df	p
χ^2	0.451	2	0.798
Fisher's exact test			1.000
N	49		

Section S5. Propensity Score Matching

Propensity Score Matching (PSM) may be used to reduce selection bias in observational studies, to balancing the observed covariates between groups. A PS is estimated using regression model methods (e.g., logistic or probit) conditioned on the observed baseline covariates. The most common PS target estimands are the "*average effect of the treatment on the treated*" (ATT), which is the effect for patients in the treatment group, and the "*average treatment effect*" (ATE), which is the effect on all individuals (treatment and control). The ATE is of more interest if every treatment potentially might be offered to every subject, whereas the ATT is preferable when patient's characteristics are more likely to determine the treatment received[1]. The covariate adjustment can estimate only marginal effect but neither the ATT nor the ATE[2].

In our retrospective study the PS approach seems to have several limitations, considering the different size of each native group, the low overall sample size and potential clinical overlapping of same variables, as well as the lack of a classical treatment-control design. Indeed, treatment assignment to Dupilumab or Mepolizumab was based on multiple clinical assessments and did not follow strictly random assignment criteria, although patients shared the same eligibility criteria. Especially, in view of the potential Dupilumab-induced hypereosinophilia[3-4], patients with a medium-high eosinophil count at the baseline or a hypereosinophilia history were preferably assigned to Mepolizumab group. For these reasons, in our study a perfect balance between the different covariates was difficult to achieve, hypothetically also discharging many cases from the original sample. Moreover, the initially enrolled sample already consisted of a relatively low number of patients, and a massive case-reduction strategy could return a too small subsample.

In any case, a PSM analysis was carried out by entering nine variables for weights and assuming Dupilumab and Mepolizumab as control and experimental treatment, respectively. The covariates were selected considering several factors (age, NSAID intolerance, previous surgery, allergy, asthma, smoke, eosinophil count > 600 cells/μL, NPS and SNOT-22 at the baseline). For the eosinophil count, $n = 3$ missing values in the Dupilumab group were filled with the mean of the available values in the same variable (mean imputation method)[5]. Considering that eosinophil count at the baseline was a critical factor for patient assignment to Mepolizumab group, the ATT was applied as target estimand.

By using the `matchit` function of the "*MatchIt*" R package in a first model with ATT (Average Treatment Effect on the Treated) as target estimand, the PS matching was obtained with the Greedy method (nearest neighbor approach) without replacement (1:1 matching).

```
# A `matchit` object
 - method: 1:1 nearest neighbor matching without replacement
 - distance: Propensity score
             - estimated with logistic regression
 - number of obs.: 72 (original), 62 (matched)
 - target estimand: ATT
 - method: nearest
 - covariates: Age, NSAID_intol, Previous_Surg, Eosinophil_dic, Allergy, Asthma, Smoke, NPS, SNOT.22
```

---

1 Fokkens W.J. et al. *EPOS/EUFOREA update on indication and evaluation of Biologics in Chronic Rhinosinusitis with Nasal Polyps 2023*. Rhinology. 2023;61(3):194-202.

2 *Ibidem.*

3 Li H.S. et al. *Eosinophilia and adverse effects of Dupilumab for respiratory indications: A real-world setting.* J Allergy Clin Immunol Pract. 2025;13(1):121-131.

4 Caminati M. et al. *Dupilumab-induced hypereosinophilia: review of the literature and algorithm proposal for clinical management.* Expert Rev Respir Med.2022;16(7):713-721.

5 For more details, see the previous Section S4.1.

```
# Summary of Balance for All Data:
                  Means Treated Means Control Std. Mean Diff. Var. Ratio eCDF Mean eCDF Max
distance                 0.6393        0.2727          1.3171     1.6815    0.3466   0.5940
Age                     57.0968       56.7805          0.0205     1.2273    0.0371   0.0865
NSAID_intolNo            0.7419        0.4634          0.6365          .    0.2785   0.2785
NSAID_intolYes           0.2581        0.5366         -0.6365          .    0.2785   0.2785
Previous_SurgNo          0.3548        0.1463          0.4358          .    0.2085   0.2085
Previous_SurgYes         0.6452        0.8537         -0.4358          .    0.2085   0.2085
Eosinophil_dicNo         0.4516        0.7805         -0.6609          .    0.3289   0.3289
Eosinophil_dicYes        0.5484        0.2195          0.6609          .    0.3289   0.3289
AllergyNo                0.3548        0.3171          0.0789          .    0.0378   0.0378
AllergyYes               0.6452        0.6829         -0.0789          .    0.0378   0.0378
AsthmaNo                 0.1935        0.1951         -0.0040          .    0.0016   0.0016
AsthmaYes                0.8065        0.8049          0.0040          .    0.0016   0.0016
SmokeNo                  0.7742        0.9024         -0.3067          .    0.1282   0.1282
SmokeYes                 0.2258        0.0976          0.3067          .    0.1282   0.1282
NPS                      5.3871        5.7805         -0.2184     1.3379    0.0482   0.1518
SNOT.22                 53.7419       59.5610         -0.3421     0.6779    0.1083   0.2777

# Summary of Balance for Matched Data:
                  Means Treated Means Control Std. Mean Diff. Var. Ratio eCDF Mean eCDF Max Std. Pair Dist.
distance                 0.6393        0.3433          1.0634     1.9422    0.2603   0.4839          1.0634
Age                     57.0968       57.0000          0.0063     1.3328    0.0492   0.0968          1.0674
NSAID_intolNo            0.7419        0.5484          0.4423          .    0.1935   0.1935          1.0321
NSAID_intolYes           0.2581        0.4516         -0.4423          .    0.1935   0.1935          1.0321
Previous_SurgNo          0.3548        0.1935          0.3371          .    0.1613   0.1613          0.8765
Previous_SurgYes         0.6452        0.8065         -0.3371          .    0.1613   0.1613          0.8765
Eosinophil_dicNo         0.4516        0.7097         -0.5186          .    0.2581   0.2581          0.9075
Eosinophil_dicYes        0.5484        0.2903          0.5186          .    0.2581   0.2581          0.9075
AllergyNo                0.3548        0.3548          0.0000          .    0.0000   0.0000          0.5161
AllergyYes               0.6452        0.6452          0.0000          .    0.0000   0.0000          0.5161
AsthmaNo                 0.1935        0.1935          0.0000          .    0.0000   0.0000          0.3226
AsthmaYes                0.8065        0.8065          0.0000          .    0.0000   0.0000          0.3226
SmokeNo                  0.7742        0.8710         -0.2315          .    0.0968   0.0968          0.6944
SmokeYes                 0.2258        0.1290          0.2315          .    0.0968   0.0968          0.6944
NPS                      5.3871        5.6774         -0.1612     1.0272    0.0363   0.0968          0.9491
SNOT.22                 53.7419       58.0645         -0.2541     0.7282    0.0981   0.2581          1.1910

# Sample Sizes:
          Control Treated
All            41      31
Matched        31      31
Unmatched      10       0
Discarded       0       0
```

The `bal.tab` function from the "*cobalt*" package was used to assess covariate balance before and after weighting. The balance table summarizes statistics such as standardized mean differences (SMD), variance ratios, and other metrics for each covariate.

```
# Balance Measures
                    Type Diff.Un      M.Threshold.Un
Age               Contin.  0.0205     Balanced, <0.05
NSAID_intol_Yes    Binary -0.2785 Not Balanced, >0.05
Previous_Surg_Yes  Binary -0.2085 Not Balanced, >0.05
Eosinophil_dic_Yes Binary  0.3289 Not Balanced, >0.05
Allergy_Yes        Binary -0.0378     Balanced, <0.05
Asthma_Yes         Binary  0.0016     Balanced, <0.05
Smoke_Yes          Binary  0.1282 Not Balanced, >0.05
NPS               Contin. -0.2184 Not Balanced, >0.05
SNOT.22           Contin. -0.3421 Not Balanced, >0.05

Balance tally for mean differences
                    count
Balanced, <0.05         3
Not Balanced, >0.05     6

Variable with the greatest mean difference
 Variable Diff.Un      M.Threshold.Un
  SNOT.22 -0.3421 Not Balanced, >0.05

Sample sizes
    Control Treated
All      41      31

Sample sizes
    Control Treated
All      41      31
```

The PS model shows that six variables are non-balanced, and that after matching $n = 10$ cases from the control group (Dupilumab) were unmatched. A visual inspection with absolute standardized mean differences (ASMD) and Kolmogorov-Smirnov (KS) statistics is shown in **Figure S2**. Notably, also after matching the not balanced variables remain outside from the threshold areas, conventionally set at 0.1 and 0.05 for ASMD and KS, respectively, although a marginal improved balance was obtained for all covariates. However, the balanced "*Age*" variable remains outside from the threshold area in the KS plot.

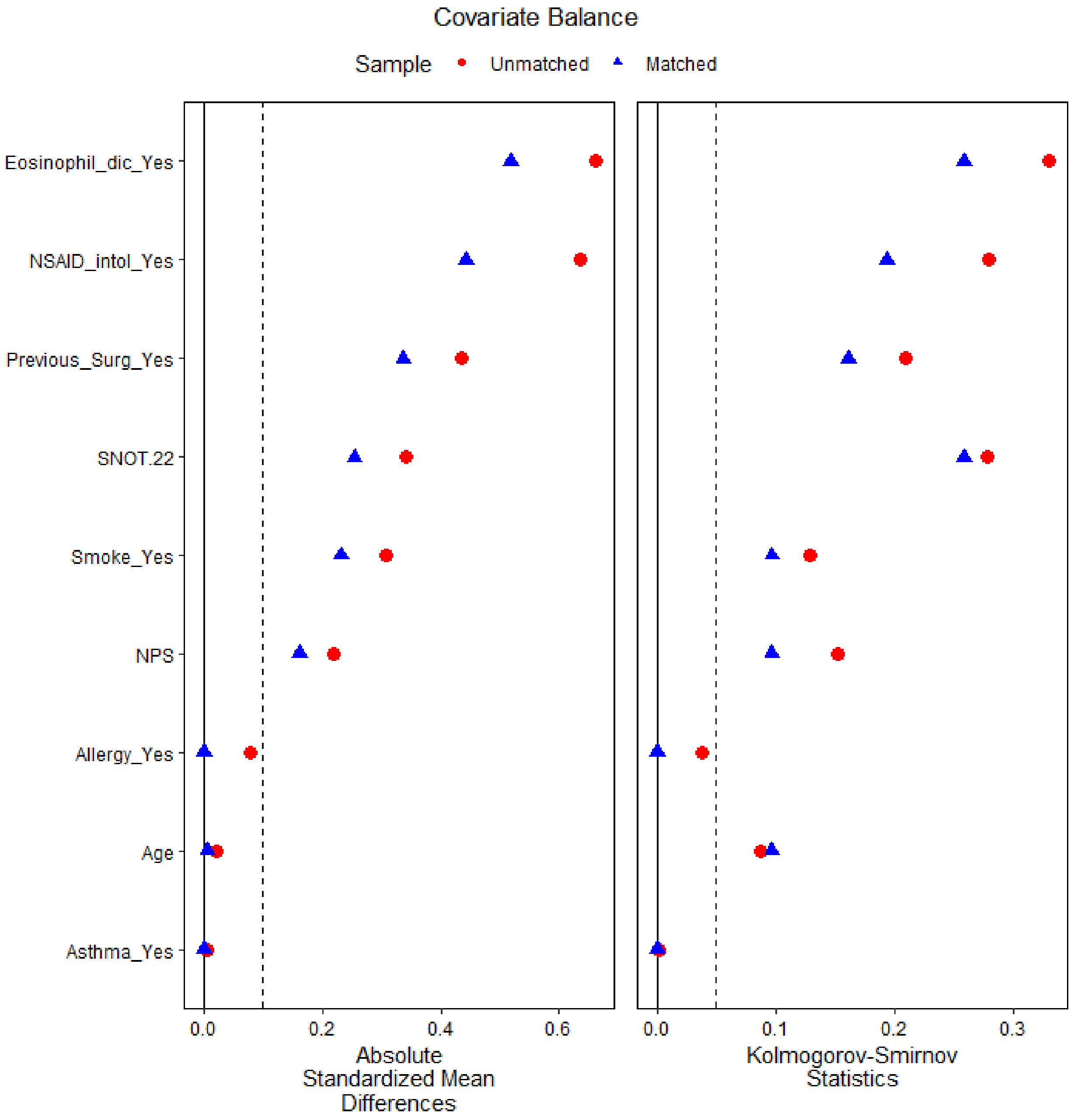

Figure S2 – Covariate balance for the multivariate PS model (nearest neighbor approach) without replacement (1:1 matching).

Empirical Quantile-Quantile (eQQ) plots were used to visualize covariate balance by comparing the distributions of Dupilumab and Mepolizumab groups before and after matching. Points along the 45-degree line indicate good balance, while deviations show imbalances (**Figure S3**, **Figure S4**, **Figure S5**, **Figure S6**, **Figure S7**, **Figure S8**).

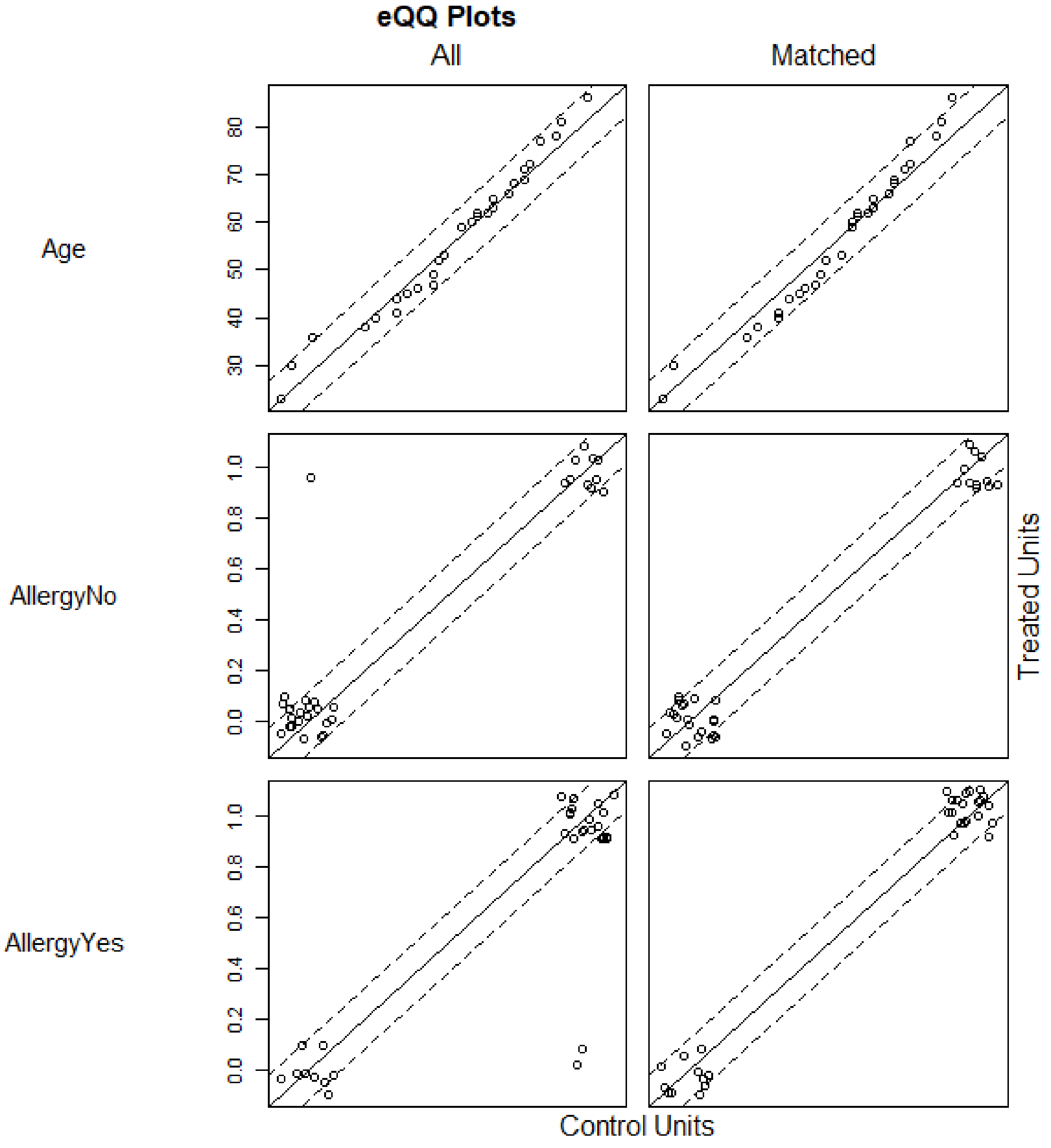

Figure S3 – Empirical Quantile-Quantile (eQQ) plots for "*Age*" and "*Allergy*" variables before and after matching.

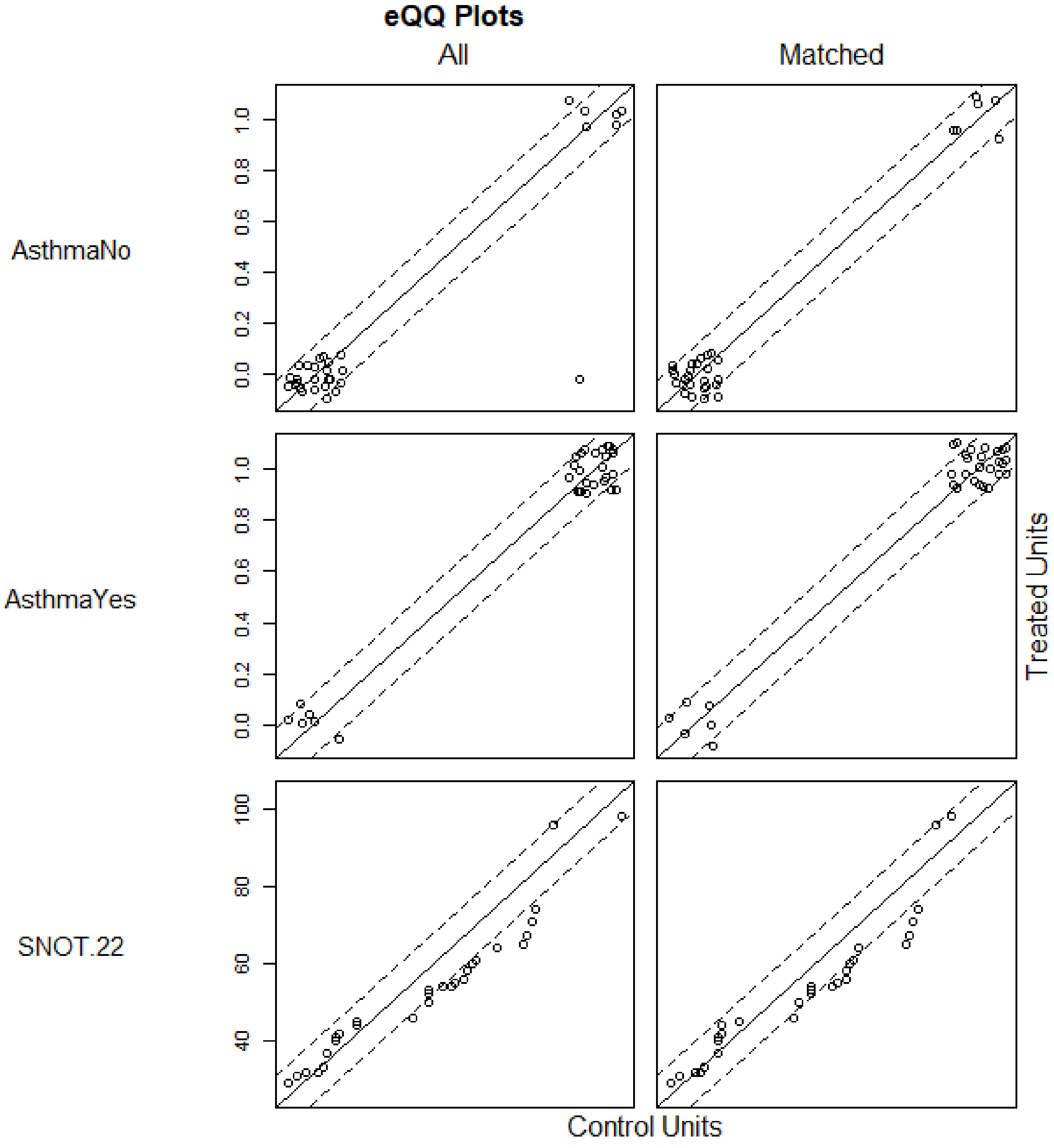

Figure S4 – Empirical Quantile-Quantile (eQQ) plots for "*Asthma*" and "*SNOT.22*" variables before and after matching.

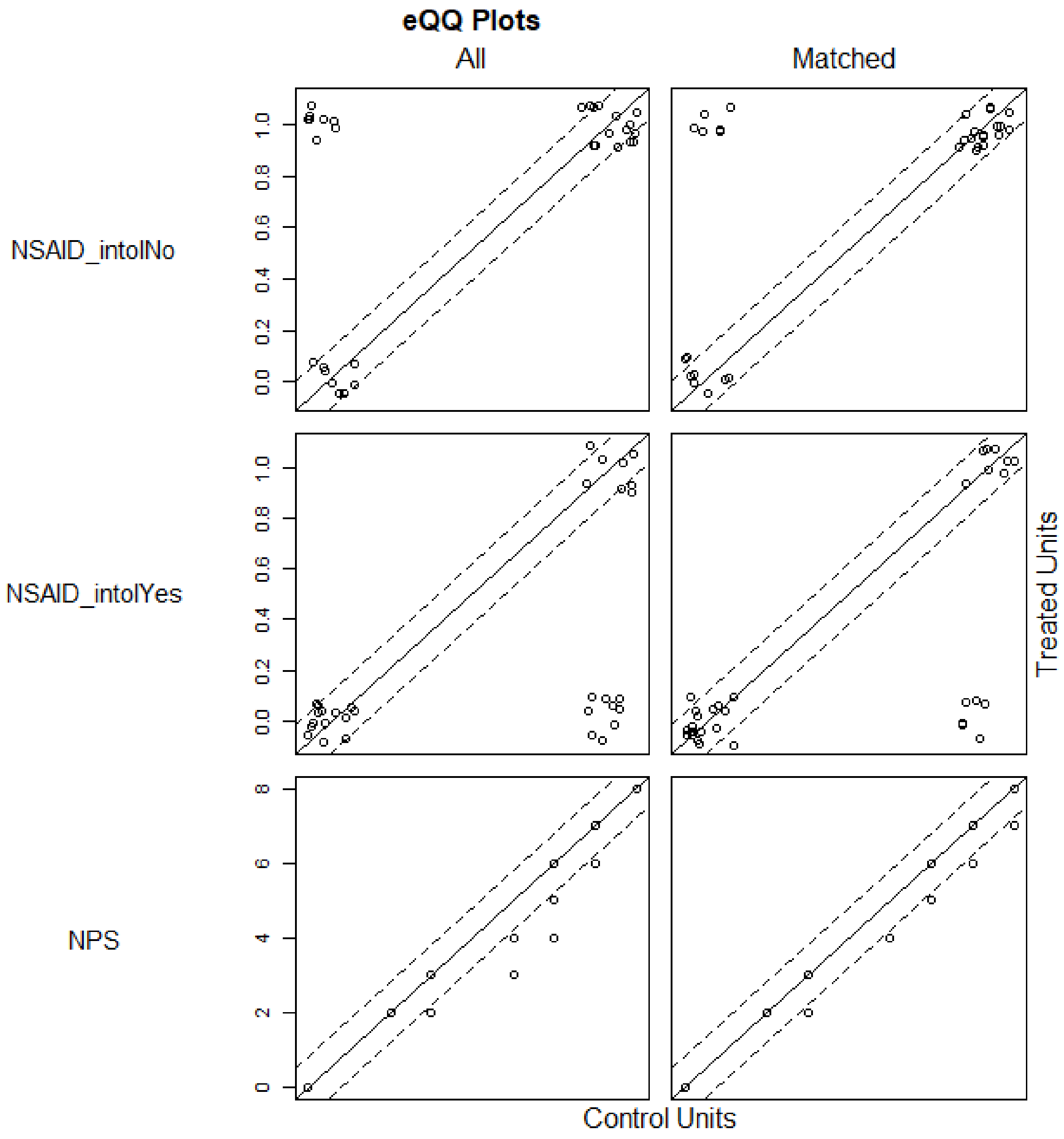

Figure S5 – Empirical Quantile-Quantile (eQQ) plots for "*NSAID_intol*" and "*NPS*" variables before and after matching.

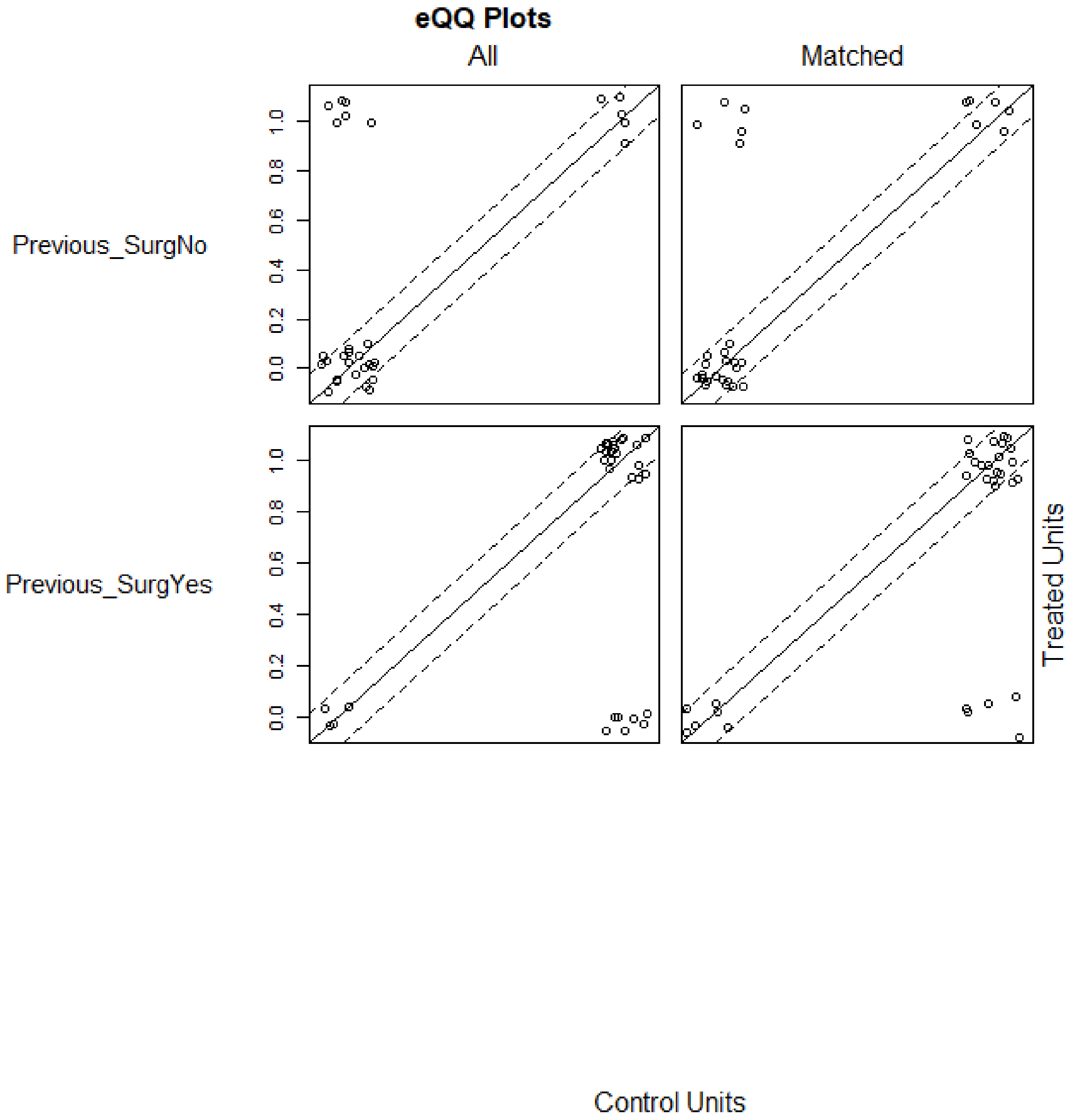

Figure S6 – Empirical Quantile-Quantile (eQQ) plots for "*Previous_Surg*" variable before and after matching.

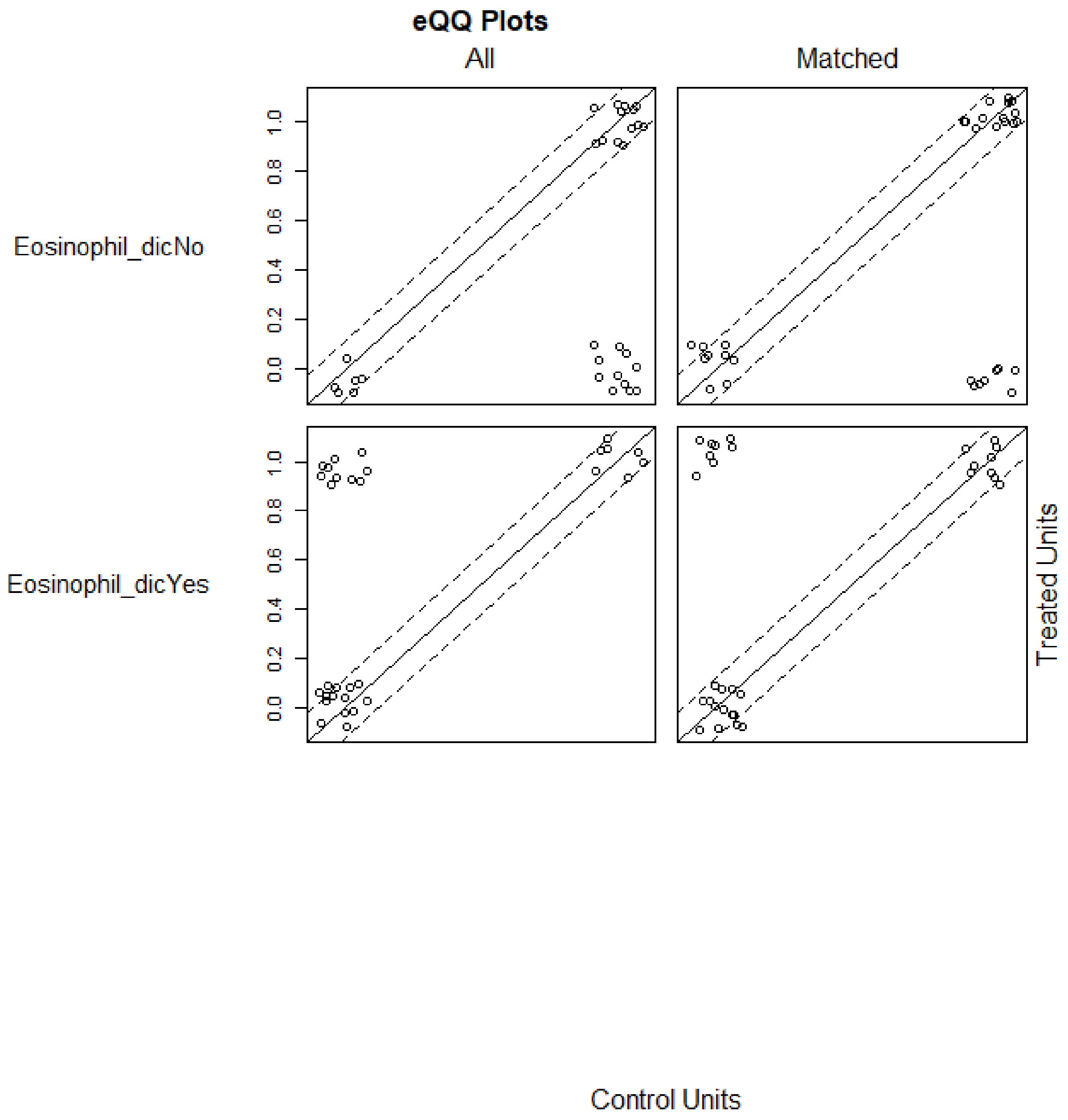

Figure S7 – Empirical Quantile-Quantile (eQQ) plots for "*Eosinophil_dic*" variable before and after matching.

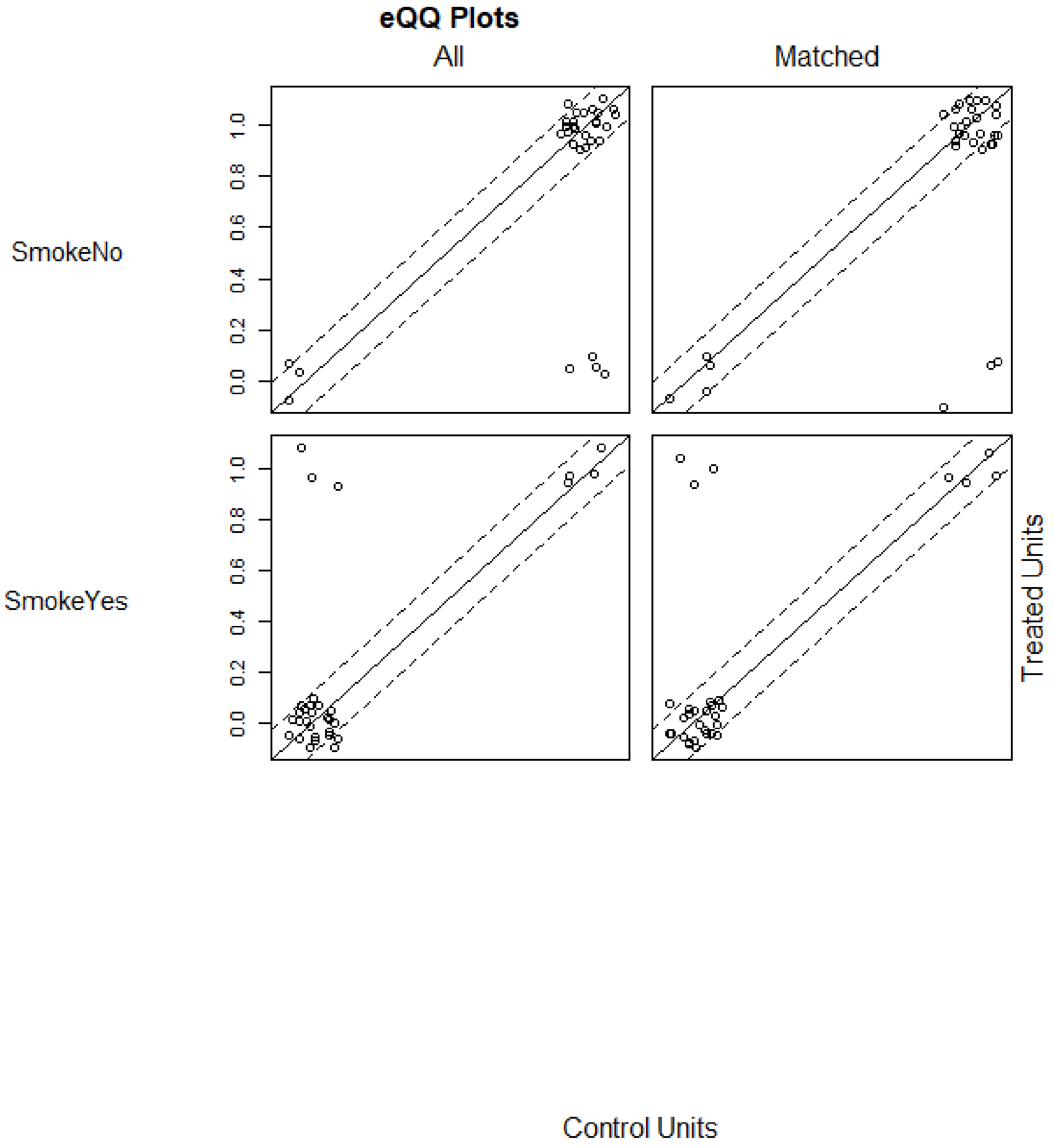

Figure S8 – Empirical Quantile-Quantile (eQQ) plots for "*Smoke*" variable before and after matching.

The eQQ plots showed a marginal improvement after matching, especially for outliers. In some unbalanced variables, after matching all or part of the outliers were placed near to the 45-degree line area (e.g., "*NPS*", "*Previous_Surg*", "*Smoke*"), as well as in the balanced variables ("*Allergy*" and "*Asthma*"). For the continuous covariates, density plots before and after matching are shown in **Figure S9**.

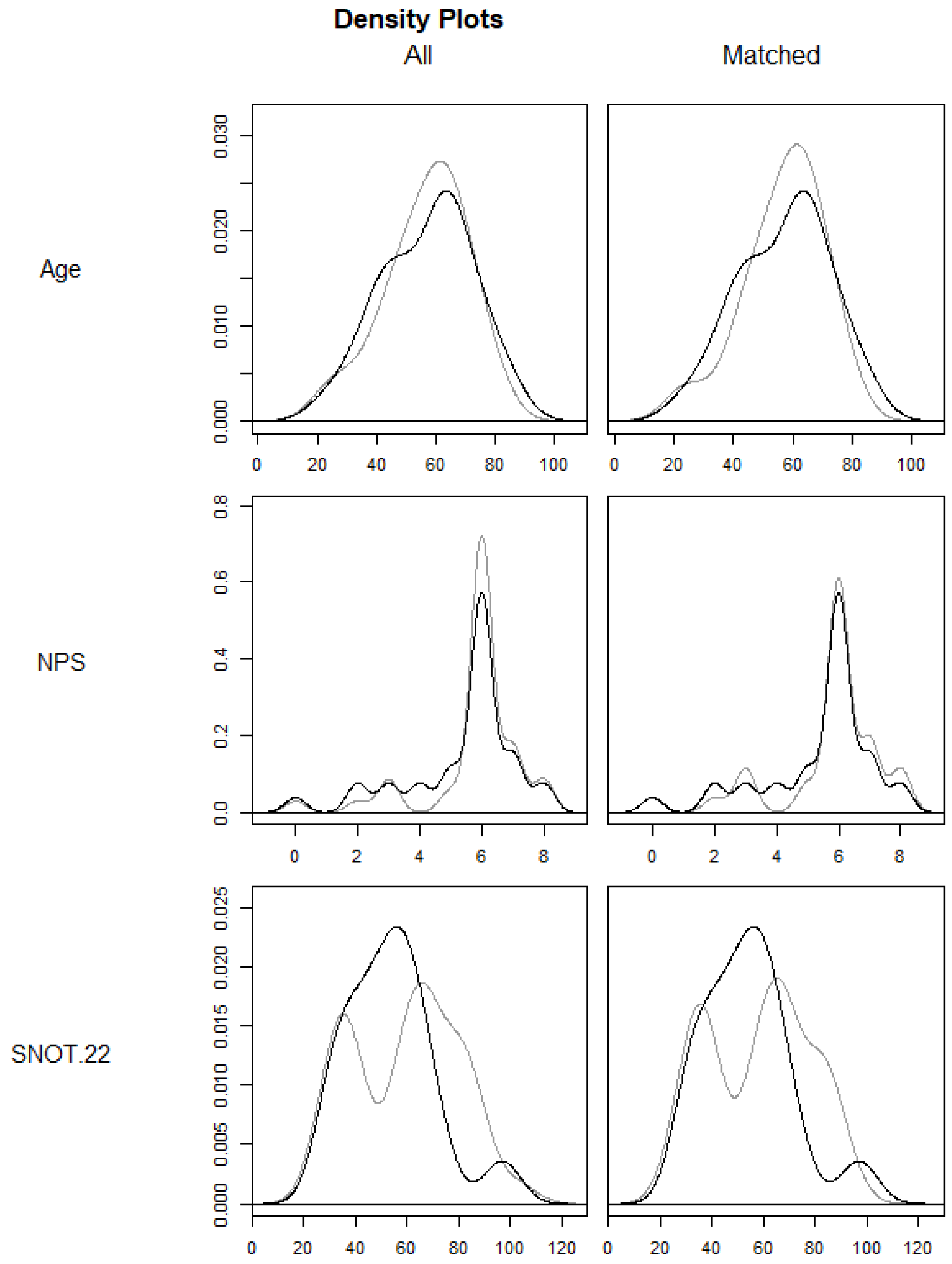

Figure S9 – Density plots for each continuous covariate entered in the PS model. The *x*-axis displays the covariate values, and the *y*-axis displays the density of the sample at that covariate value. The black line corresponds to the Mepolizumab group and the gray line to the Dupilumab group.

By using the **subclassification approach**, balance can be checked both within each subclass and overall. Thus, we have performed a propensity score subclassification using $n = 4$ subclasses.

```
# A `matchit` object
 - method: Subclassification (4 subclasses)
 - distance: Propensity score
             - estimated with logistic regression
 - number of obs.: 72 (original), 72 (matched)
 - target estimand: ATT
 - covariates: Age, NSAID_intol, Previous_Surg, Eosinophil_dic, Allergy, Asthma, Smoke, NPS, SNOT.22
```

```
# Summary of Balance for All Data:
                  Means Treated Means Control Std. Mean Diff. Var. Ratio eCDF Mean eCDF Max
distance                 0.6393        0.2727          1.3171     1.6815    0.3466   0.5940
Age                     57.0968       56.7805          0.0205     1.2273    0.0371   0.0865
NSAID_intolNo            0.7419        0.4634          0.6365          .    0.2785   0.2785
NSAID_intolYes           0.2581        0.5366         -0.6365          .    0.2785   0.2785
Previous_SurgNo          0.3548        0.1463          0.4358          .    0.2085   0.2085
Previous_SurgYes         0.6452        0.8537         -0.4358          .    0.2085   0.2085
Eosinophil_dicNo         0.4516        0.7805         -0.6609          .    0.3289   0.3289
Eosinophil_dicYes        0.5484        0.2195          0.6609          .    0.3289   0.3289
AllergyNo                0.3548        0.3171          0.0789          .    0.0378   0.0378
AllergyYes               0.6452        0.6829         -0.0789          .    0.0378   0.0378
AsthmaNo                 0.1935        0.1951         -0.0040          .    0.0016   0.0016
AsthmaYes                0.8065        0.8049          0.0040          .    0.0016   0.0016
SmokeNo                  0.7742        0.9024         -0.3067          .    0.1282   0.1282
SmokeYes                 0.2258        0.0976          0.3067          .    0.1282   0.1282
NPS                      5.3871        5.7805         -0.2184     1.3379    0.0482   0.1518
SNOT.22                 53.7419       59.5610         -0.3421     0.6779    0.1083   0.2777

# Summary of Balance Across Subclasses
                  Means Treated Means Control Std. Mean Diff. Var. Ratio eCDF Mean eCDF Max
distance                 0.6393        0.5308          0.3900     1.1652    0.1126   0.4194
Age                     57.0968       51.8609          0.3390     1.2654    0.1090   0.2688
NSAID_intolNo            0.7419        0.5118          0.5259          .    0.2301   0.2301
NSAID_intolYes           0.2581        0.4882         -0.5259          .    0.2301   0.2301
Previous_SurgNo          0.3548        0.0846          0.5648          .    0.2703   0.2703
Previous_SurgYes         0.6452        0.9154         -0.5648          .    0.2703   0.2703
Eosinophil_dicNo         0.4516        0.3742          0.1556          .    0.0774   0.0774
Eosinophil_dicYes        0.5484        0.6258         -0.1556          .    0.0774   0.0774
AllergyNo                0.3548        0.3943         -0.0824          .    0.0394   0.0394
AllergyYes               0.6452        0.6057          0.0824          .    0.0394   0.0394
AsthmaNo                 0.1935        0.1018          0.2322          .    0.0918   0.0918
AsthmaYes                0.8065        0.8982         -0.2322          .    0.0918   0.0918
SmokeNo                  0.7742        0.6996          0.1783          .    0.0746   0.0746
SmokeYes                 0.2258        0.3004         -0.1783          .    0.0746   0.0746
NPS                      5.3871        5.7907         -0.2240     1.5231    0.0677   0.1964
SNOT.22                 53.7419       56.8896         -0.1851     0.5246    0.1236   0.2961

# Sample Sizes:
              Control Treated
All             41.        31
Matched (ESS)    7.09      31
Matched         41.        31
Unmatched        0.         0
Discarded        0.         0
```

"*Matched (ESS)*" corresponds to the precision-equivalent number of units after accounting for weight variability, where "*ESS*" stands for "Effective Sample Size" after matching. If matching produces unequal weights, the ESS values will be less than the number of matched observations.

Summary of balance for each subclass is reported below.

```
# Summary of Balance by Subclass:

- Subclass 1
                 Means Treated Means Control Std. Mean Diff. Var. Ratio eCDF Mean eCDF Max
distance                0.2902        0.1589          1.1896     1.1477    0.3333   0.6750
Age                    56.3750       56.1000          0.0129     2.4784    0.1303   0.2750
NSAID_intolNo           0.5000        0.4000          0.2000          .    0.1000   0.1000
NSAID_intolYes          0.5000        0.6000         -0.2000          .    0.1000   0.1000
Previous_SurgNo         0.0000        0.1333         -0.3237          .    0.1333   0.1333
Previous_SurgYes        1.0000        0.8667          0.3237          .    0.1333   0.1333
Eosinophil_dicNo        0.7500        0.8667         -0.2694          .    0.1167   0.1167
Eosinophil_dicYes       0.2500        0.1333          0.2694          .    0.1167   0.1167
AllergyNo               0.2500        0.3333         -0.1925          .    0.0833   0.0833
AllergyYes              0.7500        0.6667          0.1925          .    0.0833   0.0833
AsthmaNo                0.2500        0.2000          0.1155          .    0.0500   0.0500
AsthmaYes               0.7500        0.8000         -0.1155          .    0.0500   0.0500
SmokeNo                 0.8750        0.9333         -0.1764          .    0.0583   0.0583
SmokeYes                0.1250        0.0667          0.1764          .    0.0583   0.0583
NPS                     5.6250        5.9667         -0.2623     1.3308    0.0940   0.2750
SNOT.22                48.6250       64.4333         -1.4330     0.3605    0.2543   0.5500

- Subclass 2
                 Means Treated Means Control Std. Mean Diff. Var. Ratio eCDF Mean eCDF Max
distance                0.4847        0.5559         -1.5684     0.3877    0.2937   0.6667
Age                    53.7143       61.5556         -0.6125     0.6915    0.2444   0.5238
NSAID_intolNo           0.5714        0.6667         -0.1925          .    0.0952   0.0952
NSAID_intolYes          0.4286        0.3333          0.1925          .    0.0952   0.0952
Previous_SurgNo         0.2857        0.2222          0.1405          .    0.0635   0.0635
Previous_SurgYes        0.7143        0.7778         -0.1405          .    0.0635   0.0635
Eosinophil_dicNo        0.5714        0.6667         -0.1925          .    0.0952   0.0952
Eosinophil_dicYes       0.4286        0.3333          0.1925          .    0.0952   0.0952
AllergyNo               0.1429        0.2222         -0.2268          .    0.0794   0.0794
AllergyYes              0.8571        0.7778          0.2268          .    0.0794   0.0794
AsthmaNo                0.1429        0.2222         -0.2268          .    0.0794   0.0794
AsthmaYes               0.8571        0.7778          0.2268          .    0.0794   0.0794
SmokeNo                 0.8571        0.8889         -0.0907          .    0.0317   0.0317
SmokeYes                0.1429        0.1111          0.0907          .    0.0317   0.0317
NPS                     5.5714        5.1111          0.2068     0.7218    0.0567   0.1587
SNOT.22                59.4286       43.4444          0.5556     2.0283    0.1683   0.3492

- Subclass 3
                 Means Treated Means Control Std. Mean Diff. Var. Ratio eCDF Mean eCDF Max
distance                0.7958        0.6472          2.4936          .    0.5000    1.000
Age                    63.3750       52.0000          0.7844          .    0.3125    0.750
NSAID_intolNo           0.8750        0.0000          2.6458          .    0.8750    0.875
NSAID_intolYes          0.1250        1.0000         -2.6458          .    0.8750    0.875
Previous_SurgNo         0.5000        0.0000          1.0000          .    0.5000    0.500
Previous_SurgYes        0.5000        1.0000         -1.0000          .    0.5000    0.500
Eosinophil_dicNo        0.3750        0.0000          0.7746          .    0.3750    0.375
Eosinophil_dicYes       0.6250        1.0000         -0.7746          .    0.3750    0.375
AllergyNo               0.6250        1.0000         -0.7746          .    0.3750    0.375
AllergyYes              0.3750        0.0000          0.7746          .    0.3750    0.375
AsthmaNo                0.1250        0.0000          0.3780          .    0.1250    0.125
AsthmaYes               0.8750        1.0000         -0.3780          .    0.1250    0.125
SmokeNo                 0.8750        0.0000          2.6458          .    0.8750    0.875
SmokeYes                0.1250        1.0000         -2.6458          .    0.8750    0.875
NPS                     5.3750        6.0000         -0.3384          .    0.1562    0.250
SNOT.22                56.1250       37.0000          1.4094          .    0.4219    0.875

- Subclass 4
                 Means Treated Means Control Std. Mean Diff. Var. Ratio eCDF Mean eCDF Max
distance                0.9673        0.7642          9.8227          .    0.5000    1.000
Age                    54.5000       39.0000          1.2572          .    0.5000    1.000
NSAID_intolNo           1.0000        1.0000          0.0000          .    0.0000    0.000
NSAID_intolYes          0.0000        0.0000          0.0000          .    0.0000    0.000
Previous_SurgNo         0.6250        0.0000          1.2910          .    0.6250    0.625
Previous_SurgYes        0.3750        1.0000         -1.2910          .    0.6250    0.625
Eosinophil_dicNo        0.1250        0.0000          0.3780          .    0.1250    0.125
Eosinophil_dicYes       0.8750        1.0000         -0.3780          .    0.1250    0.125
AllergyNo               0.3750        0.0000          0.7746          .    0.3750    0.375
AllergyYes              0.6250        1.0000         -0.7746          .    0.3750    0.375
AsthmaNo                0.2500        0.0000          0.5774          .    0.2500    0.250
AsthmaYes               0.7500        1.0000         -0.5774          .    0.2500    0.250
SmokeNo                 0.5000        1.0000         -1.0000          .    0.5000    0.500
SmokeYes                0.5000        0.0000          1.0000          .    0.5000    0.500
NPS                     5.0000        6.0000         -0.4830          .    0.1667    0.375
SNOT.22                51.5000       81.0000         -2.4064          .    0.5000    1.000

# Sample Sizes by Subclass:
         1  2 3 4 All
Control 30  9 1 1  41
Treated  8  7 8 8  31
Total   38 16 9 9  72
```

The sample size for each subclass is strongly unbalanced in the subclasses 1 (Dupilumab/Mepolizumab: 30/8), 3 (Dupilumab/Mepolizumab: 1/8) and 4 (Dupilumab/Mepolizumab: 1/8), while only in subclass 2 an acceptable numerical balance was returned (Dupilumab/Mepolizumab: 9/7). In **Figure S10** the standardized mean differences/covariate before and after matching, as well as balance for the subclasses, are shown.

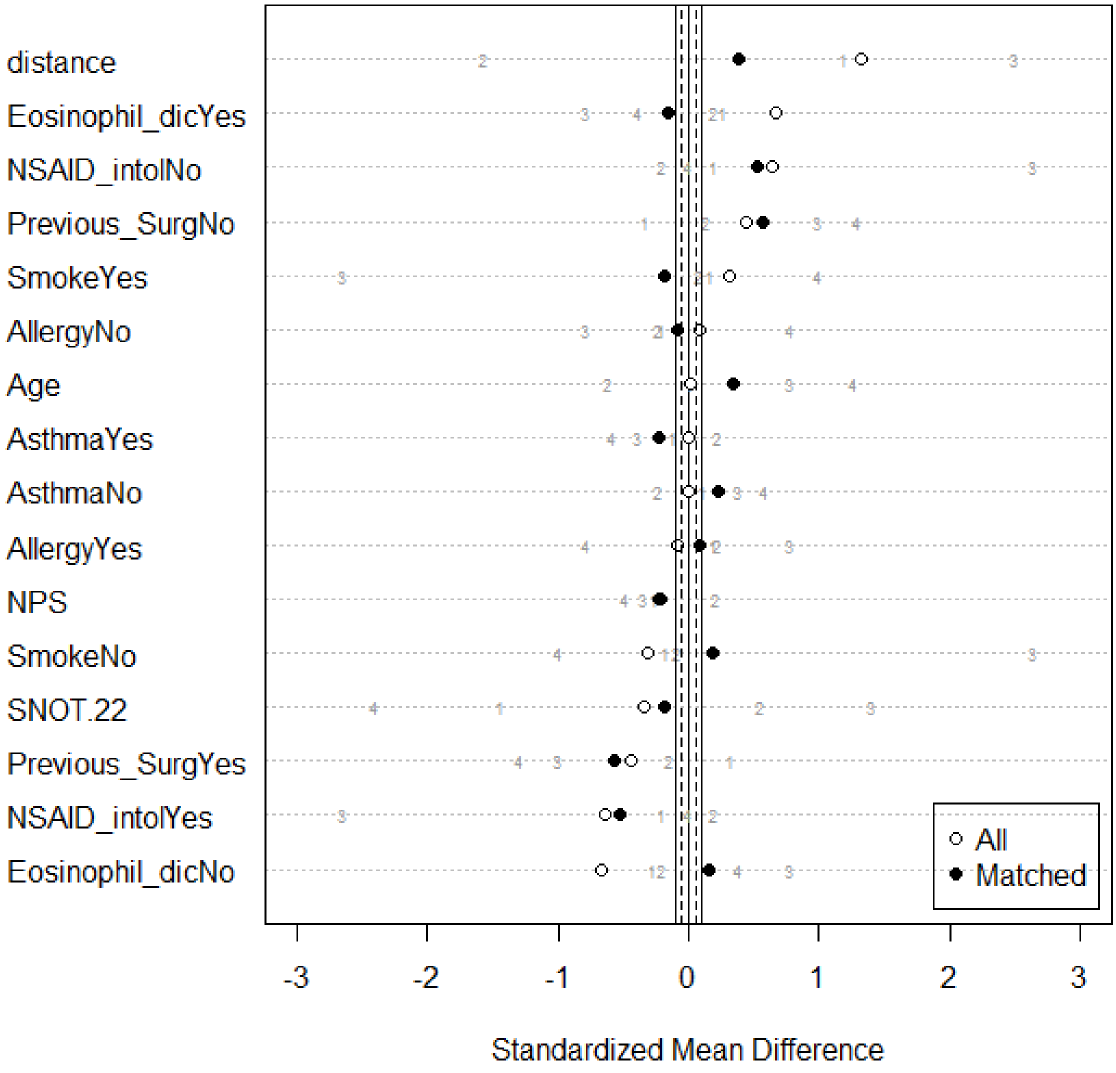

Figure S10 – Standardized mean differences/covariate before/after matching and balance for each subclass.

Since in subclasses 1 and 2 a relatively better numerical balance was observed (Dupilumab/Mepolizumab: 30/8 and 9/7, respectively), we have also evaluated the density plots of the continuous covariates before and after matching (**Figure S11** and **Figure S12**).

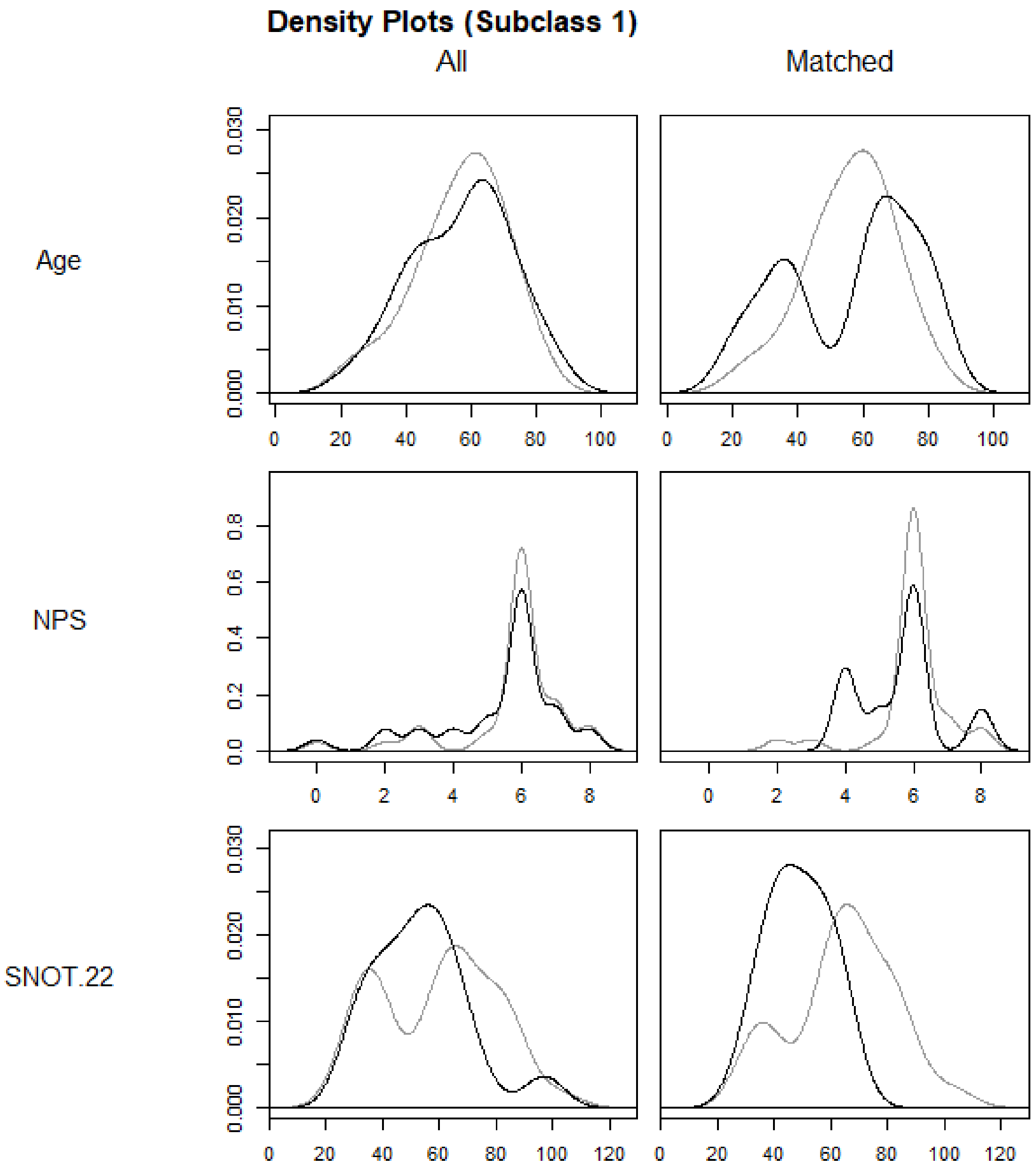

Figure S11 – Density plots for each continuous covariate entered in the PS model (Subclass 1). The *x*-axis displays the covariate values, and the *y*-axis displays the density of the sample at that covariate value. The black line corresponds to the Mepolizumab group and the gray line to the Dupilumab group.

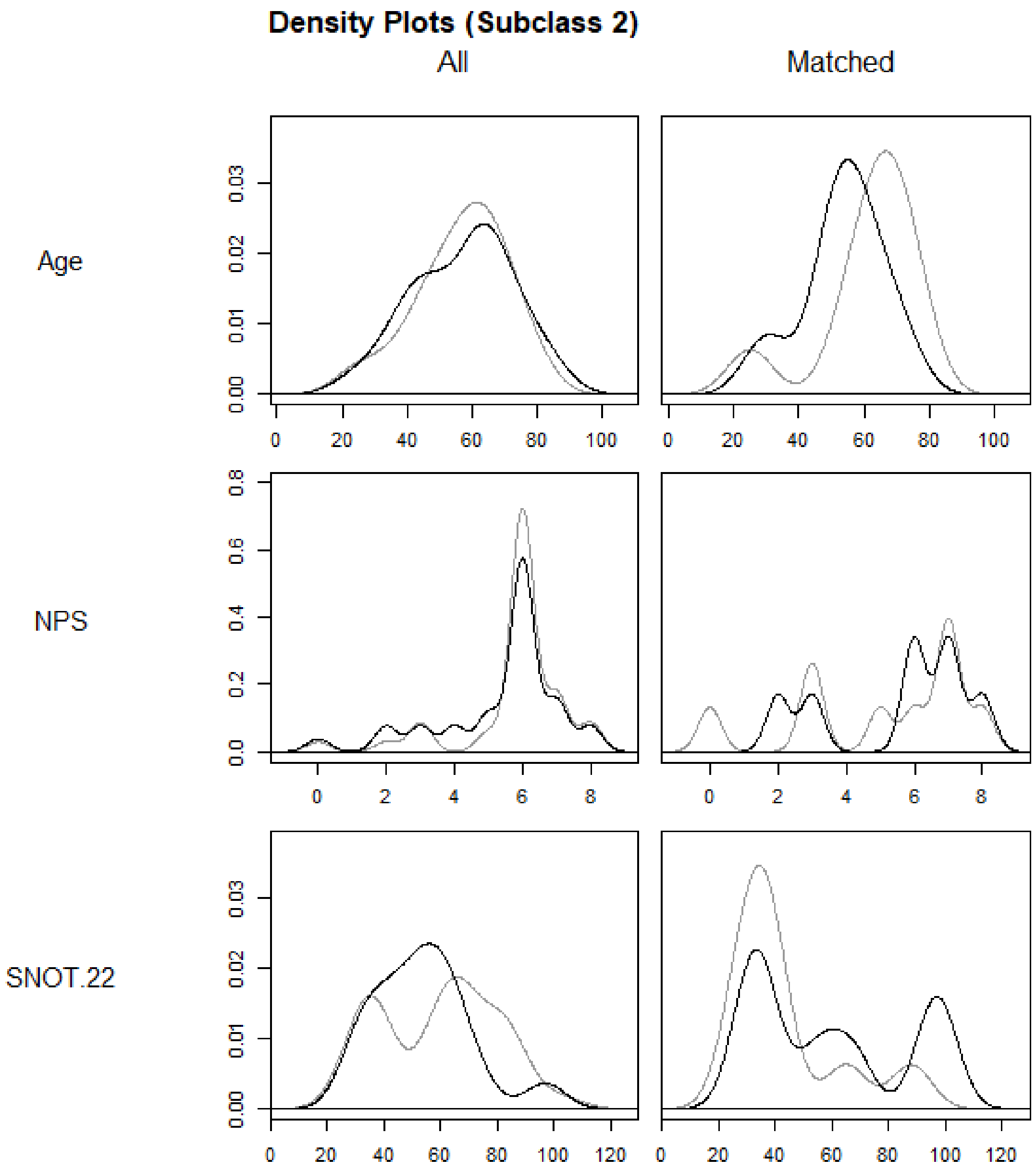

Figure S12 – Density plots for each continuous covariate entered in the PS model (Subclass 2). The *x*-axis displays the covariate values, and the *y*-axis displays the density of the sample at that covariate value. The black line corresponds to the Mepolizumab group and the gray line to the Dupilumab group.

The manifest numerical imbalance in at least three subclasses, as well as the clear worsening observed in the density plots after matching for subclasses 1 and 2, makes this approach completely impractical.

Finally, by taking the original PS model (pp. 29-38), the variables used for matching have been entered into a logistic regression model to estimate the propensity score, by assuming treatment assignment as dependent variable, and covariates that potentially may have influenced treatment assignment as independent variables. Following this approach, we have obtained the output below:

```
Coefficients:
                   Estimate Std. Error z value Pr(>|z|)
(Intercept)        1.446920   2.025368   0.714  0.47498
Age                0.012065   0.022194   0.544  0.58670
NSAID_intolYes    -2.643047   0.876426  -3.016  0.00256 **
Previous_SurgYes  -1.866046   0.815741  -2.288  0.02216 *
Eosinophil_dicYes  2.692700   0.840328   3.204  0.00135 **
AllergyYes         0.886542   0.760578   1.166  0.24377
AsthmaYes         -0.388531   0.906789  -0.428  0.66831
SmokeYes           1.664123   0.998497   1.667  0.09559 .
NPS                0.005259   0.195764   0.027  0.97857
SNOT.22           -0.025900   0.016544  -1.566  0.11746 ---
Signif. codes: 0 '***' 0.001 '**' 0.01 '*' 0.05 '.' 0.1 ' ' 1

(Dispersion parameter for binomial family taken to be 1)

    Null deviance: 98.420 on 71 degrees of freedom
Residual deviance: 67.261 on 62 degrees of freedom
AIC: 87.261

Number of Fisher Scoring iterations: 5

                           OR      2.5 %      97.5 %
(Intercept)        4.25000382 0.08382669 278.3916512
Age                1.01213834 0.96964370   1.0595361
NSAID_intolYes     0.07114418 0.01014853   0.3368752
Previous_SurgYes   0.15473425 0.02680679   0.7001247
Eosinophil_dicYes 14.77150996 3.31489798  95.2483408
AllergyYes         2.42672460 0.57941066  11.9540678
AsthmaYes          0.67805213 0.10880930   4.0449876
SmokeYes           5.28103721 0.80670836  43.9611942
NPS                1.00527292 0.68035503   1.4964046
SNOT.22            0.97443301 0.94149899   1.0055179
```

In the logistic regression model, the predicted values of the probability for treatment assignment corresponds essentially to the propensity score. By using the `augment_columns` and `mutate` functions, the same results were returned regarding descriptive statistics (min/max values, mean, median, 1st and 3rd quartile) of the propensity score.

```
## Method 1: Using augment_columns function
prob_fitted <- augment_columns(logit1, data = PSM, type.predict = "response") %>%
               rename(propensity_score = .fitted)

## Inspect propensity scores (propensity_score)
summary(prob_fitted$propensity_score)

   Min. 1st Qu.  Median    Mean 3rd Qu.    Max.
0.01693 0.18369 0.36639 0.43056 0.64118 0.98720


## Method 2: Using mutate function
prob_fitted2 <- PSM %>%
    mutate(propensity_score = glm(GroupLR ~ Age + NSAID_intol + Previous_Surg + Eosinophil.dic +
Allergy + Asthma + Smoke + NPS + SNOT.22, data = PSM, family = "binomial"(link = "logit")) %>%
    predict(type = "response"))

## Inspect propensity scores (propensity_score)
summary(prob_fitted2$propensity_score)

   Min. 1st Qu.  Median    Mean 3rd Qu.    Max.
0.01693 0.18369 0.36639 0.43056 0.64118 0.98720
```

The new matched dataset (n = 62), with 31 cases for each treatment group (see "`# Sample size`" in the R output at p. 30), has been further evaluated for covariate balancing, by using the same approach previously described (pp. 29-31). In this additional step, no other unmatched cases were returned by the statistical environment (data not shown). The matched dataset obtained by applying the PS method has been used for a new round of statistical analysis (see the next Sections S6-S7).

Section S6. Statistical analysis carried out in the matched sample obtained by the Propensity Score

The matched sample obtained by applying the propensity score (PS), that returned n = 31 cases for both Dupilumab and Mepolizumab group, was subjected to further statistical analyses.

S6.1 – Extended descriptive statistics for continuous and categorical variables in the matched sample

Table S21 – Descriptive statistics for continuous variables of the matched sample (all cases; **Part I**).

	Age	Cycles_N	NPS	SNOT-22	SSIT-16	IgE_Tot
**Mean**	57.048	4.032	5.532	55.903	4.774	368.925
**Std. error mean**	1.820	0.948	0.226	0.235	0.321	83.873
**95% CI mean lower bound**	53.482	2.173	5.089	51.297	4.144	204.538
**95% CI mean upper bound**	60.615	5.891	5.976	60.510	5.404	533.312
**Median**	60.000	1.000	6.000	56.000	5.000	171.000
**Standard deviation**	14.328	7.468	1.781	18.505	2.531	604.813
**IQR**	19.750	3.000	1.000	28.500	3.000	254.000
**Range**	63	30	8	72	10	2883.500
**Minimum**	23	0	0	26	0	2.500
**Maximum**	86	30	8	98	10	2886.000
**Skewness**	-0.471	2.320	-1.360	0.328	0.300	2.782
**Std. error skewness**	0.304	0.304	0.304	0.304	0.304	0.330
**Kurtosis**	-0.095	4.195	1.858	-0.642	-0.613	7.618
**Std. error kurtosis**	0.599	0.599	0.599	0.599	0.599	0.650
**Shapiro-Wilk (*W*)**	0.973	0.572	0.820	0.962	0.962	0.587
**Shapiro-Wilk (*P* value)***	0.181	< .001	< .001	0.051	0.055	< .001

* The Shapiro-Wilk test evaluates the normal distribution of continuous variables. In this test, a P value < 0.05 indicates that the data significantly deviates from a normal distribution (reject the null hypothesis of a normal distribution), while a P value > 0.05 suggests the data does not significantly differ from normal distribution (fail to reject the null hypothesis). When a P value < 0.05 is returned by the Shapiro-Wilk test, a non-parametric test should be used in the subsequent comparisons.

Table S21 – Descriptive statistics for continuous variables of the matched sample (all cases; **Part II**).

	EOS_or*	EOS_mi**	ACT	VAS	Lund-Mackay	Access
**Mean**	621.700	618.581	19.415	42.167	18.574	11.328
**Std. error mean**	45.211	43.795	0.670	2.091	0.609	1.110
**95% CI mean lower bound**	533.089	532.744	18.101	38.069	17.380	9.152
**95% CI mean upper bound**	710.311	704.417	20.729	46.264	19.767	13.504
**Median**	555.000	525.000	21	44.500	20	7
**Standard deviation**	350.200	344.841	4.881	15.362	4.756	8.671
**IQR**	487.500	460.000	8.000	23.750	7.000	14.000
**Range**	1590	1590	19	67	26	24
**Minimum**	100	100	6	9	2	0
**Maximum**	1690	1690	25	76	28	24
**Skewness**	0.962	1.001	-0.867	0.015	-0.917	0.335
**Std. error skewness**	0.309	0.304	0.327	0.325	0.306	0.306
**Kurtosis**	0.659	0.799	0.096	-0.711	1.222	-1.451
**Std. error kurtosis**	0.608	0.599	0.644	0.639	0.604	0.604
**Shapiro-Wilk (*W*)**	0.929	0.927	0.912	0.977	0.932	0.865
**Shapiro-Wilk (*P* value)**	0.002	0.001	< .001	0.366	0.002	< .001

* "*EOS_or*": eosinophil count (cells/µL) with $n = 3$ missing values in the Dupilumab group (raw data).
** "*EOS_mi*": eosinophil count (cells/µL) after replacement of missing values in the Dupilumab group by using a simple missing imputation (mi) method. For more details, see Section S4.1 (pp. 14-16).

Table S22 – Descriptive statistics for continuous variables of the matched sample, by grouping for Dupilumab and Mepolizumab (**Part I**).

	Group	Age	Cycles_N	NPS	SNOT-22
**Mean**	Dupilumab	57.000	6.065	5.677	58.065
	Mepolizumab	57.097	2.000	5.387	53.742
**Std. error mean**	Dupilumab	2.403	1.692	0.319	3.580
	Mepolizumab	2.774	0.721	0.324	3.055
**95% CI mean lower bound**	Dupilumab	52.291	2.749	5.052	51.048
	Mepolizumab	51.660	0.586	4.753	47.754
**95% CI mean upper bound**	Dupilumab	61.709	9.380	6.303	65.081
	Mepolizumab	62.533	3.414	6.021	59.730
**Median**	Dupilumab	60	2	6	59
	Mepolizumab	61	1	6	54
**Standard deviation**	Dupilumab	13.377	9.420	1.777	19.933
	Mepolizumab	15.443	4.017	1.801	17.010
**IQR**	Dupilumab	17.000	5.000	0.500	32.000
	Mepolizumab	21.500	2.000	1.000	21.000
**Range**	Dupilumab	55	30	8	66
	Mepolizumab	63	20	8	69
**Minimum**	Dupilumab	23	0	0	26
	Mepolizumab	23	0	0	29
**Maximum**	Dupilumab	78	30	8	92
	Mepolizumab	86	20	8	98
**Skewness**	Dupilumab	-0.831	1.595	-1.531	-0.054
	Mepolizumab	-0.253	3.494	-1.285	0.822
**Std. error skewness**	Dupilumab	0.421	0.421	0.421	0.421
	Mepolizumab	0.421	0.421	0.421	0.421
**Kurtosis**	Dupilumab	0.686	0.928	2.711	-1.269
	Mepolizumab	-0.486	13.856	1.703	0.964
**Std. error kurtosis**	Dupilumab	0.821	0.821	0.821	0.821
	Mepolizumab	0.821	0.821	0.821	0.821
**Shapiro-Wilk (W)**	Dupilumab	0.946	0.650	0.800	0.931
	Mepolizumab	0.981	0.533	0.845	0.936
**Shapiro-Wilk (*P* value)**	Dupilumab	0.125	< .001	< .001	0.046
	Mepolizumab	0.839	< .001	< .001	0.063

Table S22 – Descriptive statistics for continuous variables of the matched sample, by grouping for Dupilumab and Mepolizumab (**Part II**).

	Group	SSIT-16	IgE_Tot	EOS_or*	EOS_mi**
**Mean**	Dupilumab	4.484	375.777	542.828	541.677
	Mepolizumab	5.065	362.073	695.484	695.484
**Std. error mean**	Dupilumab	0.497	122.732	57.199	53.453
	Mepolizumab	0.409	116.766	67.441	67.441
**95% CI mean lower bound**	Dupilumab	3.510	135.227	430.720	436.912
	Mepolizumab	4.262	133.216	563.301	563.301
**95% CI mean upper bound**	Dupilumab	5.458	616.327	654.935	646.443
	Mepolizumab	5.867	590.930	827.667	827.667
**Median**	Dupilumab	5	202.000	500	500
	Mepolizumab	5	133.500	650	650
**Standard deviation**	Dupilumab	2.767	625.812	308.024	297.613
	Mepolizumab	2.279	595.392	375.498	375.498
**IQR**	Dupilumab	3.500	274.500	400.000	360.000
	Mepolizumab	3.000	209.250	425.000	425.000
**Range**	Dupilumab	10	2883.500	1120	1120
	Mepolizumab	9	2275.400	1590	1590
**Minimum**	Dupilumab	0	2.500	190	190
	Mepolizumab	1	11.600	100	100
**Maximum**	Dupilumab	10	2886.000	1310	1310
	Mepolizumab	10	2287.000	1690	1690
**Skewness**	Dupilumab	0.367	3.247	0.983	1.024
	Mepolizumab	0.403	2.422	0.877	0.877
**Std. error skewness**	Dupilumab	0.421	0.456	0.434	0.421
	Mepolizumab	0.421	0.456	0.421	0.421
**Kurtosis**	Dupilumab	-0.862	11.176	0.409	0.662
	Mepolizumab	-0.125	5.271	0.583	0.583
**Std. error kurtosis**	Dupilumab	0.821	0.887	0.845	0.821
	Mepolizumab	0.821	0.887	0.821	0.821
**Shapiro-Wilk (*W*)**	Dupilumab	0.936	0.555	0.905	0.906
	Mepolizumab	0.954	0.603	0.937	0.937
**Shapiro-Wilk (*P* value)**	Dupilumab	0.063	< .001	0.013	0.010
	Mepolizumab	0.195	< .001	0.067	0.067

* "*EOS_or*": eosinophil count (cells/µL) with $n = 3$ missing values in the Dupilumab group (raw data).
** "*EOS_mi*": eosinophil count (cells/µL) after replacement of missing values in the Dupilumab group by using a simple missing imputation (mi) method. For more details, see Section S4.1 (pp. 14-16).

Table S22 – Descriptive statistics for continuous variables of the matched sample, by grouping for Dupilumab and Mepolizumab (**Part III**).

	Group	ACT	VAS	Lund-Mackay	Access
**Mean**	Dupilumab	19.185	44.071	18.774	11.161
	Mepolizumab	19.654	40.115	18.367	11.500
**Std. error mean**	Dupilumab	0.983	2.966	0.862	1.503
	Mepolizumab	0.926	2.947	0.873	1.664
**95% CI mean lower bound**	Dupilumab	17.259	38.259	17.084	8.215
	Mepolizumab	17.838	34.339	16.656	8.240
**95% CI mean upper bound**	Dupilumab	21.111	49.884	20.464	14.108
	Mepolizumab	21.470	45.892	20.078	14.760
**Median**	Dupilumab	21	46.500	20	7
	Mepolizumab	21.000	35.000	19.500	10.000
**Standard deviation**	Dupilumab	5.107	15.694	4.801	8.371
	Mepolizumab	4.724	15.029	4.781	9.111
**IQR**	Dupilumab	7.000	23.250	6.000	14.000
	Mepolizumab	7.000	19.750	7.000	15.500
**Range**	Dupilumab	19	67	22	24
	Mepolizumab	17	54	20	24
**Minimum**	Dupilumab	6	9	2	0
	Mepolizumab	8	12	8	0
**Maximum**	Dupilumab	25	76	24	24
	Mepolizumab	25	66	28	24
**Skewness**	Dupilumab	-0.964	-0.141	-1.525	0.489
	Mepolizumab	-0.776	0.169	-0.337	0.217
**Std. error skewness**	Dupilumab	0.448	0.441	0.421	0.421
	Mepolizumab	0.456	0.456	0.427	0.427
**Kurtosis**	Dupilumab	0.258	-0.336	3.468	-1.333
	Mepolizumab	0.045	-0.949	-0.450	-1.585
**Std. error kurtosis**	Dupilumab	0.872	0.858	0.821	0.821
	Mepolizumab	0.887	0.887	0.833	0.833
**Shapiro-Wilk W**	Dupilumab	0.905	0.978	0.861	0.862
	Mepolizumab	0.915	0.953	0.964	0.861
**Shapiro-Wilk p**	Dupilumab	0.017	0.810	< .001	< .001
	Mepolizumab	0.034	0.279	0.394	0.001

Table S23 – Descriptive statistics for categorical variables of the matched sample (**Part I**)

Frequencies of Group

Levels	Counts	% of Total	Cumulative %
Dupilumab	31	50.0 %	50.0 %
Mepolizumab	31	50.0 %	100.0 %

Frequencies of Gender

Levels	Counts	% of Total	Cumulative %
Female	36	58.1 %	58.1 %
Male	26	41.9 %	100.0 %

Frequencies of NSAID_intol

Levels	Counts	% of Total	Cumulative %
No	40	64.5 %	64.5 %
Yes	22	35.5 %	100.0 %

Frequencies of Previous_Surg

Levels	Counts	% of Total	Cumulative %
No	17	27.4 %	27.4 %
Yes	45	72.6 %	100.0 %

Frequencies of Medical_ther

Levels	Counts	% of Total	Cumulative %
No	5	8.1 %	8.1 %
Yes	57	91.9 %	100.0 %

Table S23 – Descriptive statistics for categorical variables of the matched sample (**Part II**)

Frequencies of OS_ther

Levels	Counts	% of Total	Cumulative %
No	22	35.5 %	35.5 %
Yes	40	64.5 %	100.0 %

Frequencies of Allergy

Levels	Counts	% of Total	Cumulative %
No	22	35.5 %	35.5 %
Yes	40	64.5 %	100.0 %

Frequencies of Asthma

Levels	Counts	% of Total	Cumulative %
No	12	19.4 %	19.4 %
Yes	50	80.6 %	100.0 %

Frequencies of Smoke

Levels	Counts	% of Total	Cumulative %
No	51	82.3 %	82.3 %
Yes	11	17.7 %	100.0 %

Frequencies of RM

Levels	Counts	% of Total	Cumulative %
No	59	95.2 %	95.2 %
Yes	3	4.8 %	100.0 %

Table S23 – Descriptive statistics for categorical variables of the matched sample (**Part III**)

Frequencies of Asthma_control

Levels	Counts	% of Total	Cumulative %
Good	30	56.6 %	56.6 %
Partly	13	24.5 %	81.1 %
Poorly	10	18.9 %	100.0 %

Frequencies of EarlyCompl

Levels	Counts	% of Total	Cumulative %
No	58	93.5 %	93.5 %
Yes	4	6.5 %	100.0 %

Frequencies of LateCompl

Levels	Counts	% of Total	Cumulative %
No	56	90.3 %	90.3 %
Yes	6	9.7 %	100.0 %

Frequencies of Complications

Levels	Counts	% of Total	Cumulative %
No	54	87.1 %	87.1 %
Yes	8	12.9 %	100.0 %

S6.2 – Comparisons of continuous and categorical variables in the matched sample

Table S24 – Comparison between continuous variables by stratifying for Dupilumab *vs*. Mepolizumab in the matched sample.

		Statistic	p
Age	Mann-Whitney U	479.500	0.994
Cycles_N	Mann-Whitney U	351.000	0.061
NPS	Mann-Whitney U	424.500	0.402
SNOT.22	Mann-Whitney U	403.000	0.278
SSIT.16	Mann-Whitney U	404.500	0.284
IgE_Tot	Mann-Whitney U	299.000	0.481
Eosinophil_or	Mann-Whitney U	333.000	0.086
Eosinophil_mi	Mann-Whitney U	359.000	0.088
ACT	Mann-Whitney U	332.000	0.741
VAS	Mann-Whitney U	316.000	0.411
Lund.Mackay	Mann-Whitney U	425.500	0.572
Access	Mann-Whitney U	458.000	0.925

Group Descriptives

	Group	N	Mean	Median	SD	SE
Age	Dupilumab	31	57.000	60.000	13.377	2.403
	Mepolizumab	31	57.097	61.000	15.443	2.774
Cycles_N	Dupilumab	31	6.065	2.000	9.420	1.692
	Mepolizumab	31	2.000	1.000	4.017	0.721
NPS	Dupilumab	31	5.677	6.000	1.777	0.319
	Mepolizumab	31	5.387	6.000	1.801	0.324
SNOT.22	Dupilumab	31	58.065	59.000	19.933	3.580
	Mepolizumab	31	53.742	54.000	17.010	3.055
SSIT.16	Dupilumab	31	4.484	5.000	2.767	0.497
	Mepolizumab	31	5.065	5.000	2.279	0.409
IgE_Tot	Dupilumab	26	375.777	202.000	625.812	122.732
	Mepolizumab	26	362.073	133.500	595.392	116.766
Eosinophil_or	Dupilumab	29	542.828	500.000	308.024	57.199
	Mepolizumab	31	695.484	650.000	375.498	67.441
Eosinophil_mi	Dupilumab	31	541.677	500.000	297.613	53.453
	Mepolizumab	31	695.484	650.000	375.498	67.441
ACT	Dupilumab	27	19.185	21.000	5.107	0.983
	Mepolizumab	26	19.654	21.000	4.724	0.926
VAS	Dupilumab	28	44.071	46.500	15.694	2.966
	Mepolizumab	26	40.115	35.000	15.029	2.947
Lund.Mackay	Dupilumab	31	18.774	20.000	4.801	0.862
	Mepolizumab	30	18.367	19.500	4.781	0.873
Access	Dupilumab	31	11.161	7.000	8.371	1.503
	Mepolizumab	30	11.500	10.000	9.111	1.664

Table S25 – Recalculated Table 1 of the paper in the matched sample.

[Table 1. Demographic data of patients included in the study**]**.

**Variable**		**Overall (n = 62)**	**Dupilumab (n = 31)**	**Mepolizumab (n = 31)**	**P-value**
**Patients**			31/62 (50%)	31/22 (50%)	1.000
**Age**		57.05 ± 14.33	57 ± 13.38	57.1 ±15.44	0.994
**Gender**	Male	26 (41.9%)	17 (54.8%)	9 (29%)	0.071
	Female	36 (58.1%)	14 (45.2%)	22 (71%)	
**Previous endoscopic sinus surgery**	Yes	45 (72.6%)	25 (80.6%)	20 (64.5%)	0.255
	No	17 (27.4%)	6 (19.4%)	11 (35.5%)	
**Number of cycles of SCS in the previous year**		4.03 ± 7.47	6.06 ± 9.42	2.0 ± 4.02	**0.061**
**Allergy**	Yes	40 (64.5%)	20 (64.5%)	20 (64.5%)	1.000
	No	22 (35.5%)	11 (35.5%)	11 (35.5%)	
**Asthma**	Yes	50 (80.6%)	25 (80.6%)	25 (80.6%)	1.000
	No	12 (19.4%)	6 (19.4%)	6 (19.4%)	
**NSAID intolerance**	Yes	22 (35.5%)	14 (45.2%)	8 (25.8%)	**0.184**
	No	40 (64.5%)	17 (54.8%)	23 (74.2%)	

Results represent the mean ± SD and number of events (%) for continuous and categorical variables, respectively.
SCS: systemic corticosteroids; NSAID: non steroid anti-inflammatory drugs.

* *P* value column refers to comparison between Dupilumab and Mepolizumab in the matched groups. The *P* values recalculated in the matched sample and that originally were <0.05 in Table 1 of the paper (unmatched sample) are shown in bold.

Table S26 – Recalculated Table 2 of the paper in the matched sample.

[**Table 2**. Clinical and radiological scores, blood test results, and endoscopic assessment of NPS collected before starting biological therapy].

Variable*	Dupilumab (n = 31)	Mepolizumab (n = 31)	P-value**
**NPS**	5.68 ± 1.77 (6.0)	5.39 ± 1.80 (6.0)	0.402
**SNOT-22**	58.06 ± 19.93 (59.0)	53.74 ± 17.01 (54.0)	0.278
**SSIT-16**	4.48 ± 2.77 (5.0)	5.07 ± 2.28 (5.0)	0.284
**IgE Tot (kU/L)**	375.78 ± 625.81 (202)	362.07 ± 595.39 (133.5)	0.481
**Eosinophil count (cells/µL)**	542.83 ± 308.02 (500)	695.48 ± 375.50 (650)	**0.086**
**Eosinophil (%)**	8.67 ± 6.92 (7.4)	9.77 ± 4.89 (8.4)	0.218
**ACT**	19.18 ± 5.11 (21.0)	19.65 ± 4.72 (21.0)	0.741
**VAS**	44.07 ± 15.69 (46.5)	40.12 ± 15.03 (35.0)	0.411
**Lund-Mackay**	18.77 ± 4.8 (20.0)	18.37 ± 4.78 (19.5)	0.572
**Access**	11.16 ± 8.37 (7.0)	11.50 ± 9.11 (10.0)	0.925

* The results of each variable are expressed as mean ± SD (median).

** The *P* values recalculated in the matched sample and that originally were <0.05 in Table 1 of the paper (unmatched sample) are shown in bold.

SNOT-22: Sinonasal Outcome Test-22; SSIT-16: Sniffin' Sticks-16 Identification Test; ACT: Asthma Control Test; NPS: Nasal Polyp Score

After matching, the significant differences reported in Table 1-2 of the paper have not been confirmed ("*Number of cycles of SCS in the previous year*": *P* value from 0.024 to 0.061; "*NSAID intolerance*": *P* value from 0.029 to 0.184; "*Eosinophil count*": *P* value from 0.039 to 0.086).

The treatment to which the variable "*Eosinophil count*" was subjected (see Section S4.1, pp. 14-18), with the creation of two new additional variables ("*Eosinophil_mi*", where the original n = 3 missing vales in the Dupilumab group at the baseline were added after missing imputation; "*Eosinophil_dic*", where the patients of the "*Eosinophil_mi*" variable were encoded as "*No/Yes*" when eosinophil count resulted ≤ 600 or > 600 cells/µL, respectively), has suggested to summarize the results obtained in the matched sample for the eosinophil-related variables following the same approach used in the previous Section S4.1 (**Table S27**).

Table S27 – Extended descriptive statistics for the Eosinophil-related continuous variables in the matched sample.

Descriptives

	Eosinophil_or	Eosinophil_mi	Eosinophil_perc
N	60	62	54
Mean	621.700	618.581	9.270
Std. error mean	45.211	43.795	0.756
95% CI mean lower bound	533.089	532.744	7.790
95% CI mean upper bound	710.311	704.417	10.751
Median	555.000	525.000	8.200
Standard deviation	350.200	344.841	5.552
IQR	487.500	460.000	7.375
Range	1590	1590	30.400
Minimum	100	100	1.100
Maximum	1690	1690	31.500
Skewness	0.962	1.001	1.389
Std. error skewness	0.309	0.304	0.325
Kurtosis	0.659	0.799	3.547
Std. error kurtosis	0.608	0.599	0.639
Shapiro-Wilk W	0.929	0.927	0.907
Shapiro-Wilk p	0.002	0.001	< .001

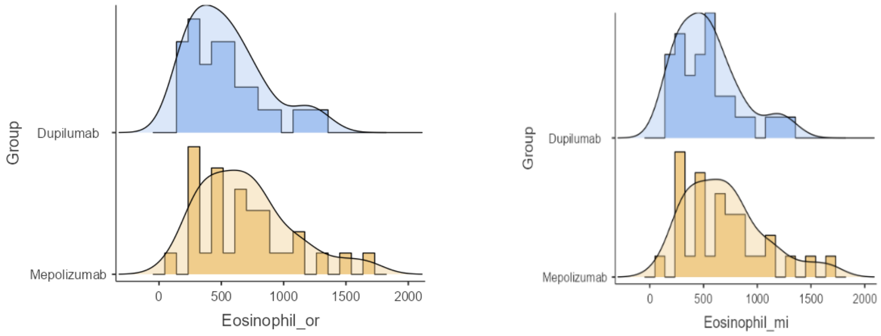

Figure S13 – Histogram with density plot for "*Eosinophil_or*" (original data, with $n = 3$ missing values in the Dupilumab group) and "*Eosinophil_mi*" (data after mean imputation for the missing cases in the Dupilumab group) variables, in both Dupilumab and Mepolizumab group of the matched sample.

The differences between comparisons for treatment with raw data and after missing imputation in the matched sample are reported in **Table S28**.

Table S28 – Mann-Whitney test performed by comparing Eosinophil count (cells/μL) in Dupilumab *vs.* Mepolizumab group ("*Eosinophil_or*": raw data; "*Eosinophil_mi*": data after mean imputation for n = 3 missing values in the Dupilumab group) in the matched sample.

						95% Confidence Interval	
		Statistic	p	Mean difference	SE difference	Lower	Upper
Eosinophil_or	Mann-Whitney U	333.000	0.086	−130.000		−300.000	10.000
Eosinophil_mi	Mann-Whitney U	359.000	0.088	−130.000		−300.000	10.000

Group Descriptives

	Group	N	Mean	Median	SD	SE
Eosinophil_or	Dupilumab	29	542.828	500.000	308.024	57.199
	Mepolizumab	31	695.484	650.000	375.498	67.441
Eosinophil_mi	Dupilumab	31	541.677	500.000	297.613	53.453
	Mepolizumab	31	695.484	650.000	375.498	67.441

By assuming the eosinophil count > 600 cells/μL as cut-off value (ref. variable: "*Eosinophil.mi*"), we have created the "*Eosinophil_dic*" variable (encoded as "*Yes*": > 600 cells/μL; "*No*": ≤ 600 cells/μL). The main frequency statistics and binomial test for this new variable are reported in **Table S29**.

Table S29 – Frequency statistics and binomial test for the "*Eosinophil_dic*" variable in the matched sample.

Frequencies of Eosinophil_dic

Levels	Counts	% of Total	Cumulative %
No	36	58.1 %	58.1 %
Yes	26	41.9 %	100.0 %

Binomial Test

						95% Confidence Interval	
	Level	Count	Total	Proportion	p	Lower	Upper
Eosinophil_dic	No	36	62	0.581	0.253	0.448	0.705
	Yes	26	62	0.419	0.253	0.295	0.552

Note. H_a is proportion ≠ 0.5

The “*Eosinophil_dic*” has been used as stratifying variable for several comparisons (**Table S30**, **Table S31**).

Table S30 – Comparison between Dupilumab *vs*. Mepolizumab for “*Eosinophil_dic*” variable in the matched sample.

Contingency Tables

		Group		
Eosinophil_dic		Dupilumab	Mepolizumab	Total
No	Observed	22	14	36
	% within row	61.1 %	38.9 %	100.0 %
	% within column	71.0 %	45.2 %	58.1 %
	% of total	35.5 %	22.6 %	58.1 %
Yes	Observed	9	17	26
	% within row	34.6 %	65.4 %	100.0 %
	% within column	29.0 %	54.8 %	41.9 %
	% of total	14.5 %	27.4 %	41.9 %
Total	Observed	31	31	62
	% within row	50.0 %	50.0 %	100.0 %
	% within column	100.0 %	100.0 %	100.0 %
	% of total	50.0 %	50.0 %	100.0 %

χ^2 Tests

	Value	df	p
χ^2	4.239	1	0.039
Fisher's exact test			0.071
N	62		

Table S31 – Comparison between the main continuous variables by stratifying for absence (No) / presence (Yes) of eosinophil count > 600 cells/μL in the matched sample.

		Statistic	p
Age	Mann-Whitney U	347.000	0.085
Cycles_N	Mann-Whitney U	467.500	1.000
NPS	Mann-Whitney U	402.000	0.317
SNOT.22	Mann-Whitney U	388.000	0.257
SSIT.16	Mann-Whitney U	438.000	0.671
IgE_Tot	Mann-Whitney U	322.500	0.897
ACT	Mann-Whitney U	261.500	0.115
VAS	Mann-Whitney U	288.000	0.234
Lund.Mackay	Mann-Whitney U	454.500	1.000
Access	Mann-Whitney U	419.000	0.602

Group Descriptives

	Group	N	Mean	Median	SD	SE
Age	No	36	60.056	61.500	11.536	1.923
	Yes	26	52.885	59.000	16.839	3.302
Cycles_N	No	36	4.111	1.000	7.652	1.275
	Yes	26	3.923	1.000	7.353	1.442
NPS	No	36	5.667	6.000	1.805	0.301
	Yes	26	5.346	6.000	1.765	0.346
SNOT.22	No	36	53.167	55.000	18.488	3.081
	Yes	26	59.692	57.000	18.203	3.570
SSIT.16	No	36	4.778	4.000	2.929	0.488
	Yes	26	4.769	5.000	1.904	0.373
IgE_Tot	No	30	437.637	173.500	724.457	132.267
	Yes	22	275.227	171.000	384.256	81.924
ACT	No	28	20.286	21.000	4.958	0.937
	Yes	25	18.440	20.000	4.700	0.940
VAS	No	31	40.032	44.000	15.855	2.848
	Yes	23	45.043	47.000	14.515	3.027
Lund.Mackay	No	35	18.371	20.000	5.292	0.895
	Yes	26	18.846	20.000	4.007	0.786
Access	No	35	11.457	7.000	8.350	1.411
	Yes	26	11.154	10.500	9.251	1.814

S6.3 – Additional analyses for NSAID intolerance, Asthma Control Test (ACT) and EPOS in the matched sample

In this section is replicated on the matched sample a part of the analyses originally performed in the unmatched sample (for more details, see Sections S4.2-S4.4, pp. 19-28).

Table S32 – Comparison between the main continuous variables by stratifying for absence (No) / presence (Yes) of NSAID intolerance in the matched sample.

		Statistic	p
Age	Mann-Whitney U	362.000	0.254
Cycles_N	Mann-Whitney U	371.000	0.298
NPS	Mann-Whitney U	381.500	0.360
SNOT.22	Mann-Whitney U	436.500	0.965
SSIT.16	Mann-Whitney U	422.000	0.795
IgE_Tot	Mann-Whitney U	194.500	0.024
Eosinophil_or	Mann-Whitney U	198.000	0.001
Eosinophil_mi	Mann-Whitney U	219.500	0.001
ACT	Mann-Whitney U	244.000	0.080
VAS	Mann-Whitney U	321.500	0.971
Lund.Mackay	Mann-Whitney U	354.000	0.261
Access	Mann-Whitney U	411.500	0.797

Group Descriptives

	Group	N	Mean	Median	SD	SE
Age	No	40	58.900	61.500	12.133	1.918
	Yes	22	53.682	58.000	17.450	3.720
Cycles_N	No	40	3.825	1.000	6.994	1.106
	Yes	22	4.409	0.500	8.421	1.795
NPS	No	40	5.375	6.000	1.807	0.286
	Yes	22	5.818	6.000	1.736	0.370
SNOT.22	No	40	55.275	56.500	16.840	2.663
	Yes	22	57.045	56.000	21.586	4.602
SSIT.16	No	40	4.875	5.000	2.662	0.421
	Yes	22	4.591	5.000	2.323	0.495
IgE_Tot	No	33	255.073	110.000	409.857	71.347
	Yes	19	566.668	213.000	820.217	188.171
Eosinophil_or	No	39	518.205	470.000	309.939	49.630
	Yes	21	813.905	730.000	345.821	75.464
Eosinophil_mi	No	40	518.375	485.000	305.942	48.374
	Yes	22	800.773	730.000	343.062	73.141
ACT	No	31	20.387	21.000	4.580	0.823
	Yes	22	18.045	19.500	5.066	1.080
VAS	No	36	42.444	45.000	14.943	2.490
	Yes	18	41.611	39.000	16.600	3.913
Lund.Mackay	No	39	18.051	19.000	5.062	0.811
	Yes	22	19.500	20.500	4.103	0.875
Access	No	39	11.308	7.000	8.323	1.333
	Yes	22	11.364	9.500	9.459	2.017

Table S33 – Comparison between absence/presence of NSAID intolerance and absence/presence of asthma in the matched sample.

Contingency Tables

Asthma		NSAID_intol No	NSAID_intol Yes	Total
No	Observed	12	0	12
	% within row	100.0 %	0.0 %	100.0 %
	% within column	30.0 %	0.0 %	19.4 %
	% of total	19.4 %	0.0 %	19.4 %
Yes	Observed	28	22	50
	% within row	56.0 %	44.0 %	100.0 %
	% within column	70.0 %	100.0 %	80.6 %
	% of total	45.2 %	35.5 %	80.6 %
Total	Observed	40	22	62
	% within row	64.5 %	35.5 %	100.0 %
	% within column	100.0 %	100.0 %	100.0 %
	% of total	64.5 %	35.5 %	100.0 %

χ^2 Tests

	Value	df	p
χ^2	8.184	1	0.004
Fisher's exact test			0.005
N	62		

Table S34 – Frequency statistics for the "*Asthma_control*" variable in the matched sample.

Frequencies of Asthma_control

Levels	Counts	% of Total	Cumulative %
Good	30	56.6 %	56.6 %
Partly	13	24.5 %	81.1 %
Poorly	10	18.9 %	100.0 %

Table S35 – Comparison between Dupilumab *vs*. Mepolizumab for quality of asthma control in the matched sample.

Contingency Tables

Asthma_control		Group: Dupilumab	Group: Mepolizumab	Total
Good	Observed	16	14	30
	% within row	53.3 %	46.7 %	100.0 %
	% within column	59.3 %	53.8 %	56.6 %
	% of total	30.2 %	26.4 %	56.6 %
Partly	Observed	5	8	13
	% within row	38.5 %	61.5 %	100.0 %
	% within column	18.5 %	30.8 %	24.5 %
	% of total	9.4 %	15.1 %	24.5 %
Poorly	Observed	6	4	10
	% within row	60.0 %	40.0 %	100.0 %
	% within column	22.2 %	15.4 %	18.9 %
	% of total	11.3 %	7.5 %	18.9 %
Total	Observed	27	26	53
	% within row	50.9 %	49.1 %	100.0 %
	% within column	100.0 %	100.0 %	100.0 %
	% of total	50.9 %	49.1 %	100.0 %

χ^2 Tests

	Value	df	p
χ^2	1.207	2	0.547
Fisher's exact test			0.566
N	53		

Table S36 – Comparison between absence/presence of complications and quality of asthma control in the matched sample.

Contingency Tables

Asthma_control		Complications: No	Complications: Yes	Total
Good	Observed	27	3	30
	% within row	90.0 %	10.0 %	100.0 %
	% within column	58.7 %	42.9 %	56.6 %
	% of total	50.9 %	5.7 %	56.6 %
Partly	Observed	12	1	13
	% within row	92.3 %	7.7 %	100.0 %
	% within column	26.1 %	14.3 %	24.5 %
	% of total	22.6 %	1.9 %	24.5 %
Poorly	Observed	7	3	10
	% within row	70.0 %	30.0 %	100.0 %
	% within column	15.2 %	42.9 %	18.9 %
	% of total	13.2 %	5.7 %	18.9 %
Total	Observed	46	7	53
	% within row	86.8 %	13.2 %	100.0 %
	% within column	100.0 %	100.0 %	100.0 %
	% of total	86.8 %	13.2 %	100.0 %

χ^2 Tests

	Value	df	p
χ^2	3.074	2	0.215
Fisher's exact test			0.255
N	53		

Table S37 – General one-way non-parametric ANOVA,
using "*Asthma control*" as grouping variable in the matched sample.

Kruskal-Wallis

	χ^2	df	p	ε^2
Age	4.184	2	0.123	0.080
Cycles_N	3.289	2	0.193	0.063
NPS	4.295	2	0.117	0.083
SNOT.22	12.839	2	0.002	0.247
SSIT.16	0.284	2	0.867	0.005
IgE_Tot	1.216	2	0.544	0.029
Eosinophil_or	5.035	2	0.081	0.101
Eosinophil_mi	4.975	2	0.083	0.096
VAS	3.355	2	0.187	0.075
Lund.Mackay	7.816	2	0.020	0.150
Access	4.723	2	0.094	0.091

ε^2: effect size.

Descriptives

	Asthma_control	Age	Cycles_N	NPS	SNOT.22	SSIT.16	IgE_Tot	Eosinophil_or	Eosinophil_mi	VAS	Lund.Mackay	Access
N	Good	30	30	30	30	30	24	29	30	27	30	30
	Partly	13	13	13	13	13	10	12	13	12	13	13
	Poorly	10	10	10	10	10	9	10	10	7	10	10
Mean	Good	58.200	2.633	5.533	50.000	4.533	315.721	557.655	556.567	39.926	17.033	13.700
	Partly	49.308	4.923	4.692	57.615	5.000	345.700	717.500	702.692	43.083	19.385	9.462
	Poorly	60.900	8.800	6.100	75.400	4.600	602.522	860.000	860.000	52.286	21.900	8.000
Median	Good	60.000	1.000	6.000	48.000	4.500	189.000	520	522.500	42	16.500	14.000
	Partly	45	1	6	55	5	183.000	550.000	525	45.500	21	6
	Poorly	61.500	3.500	6.000	78.000	5.000	251.000	875.000	875.000	51	22.000	5.500
Standard deviation	Good	15.242	5.980	1.814	17.158	2.713	575.512	292.764	287.734	16.127	5.635	8.914
	Partly	14.430	9.278	1.932	18.219	2.708	407.003	455.554	439.415	13.358	3.404	8.894
	Poorly	12.635	10.304	1.729	13.906	1.713	831.533	359.691	359.691	13.805	1.853	7.817
Minimum	Good	23	0	0	26	0	24.000	100	100	9	2	0
	Partly	23	0	0	32	1	44.000	200	200	18	14	0
	Poorly	30	0	2	56	2	14.700	300	300	31	19	0
Maximum	Good	86	24	8	92	10	2886.000	1500	1500	69	28	24
	Partly	71	30	6	96	10	1269.000	1690	1690	64	24	24
	Poorly	81	25	8	98	8	2287.000	1310	1310	76	24	24

Table S38 – The *post-hoc* pairwise comparisons for significant variables at the general one-way non-parametric ANOVA model for "*Asthma control*" in the matched sample.

Pairwise comparisons - SNOT.22

		W	p
Good	Partly	1.722	0.443
Good	Poorly	4.840	0.002
Partly	Poorly	3.555	0.032

Pairwise comparisons - Lund.Mackay

		W	p
Good	Partly	1.806	0.408
Good	Poorly	3.705	0.024
Partly	Poorly	2.445	0.195

Table S39 – Frequency statistics for the "*EPOS_SIX*" and "*EPOS_TWELVE*" variables in the matched sample.

Frequencies of EPOS_SIX

Levels	Counts	% of Total	Cumulative %
Good	40	87.0 %	87.0 %
Moderate	4	8.7 %	95.7 %
Poor	2	4.3 %	100.0 %

Frequencies of EPOS_TWELVE

Levels	Counts	% of Total	Cumulative %
Good	38	92.7 %	92.7 %
Moderate	2	4.9 %	97.6 %
Poor	1	2.4 %	100.0 %

Table S40 – Comparison between Dupilumab *vs*. Mepolizumab for "*EPOS_SIX*" variable in the matched sample.

Contingency Tables

EPOS_SIX		Group: Dupilumab	Group: Mepolizumab	Total
Good	Observed	27	13	40
	% within row	67.5 %	32.5 %	100.0 %
	% within column	90.0 %	81.3 %	87.0 %
	% of total	58.7 %	28.3 %	87.0 %
Moderate	Observed	2	2	4
	% within row	50.0 %	50.0 %	100.0 %
	% within column	6.7 %	12.5 %	8.7 %
	% of total	4.3 %	4.3 %	8.7 %
Poor	Observed	1	1	2
	% within row	50.0 %	50.0 %	100.0 %
	% within column	3.3 %	6.3 %	4.3 %
	% of total	2.2 %	2.2 %	4.3 %
Total	Observed	30	16	46
	% within row	65.2 %	34.8 %	100.0 %
	% within column	100.0 %	100.0 %	100.0 %
	% of total	65.2 %	34.8 %	100.0 %

χ^2 Tests

	Value	df	p
χ^2	0.704	2	0.703
Fisher's exact test			0.650
N	46		

Table S41 – Comparison between Dupilumab *vs*. Mepolizumab for "*EPOS_TWELVE*" variable in the matched sample.

Contingency Tables

EPOS_TWELVE		Group: Dupilumab	Group: Mepolizumab	Total
Good	Observed	27	11	38
	% within row	71.1 %	28.9 %	100.0 %
	% within column	93.1 %	91.7 %	92.7 %
	% of total	65.9 %	26.8 %	92.7 %
Moderate	Observed	1	1	2
	% within row	50.0 %	50.0 %	100.0 %
	% within column	3.4 %	8.3 %	4.9 %
	% of total	2.4 %	2.4 %	4.9 %
Poor	Observed	1	0	1
	% within row	100.0 %	0.0 %	100.0 %
	% within column	3.4 %	0.0 %	2.4 %
	% of total	2.4 %	0.0 %	2.4 %
Total	Observed	29	12	41
	% within row	70.7 %	29.3 %	100.0 %
	% within column	100.0 %	100.0 %	100.0 %
	% of total	70.7 %	29.3 %	100.0 %

χ^2 Tests

	Value	df	p
χ^2	0.831	2	0.660
Fisher's exact test			0.657
N	41		

S6.4 – Box-violin plot for Dupilumab group obtained after propensity score analysis

Considering matching sample obtained with the propensity score analysis (see Section S5, pp. 29-45), where n = 10 patients of the only Dupilumab group were discharged, we have reproduced Figure 1 of the paper (box-violin plots at each time-point for Dupilumab group) (**Figure S14**). The extended descriptive statistics at each time point are presented in **Table S42**.

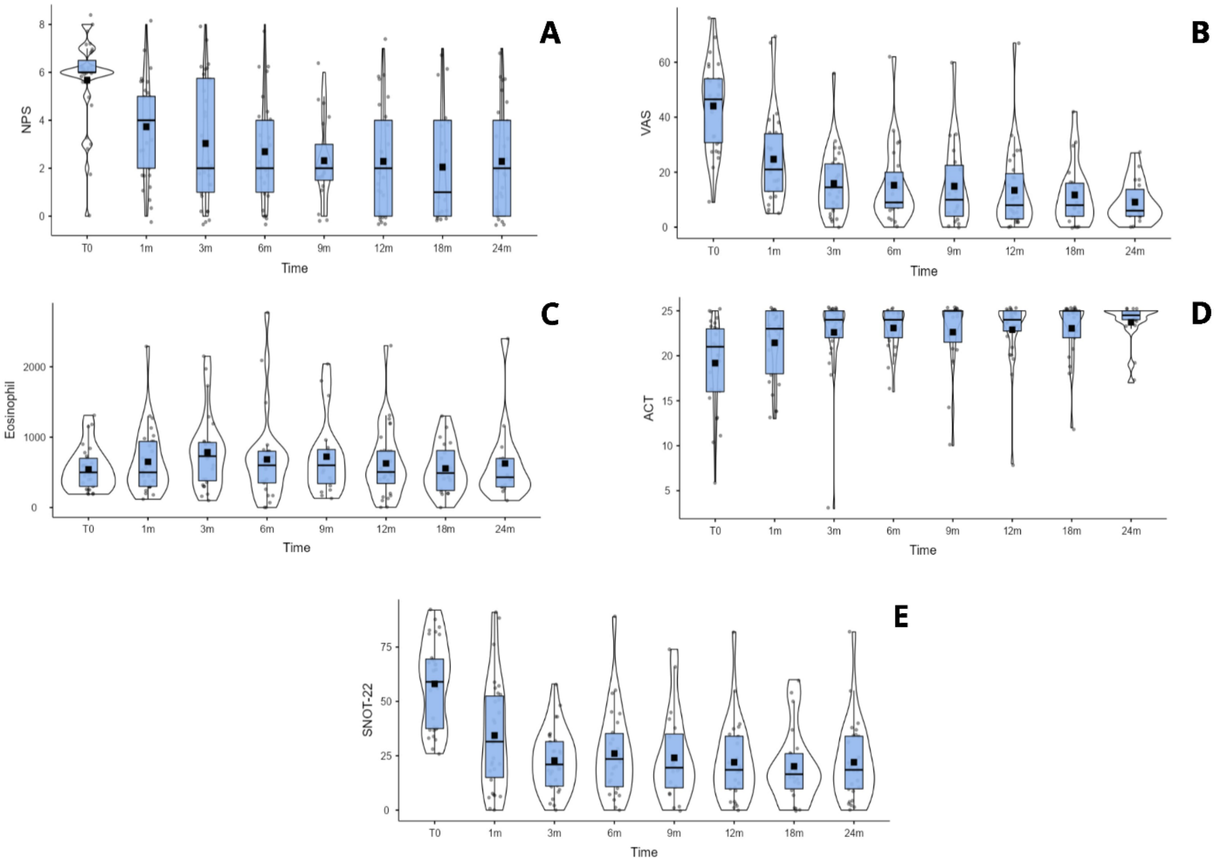

Figure S14 – Box-violin plots for Dupilumab main clinical outcomes over time for the n = 31 patients selected by the propensity score analysis: Nasal Polyp Score (NPS) **(A)**; Visual Analogue Scale (VAS) **(B)**; Eosinophil count (cells/µL) **(C)**; Asthma Control Test (ACT) **(D)**; sinonasal outcome test- 22 (SNOT-22) **(E)**.

Table S42 – Extended descriptive statistics for continuous variables at each time-point of Figure S14.

	Time	NPS	VAS	Eosinophil	ACT	SNOT-22
**Mean**	T0	5.677	44.071	542.828	19.185	58.065
	1m	3.733	24.720	649.966	21.440	34.333
	3m	3.033	15.893	786.667	22.607	22.774
	6m	2.692	15.261	683.684	23.087	26.000
	9m	2.316	14.895	723.158	22.632	24.050
	12m	2.286	13.407	627.164	22.893	22.000
	18m	2.048	11.714	555.950	23.048	20.136
	24m	2.286	9.111	627.143	23.722	22.000
**Std. Error mean**	T0	0.319	2.966	57.199	0.983	3.580
	1m	0.380	3.385	85.945	0.800	4.612
	3m	0.456	2.276	101.022	0.815	2.555
	6m	0.429	2.968	125.956	0.499	3.960
	9m	0.398	3.541	125.127	0.965	4.553
	12m	0.430	2.793	94.702	0.665	3.460
	18m	0.509	2.480	79.350	0.738	3.634
	24m	0.430	1.797	156.347	0.523	3.460
**95% CI mean lower bound**	T0	5.052	38.259	430.720	17.259	51.048
	1m	2.988	18.085	481.517	19.872	25.295
	3m	2.139	11.432	588.667	21.009	17.766
	6m	1.852	9.443	436.816	22.110	18.238
	9m	1.536	7.955	477.914	20.740	15.125
	12m	1.443	7.934	441.552	21.589	15.218
	18m	1.049	6.854	400.427	21.601	13.014
	24m	1.443	5.590	320.708	22.698	15.218
**95% CI mean upper bound**	T0	6.303	49.884	654.935	21.111	65.081
	1m	4.479	31.355	818.414	23.008	43.372
	3m	3.927	20.354	984.666	24.205	27.783
	6m	3.533	21.079	930.553	24.064	33.762
	9m	3.095	21.835	968.402	24.523	32.975
	12m	3.128	18.881	812.777	24.197	28.782
	18m	3.046	16.574	711.473	24.495	27.259
	24m	3.128	12.633	933.578	24.747	28.782
**Median**	T0	6	46.500	500.000	21	59
	1m	4.000	21	500.000	23	31.500
	3m	2.000	14.500	730.000	24.000	21
	6m	2.000	9	600.000	24	23.500
	9m	2	10	600.000	25	19.500
	12m	2.000	8	505.000	24.000	18.500
	18m	1	8	490.000	25	16.500

	Time	NPS	VAS	Eosinophil	ACT	SNOT-22
	24m	2.000	6.000	430.000	24.500	18.500
**Standard deviation**	T0	1.777	15.694	308.024	5.107	19.933
	1m	2.083	16.925	462.828	4.001	25.259
	3m	2.498	12.044	524.925	4.315	14.228
	6m	2.187	14.236	629.778	2.392	20.193
	9m	1.734	15.434	545.416	4.206	20.364
	12m	2.275	14.511	501.117	3.521	18.310
	18m	2.334	11.363	354.864	3.383	17.044
	24m	2.275	7.623	584.997	2.218	18.310
**IQR**	T0	0.500	23.250	400.000	7.000	32.000
	1m	3.000	21.000	640.000	7.000	37.500
	3m	4.750	16.250	545.000	3.000	20.500
	6m	3.000	13.000	450.000	3.000	24.500
	9m	1.500	18.500	485.000	3.500	24.750
	12m	4.000	16.500	460.000	2.250	24.250
	18m	4.000	12.000	567.500	3.000	16.250
	24m	4.000	9.750	405.000	1.000	24.250
**Range**	T0	8	67	1120.000	19	66
	1m	8	64	2171.000	12	91
	3m	8	56	2050.000	22	58
	6m	8	62	2769.430	9	89
	9m	6	60	1910.000	15	74
	12m	7	67	2296.400	17	82
	18m	7	42	1300.000	13	60
	24m	7	27	2300.000	8	82
**Minimum**	T0	0	9	190.000	6	26
	1m	0	5	119.000	13	0
	3m	0	0	100.000	3	0
	6m	0	0	0.570	16	0
	9m	0	0	130.000	10	0
	12m	0	0	3.600	8	0
	18m	0	0	0.000	12	0
	24m	0	0	100.000	17	0
**Maximum**	T0	8	76	1310.000	25	92
	1m	8	69	2290.000	25	91
	3m	8	56	2150.000	25	58
	6m	8	62	2770.000	25	89
	9m	6	60	2040.000	25	74
	12m	7	67	2300.000	25	82
	18m	7	42	1300.000	25	60
	24m	7	27	2400.000	25	82
**Skewness**	T0	-1.531	-0.141	0.983	-0.964	-0.054
	1m	-0.036	1.286	1.739	-1.002	0.605

	Time	NPS	VAS	Eosinophil	ACT	SNOT-22
	3m	0.358	1.345	1.122	-3.744	0.464
	6m	0.676	1.832	2.006	-1.488	1.273
	9m	0.669	1.556	1.311	-2.140	1.156
	12m	0.656	2.125	1.459	-3.090	1.415
	18m	0.823	1.256	0.542	-2.117	1.048
	24m	0.656	0.989	2.404	-2.372	1.415
**Std. Errors kewness**	T0	0.421	0.441	0.434	0.448	0.421
	1m	0.427	0.464	0.434	0.464	0.427
	3m	0.427	0.441	0.448	0.441	0.421
	6m	0.456	0.481	0.464	0.481	0.456
	9m	0.524	0.524	0.524	0.524	0.512
	12m	0.441	0.448	0.441	0.441	0.441
	18m	0.501	0.501	0.512	0.501	0.491
	24m	0.441	0.536	0.597	0.536	0.441
**Kurtosis**	T0	2.711	-0.336	0.409	0.258	-1.269
	1m	-0.678	1.683	4.317	-0.432	-0.336
	3m	-1.229	3.101	1.117	16.504	-0.131
	6m	-0.107	4.148	4.775	2.129	2.384
	9m	-0.167	2.751	1.082	4.252	0.963
	12m	-0.862	6.128	3.252	11.762	2.998
	18m	-0.560	1.307	-0.507	4.794	0.557
	24m	-0.862	0.308	6.731	5.272	2.998
**Std. Error kurtosis**	T0	0.821	0.858	0.845	0.872	0.821
	1m	0.833	0.902	0.845	0.902	0.833
	3m	0.833	0.858	0.872	0.858	0.821
	6m	0.887	0.935	0.902	0.935	0.887
	9m	1.014	1.014	1.014	1.014	0.992
	12m	0.858	0.872	0.858	0.858	0.858
	18m	0.972	0.972	0.992	0.972	0.953
	24m	0.858	1.038	1.154	1.038	0.858
**Shapiro-Wilk (*W*)**	T0	0.800	0.978	0.905	0.905	0.931
	1m	0.967	0.881	0.847	0.811	0.938
	3m	0.900	0.901	0.897	0.555	0.973
	6m	0.924	0.822	0.792	0.801	0.912
	9m	0.877	0.847	0.854	0.644	0.889
	12m	0.869	0.785	0.885	0.625	0.888
	18m	0.828	0.873	0.952	0.660	0.890
	24m	0.869	0.902	0.733	0.621	0.888
**Shapiro-Wilk (*P* value)**	T0	< .001	0.810	0.013	0.017	0.046
	1m	0.460	0.007	< .001	< .001	0.078
	3m	0.008	0.012	0.012	< .001	0.596
	6m	0.054	< .001	< .001	< .001	0.029
	9m	0.019	0.006	0.008	< .001	0.026
	12m	0.002	< .001	0.005	< .001	0.006

	Time	NPS	VAS	Eosinophil	ACT	SNOT-22
	18m	0.002	0.011	0.402	< .001	0.019
	24m	0.002	0.062	< .001	< .001	0.006

Section S7. Evaluation of complications in both unmatched and matched samples

The occurrence of complications during treatment represents a topic worthy of further investigation. In a first phase we had made a distinction between early (within the first 30 days of starting treatment) and late (after the first 30 days of starting treatment) complications. However, only $n = 5$ early complications were observed in all cases, making this low number of events unsuitable for predictive regression models[6]. The late complications were $n = 9$. Although this number of events was more acceptable, the whole complications were $n = 12$, making the variable "*Complications*" (early and/or late complications) the best option to enter as dependent variable in logistic regression models. The whole number of complications does not correspond to the sum of early and late complications, because in some patients both early and late complications occurred. Descriptive statistics for early/late/whole complications in the original (unmatched) patient population are preliminarily reported in **Table S43**, where in **Table S44** the contingency tables for treatments are shown. In Table S44 also odds ratio, relative risk and their corresponding 95% confidence intervals (CI) have been calculated. For more details about the follow-up time-points, see "*Dupilumab and Mepolizumab Administration and Follow-up*" section in the paper.

Table S43 – Number of complications observed in the unmatched sample.

Frequencies of EarlyCompl

Levels	Counts	% of Total	Cumulative %
No	67	93.1 %	93.1 %
Yes	5	6.9 %	100.0 %

Frequencies of LateCompl

Levels	Counts	% of Total	Cumulative %
No	63	87.5 %	87.5 %
Yes	9	12.5 %	100.0 %

Frequencies of Complications

Levels	Counts	% of Total	Cumulative %
No	60	83.3 %	83.3 %
Yes	12	16.7 %	100.0 %

[6] For more details about the minimum number of events in regression models, see, e.g., Santori G. et al. *Application and validation of Cox regression models in a single-center series of double kidney transplantation*. Transplant Proc. 2010;42(4):1098-103, and related references.

Table S44 (Part I) – Comparison between Dupilumab *vs*. Mepolizumab for early complications in the unmatched sample.

Contingency Tables

EarlyCompl		Group: Dupilumab	Group: Mepolizumab	Total
No	Observed	36	31	67
	% within row	53.7 %	46.3 %	100.0 %
	% within column	87.8 %	100.0 %	93.1 %
	% of total	50.0 %	43.1 %	93.1 %
Yes	Observed	5	0	5
	% within row	100.0 %	0.0 %	100.0 %
	% within column	12.2 %	0.0 %	6.9 %
	% of total	6.9 %	0.0 %	6.9 %
Total	Observed	41	31	72
	% within row	56.9 %	43.1 %	100.0 %
	% within column	100.0 %	100.0 %	100.0 %
	% of total	56.9 %	43.1 %	100.0 %

χ^2 Tests

	Value	df	p
χ^2	4.063	1	0.044
Fisher's exact test			0.066
N	72		

Comparative Measures

	Value	95% Confidence Intervals: Lower	95% Confidence Intervals: Upper
Odds ratio	0.105 [a]	0.006	1.981
Relative risk	0.537 [b]	0.430	0.671

[a] Haldane-Ascombe correction applied

[b] rows compared

Table S44 (Part II) – Comparison between Dupilumab *vs*. Mepolizumab for late complications in the unmatched sample.

Contingency Tables

LateCompl		Group: Dupilumab	Group: Mepolizumab	Total
No	Observed	34	29	63
	% within row	54.0 %	46.0 %	100.0 %
	% within column	82.9 %	93.5 %	87.5 %
	% of total	47.2 %	40.3 %	87.5 %
Yes	Observed	7	2	9
	% within row	77.8 %	22.2 %	100.0 %
	% within column	17.1 %	6.5 %	12.5 %
	% of total	9.7 %	2.8 %	12.5 %
Total	Observed	41	31	72
	% within row	56.9 %	43.1 %	100.0 %
	% within column	100.0 %	100.0 %	100.0 %
	% of total	56.9 %	43.1 %	100.0 %

χ^2 Tests

	Value	df	p
χ^2	1.821	1	0.177
Fisher's exact test			0.283
N	72		

Comparative Measures

	Value	95% Confidence Intervals: Lower	95% Confidence Intervals: Upper
Odds ratio	0.335	0.064	1.740
Relative risk	0.694 [a]	0.457	1.053

[a] rows compared

Table S44 (Part III) – Comparison between Dupilumab *vs*. Mepolizumab for overall complications (early and/or late) in the unmatched sample.

Contingency Tables

		Group		
Complications		Dupilumab	Mepolizumab	Total
No	Observed	31	29	60
	% within row	51.7 %	48.3 %	100.0 %
	% within column	75.6 %	93.5 %	83.3 %
	% of total	43.1 %	40.3 %	83.3 %
Yes	Observed	10	2	12
	% within row	83.3 %	16.7 %	100.0 %
	% within column	24.4 %	6.5 %	16.7 %
	% of total	13.9 %	2.8 %	16.7 %
Total	Observed	41	31	72
	% within row	56.9 %	43.1 %	100.0 %
	% within column	100.0 %	100.0 %	100.0 %
	% of total	56.9 %	43.1 %	100.0 %

χ^2 Tests

	Value	df	p
χ^2	4.090	1	0.043
Fisher's exact test			0.057
N	72		

Comparative Measures

		95% Confidence Intervals	
	Value	Lower	Upper
Odds ratio	0.214	0.043	1.059
Relative risk	0.620 [a]	0.436	0.882

[a] rows compared

Descriptive statistics for early/late/whole complications in the matched patient population are reported in **Table S45**, where in **Table S46** the contingency tables for treatments are shown. In Table S46 also odds ratio, relative risk and their respective 95% CI have been calculated.

Table S45 – Number of complications observed in the matched sample.

Frequencies of EarlyCompl

Levels	Counts	% of Total	Cumulative %
No	58	93.5 %	93.5 %
Yes	4	6.5 %	100.0 %

Frequencies of LateCompl

Levels	Counts	% of Total	Cumulative %
No	56	90.3 %	90.3 %
Yes	6	9.7 %	100.0 %

Frequencies of Complications

Levels	Counts	% of Total	Cumulative %
No	54	87.1 %	87.1 %
Yes	8	12.9 %	100.0 %

Table S46 (Part I) – Comparison between Dupilumab *vs*. Mepolizumab for early complications in the matched sample.

Contingency Tables

EarlyCompl		Group		Total
		Dupilumab	Mepolizumab	
No	Observed	27	31	58
	% within row	46.6 %	53.4 %	100.0 %
	% within column	87.1 %	100.0 %	93.5 %
	% of total	43.5 %	50.0 %	93.5 %
Yes	Observed	4	0	4
	% within row	100.0 %	0.0 %	100.0 %
	% within column	12.9 %	0.0 %	6.5 %
	% of total	6.5 %	0.0 %	6.5 %
Total	Observed	31	31	62
	% within row	50.0 %	50.0 %	100.0 %
	% within column	100.0 %	100.0 %	100.0 %
	% of total	50.0 %	50.0 %	100.0 %

χ^2 Tests

	Value	df	p
χ^2	4.276	1	0.039
Fisher's exact test			0.113
N	62		

Comparative Measures

	Value	95% Confidence Intervals	
		Lower	Upper
Odds ratio	0.097 [a]	0.005	1.884
Relative risk	0.466 [b]	0.353	0.613

[a] Haldane-Ascombe correction applied

[b] rows compared

Table S46 (Part II) – Comparison between Dupilumab *vs*. Mepolizumab for late complications in the matched sample.

Contingency Tables

LateCompl		Group: Dupilumab	Group: Mepolizumab	Total
No	Observed	27	29	56
	% within row	48.2 %	51.8 %	100.0 %
	% within column	87.1 %	93.5 %	90.3 %
	% of total	43.5 %	46.8 %	90.3 %
Yes	Observed	4	2	6
	% within row	66.7 %	33.3 %	100.0 %
	% within column	12.9 %	6.5 %	9.7 %
	% of total	6.5 %	3.2 %	9.7 %
Total	Observed	31	31	62
	% within row	50.0 %	50.0 %	100.0 %
	% within column	100.0 %	100.0 %	100.0 %
	% of total	50.0 %	50.0 %	100.0 %

χ^2 Tests

	Value	df	p
χ^2	0.738	1	0.390
Fisher's exact test			0.671
N	62		

Comparative Measures

	Value	95% Confidence Intervals: Lower	95% Confidence Intervals: Upper
Odds ratio	0.466	0.079	2.751
Relative risk	0.723 [a]	0.386	1.355

[a] rows compared

Table S46 (Part III) – Comparison between Dupilumab *vs*. Mepolizumab for overall complications (early and/or late) in the matched sample.

Contingency Tables

		Group		
Complications		Dupilumab	Mepolizumab	Total
No	Observed	25	29	54
	% within row	46.3 %	53.7 %	100.0 %
	% within column	80.6 %	93.5 %	87.1 %
	% of total	40.3 %	46.8 %	87.1 %
Yes	Observed	6	2	8
	% within row	75.0 %	25.0 %	100.0 %
	% within column	19.4 %	6.5 %	12.9 %
	% of total	9.7 %	3.2 %	12.9 %
Total	Observed	31	31	62
	% within row	50.0 %	50.0 %	100.0 %
	% within column	100.0 %	100.0 %	100.0 %
	% of total	50.0 %	50.0 %	100.0 %

χ^2 Tests

	Value	df	p
χ^2	2.296	1	0.130
Fisher's exact test			0.255
N	62		

Comparative Measures

		95% Confidence Intervals	
	Value	Lower	Upper
Odds ratio	0.287	0.053	1.553
Relative risk	0.617 [a]	0.377	1.010

[a] rows compared

The results of univariate logistic regression performed on the unmatched sample are reported in **Table S47**.

Table S47 – Univariate logistic regression models performed with overall complications as dependent variable in the unmatched sample.

**Variable**	**Estimate (β)***	**Odds ratio (95% CI)**	***P* value**
Group [Dupilumab]	1.543	4.677 (0.944 – 23.176)	0.059
Age	0.025	1.026 (0.983 – 1.070)	0.241
Gender [Female]	1.543	4.677 (0.944 – 23.176)	0.059
NSAID intolerance [Yes]	1.240	3.455 (0.932 – 12.802)	0.064
Previous surgery [Yes]	1.386	4.000 (0.477 – 33.510)	0.201
Cycle_N	- 0.091	0.913 (0.855 – 0.975)	0.007
Allergy [Yes]	1.922	6.838 (0.827 – 56.505)	0.074
Asthma [Yes]	0.223	1.250 (0.241 – 6.474)	0.790
Smoke [Yes]	- 0.788	0.455 (0.053 – 3.927)	0.474
NPS	- 0.107	0.898 (0.594 – 1.359)	0.612
SNOT-22	- 0.025	0.975 (0.944 – 1.008)	0.135
SSIT-16	0.094	1.098 (0.845 – 1.426)	0.484
IgE Tot	0.000	1.000 (0.998 – 1.002)	0.572
Eosinophil_or	- 0.000	1.000 (0.998 – 1.002)	0.823
Eosinophil_mi	- 0.000	1.000 (0.998 – 1.002)	0.856
ACT	0.118	1.126 (0.995 – 1.273)	0.060
VAS	- 0.029	0.972 (0.928 – 1.018)	0.227
Lund-Mackay	- 0.149	0.861 (0.720 – 1.030)	0.102
Access	0.102	1.108 (1.002 – 1.225)	0.046

* Estimate represents the log odds of "*Complications = No (0)*" *vs*. "*Complications = Yes (1)*". A negative coefficient ($\beta < 0$) for a continuous variable indicates an inverse relationship: as the predictor variable increases, the log-odds and probability of the outcome event ($Y = 1$) decrease. This means that higher values of the variable are associated with a lower likelihood of the event occurring.

NSAID: non steroid anti-inflammatory drugs; Cycle_N: Number of systemic corticosteroids cycles in the previous year; SNOT-22: Sinonasal Outcome Test-22; SSIT-16: Sniffin' Sticks-16 Identification Test; ACT: Asthma Control Test; NPS: Nasal Polyp Score.

The univariate logistic regression for putative independent predictors of complications in the unmatched sample has returned two significative variables ("*Cycle_N*" and "*Access*"), while in other five variables a *P* value < 0.1 was calculated ("*Group*", "*Gender*", "*NSAID_intol*", "*Allergy*", and "*ACT*"). The significative models had an accuracy of 0.833 ("*Cycle_N*") and 0.831 ("*Access*"), respectively. However, "within" accuracy was severely unbalanced considering specificity (Sp) and sensitivity (Sn) of each model [*Cycle_N*: Sp = 0.083, Sn = 0.983, Area under the ROC curve (AUC) = 0.667; *Access*: Sp = 0.000, Sn = 1.000, AUC = 0.666].

In the subsequent multivariate logistic regression model (MLR1) performed by entering only the significative variables at the univariate analysis, the "*Cycle_N*" variable confirmed a *P* value < 0.05 (**Table S48**).

Table S48 – Multivariate logistic regression model (MLR1) performed with overall complications as dependent variable in the unmatched sample, by entering the significative variables at the univariate analysis.

**Variable**	**Estimate (β)**	**Odds ratio (95% CI)**	***P* value**
Cycle_N	- 0.090	0.914 (0.852 – 0.980)	0.012
Access	0.102	1.107 (0.996 – 1.231)	0.060

Further MLR1 outputs are shown in **Figure S15**.

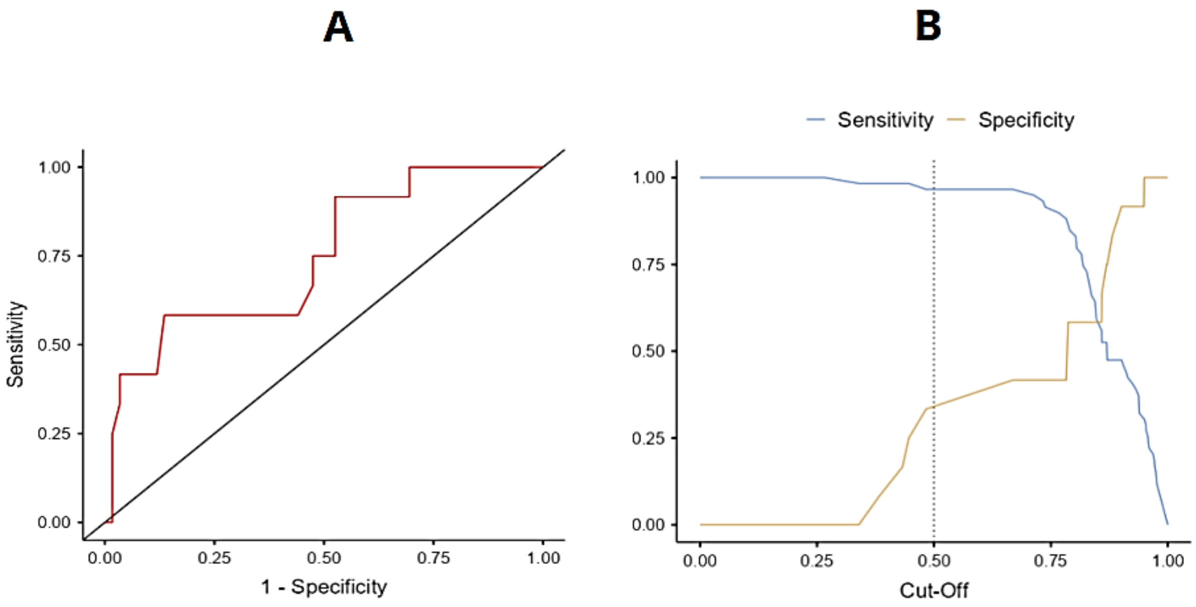

Classification Table – Complications

	Predicted		
Observed	Yes	No	% Correct
Yes	5	7	41.667
No	2	57	96.610

Note. The cut-off value is set to 0.5

Predictive Measures

Accuracy	Specificity	Sensitivity	AUC
0.873	0.417	0.966	0.746

Note. The cut-off value is set to 0.5

Figure S15 – ROC curve (A), cut-off plot (B), classification table and predictive measures of the MLR1.

Finally, a second multivariate logistic regression model (MLR2), evaluated by entering also the variables with *P* value < 0.1 at the univariate analysis, has returned the results reported in **Table S49**.

Table S49 – Multivariate logistic regression model (MLR2) performed with overall complications as dependent variable in the unmatched sample, by entering the variables with *P* value <0.1 at the univariate analysis.

**Variable**	**Estimate (*β*)**	**Odds ratio (95% CI)**	***P* value**
Cycle_N	- 0.156	0.855 (0.735 – 0.996)	0.044
Access	0.329	1.389 (1.050 – 1.838)	0.021
Group [Dupilumab]	3.830	46.084 (1.080 – 1966)	0.045
Gender [Female]	6.417	612.195 (3.406 – 110022)	0.002
NSAID intolerance [Yes]	4.157	63.910 (0.906 – 4509)	0.056
Allergy	- 1.396	0.248 (0.007 – 9.383)	0.452
ACT	0.074	01.077 (0.865 – 1.341)	0.509

In the MLR2, a *P* value < 0.05 was returned by the "*Cycle_N*", "*Access*", "*Group*", and "*Gender*" variables, whereas in the "*NSAID_intol*" variable (*P* < 0.056) the high odds ratio and its very large 95% CI should suggest caution. Notably, huge odds ratio and wide 95% CI occurred also in "*Group*" and "*Gender*" variables. Limited to the unmatched sample, treatment with Dupilumab in female patients seems to represent a potential higher risk pattern for the onset of complications. The other MLR2 outputs are shown in **Figure S16**.

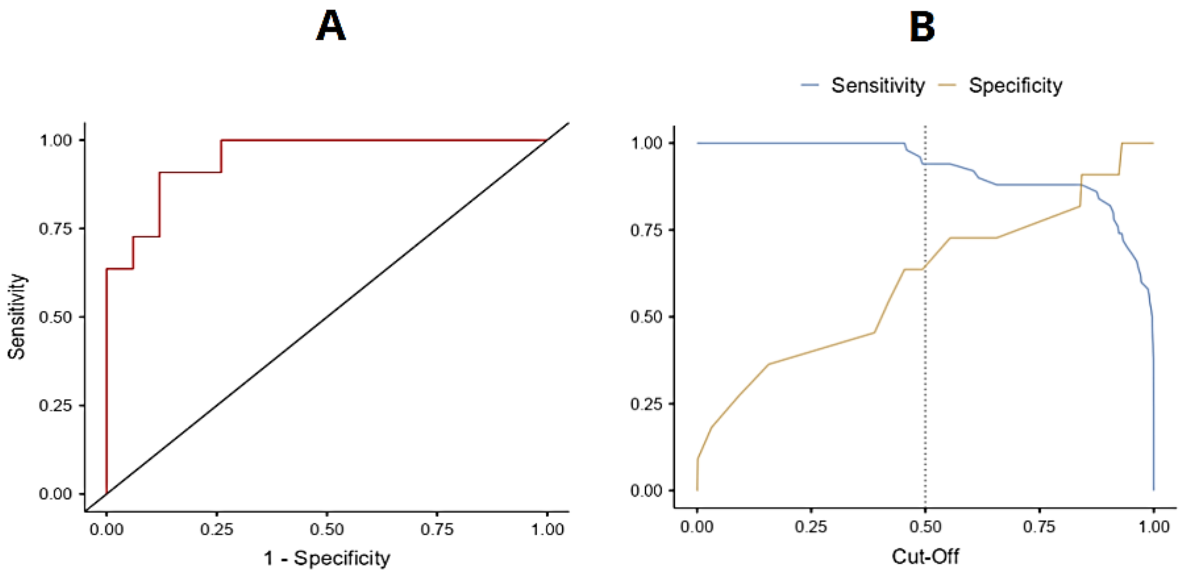

Classification Table – Complications

	Predicted		
Observed	Yes	No	% Correct
Yes	8	3	72.727
No	3	47	94.000

Note. The cut-off value is set to 0.5

Predictive Measures

Accuracy	Specificity	Sensitivity	AUC
0.902	0.727	0.940	0.949

Note. The cut-off value is set to 0.5

Figure S16 – ROC curve (A), cut-off plot (B), classification table and predictive measures of the MLR2.

The results of univariate logistic regression performed on the matched sample are reported in **Table S50**.

Table S50 – Univariate logistic regression models performed with overall complications as dependent variable in the matched sample.

**Variable**	**Estimate (*β*)***	**Odds ratio (95% CI)**	***P* value**
Group [Dupilumab]	1.247	3.480 (0.644 – 18.809)	0.147
Age	0.028	1.029 (0.978 – 1.082)	0.276
Gender [Female]	1.797	6.034 (0.694 – 52.456)	0.103
NSAID intolerance [Yes]	1.289	3.627 (0.776 – 16.959)	0.102
Previous surgery [Yes]	1.081	2.947 (0.335 – 25.947)	0.330
Cycle_N	- 0.058	0.944 (0.872 – 1.021)	0.149
Allergy [Yes]	1.494	4.455 (0.511 – 38.833)	0.176
Asthma [Yes]	- 0.383	0.682 (0.120 – 3.890)	0.666
Smoke [Yes]	- 0.464	0.629 (0.069 – 5.699)	0.680
NPS	- 0.035	0.965 (0.626 – 1.490)	0.874
SNOT-22	- 0.017	0.983 (0.994 – 1.023)	0.393
SSIT-16	0.004	1.004 (0.747 – 1.351)	0.977
IgE Tot	0.000	1.000 (0.999 – 1.002)	0.685
Eosinophil_or	- 0.000	0.999 (0.997 – 1.001)	0.604
Eosinophil_mi	- 0.000	0.999 (0.997 – 1.001)	0.580
ACT	0.077	1.080 (0.927 – 1.258)	0.326
VAS	- 0.019	0.981 (0.927 – 1.039)	0.515
Lund-Mackay	- 0.205	0.815 (0.647 – 1.026)	0.081
Access	0.082	1.086 (0.975 – 1.209)	0.135

* Estimate represents the log odds of "*Complications = No (0)*" *vs*. "*Complications = Yes (1)*". A negative coefficient ($\beta < 0$) for a continuous variable indicates an inverse relationship: as the predictor variable increases, the log-odds and probability of the outcome event ($Y = 1$) decrease. This means that higher values of the variable are associated with a lower likelihood of the event occurring.

NSAID: non steroid anti-inflammatory drugs; Cycle_N: Number of systemic corticosteroids cycles in the previous year; SNOT-22: Sinonasal Outcome Test-22; SSIT-16: Sniffin' Sticks-16 Identification Test; ACT: Asthma Control Test; NPS: Nasal Polyp Score.

No statistical significance was found in the univariate logistic regression performed in the matched sample for overall complications. Only the "*Lund-Mackay*" variable has produced a *P* value < 0.1.

To perform statistical analysis in the paper, as well as in this supplementary report, the following R packages were used:

```
R version 4.5.2 (2025-10-31 ucrt)
Platform: x86_64-w64-mingw32/x64
Running under: Windows 11 x64 (build 26200)


# attached base packages:
[1] stats  graphics  grDevices  utils  datasets  methods  base

# other attached packages:
[1] pacman_0.5.1     sandwich_3.1-1   modelsummary_2.6.0  broom_1.0.12
[5] ggplot2_4.0.2    MatchIt_4.7.2    WeightIt_1.6.0      cobalt_4.6.2

# loaded via a namespace (and not attached):
 [1] gtable_0.3.6          dplyr_1.2.0         compiler_4.5.2
 [4] tidyselect_1.2.1      Rcpp_1.1.1          dichromat_2.0-0.1
 [7] tidyr_1.3.2           scales_1.4.0        fastmap_1.2.0
[10] lattice_0.22-9        R6_2.6.1            tables_0.9.33
[13] generics_0.1.4        knitr_1.51          backports_1.5.0
[16] tibble_3.3.1          chk_0.10.0          pillar_1.11.1
[19] RColorBrewer_1.1-3    rlang_1.1.7         xfun_0.56
[22] S7_0.2.1              otel_0.2.0          cli_3.6.5
[25] withr_3.0.2           magrittr_2.0.4      digest_0.6.39
[28] grid_4.5.2            lifecycle_1.0.5     vctrs_0.7.1
[31] evaluate_1.0.5        jmv 2.7.7.          glue_1.8.0
[34] farver_2.1.2          zoo_1.8-15          purrr_1.2.1
[37] tools_4.5.2           pkgconfig_2.0.3     htmltools_0.5.9
[40] data.table_1.18.2.1
```
